# Non-invasive identification of protein biomarkers for early pregnancy diagnosis in the cheetah (*Acinonyx jubatus*)

**DOI:** 10.1371/journal.pone.0188575

**Published:** 2017-12-13

**Authors:** Diana C. Koester, David E. Wildt, Morgan Maly, Pierre Comizzoli, Adrienne E. Crosier

**Affiliations:** 1 Center for Species Survival, Smithsonian Conservation Biology Institute, Front Royal, VA, United States of America; 2 Center for Species Survival, National Zoological Park, Washington, DC, United States of America; Universidade Federal do Parana, BRAZIL

## Abstract

Approximately 80% of cheetahs living in typical zoological collections never reproduce. In more than 60% of breedings, the female is confirmed to ovulate, but parturition fails to occur. It is unknown if these non-pregnant intervals of elevated progesterone (deemed luteal phases) are conception failures or a pregnancy terminating in embryonic/fetal loss. There have been recent advances in metabolic profiling and proteome analyses in many species with mass spectrometry used to identify ‘biomarkers’ and mechanisms indicative of specific physiological states (including pregnancy). Here, we hypothesized that protein expression in voided cheetah feces varied depending on pregnancy status. We: 1) identified the expansive protein profile present in fecal material of females; and 2) isolated proteins that may be candidates playing a role in early pregnancy establishment and diagnosis. Five hundred and seventy unique proteins were discovered among samples from pregnant (n = 8), non-pregnant, luteal phase (n = 5), and non-ovulatory control (n = 5) cheetahs. Four protein candidates were isolated that were significantly up-regulated and two were down-regulated in samples from pregnant compared to non-pregnant or control counterparts. One up-regulated candidate, immunoglobulin J chain (IGJ; an important component of the secretory immune system) was detected using a commercially available antibody via immunoblotting. Findings revealed that increased IGJ abundance could be used to detect pregnancy successfully in >80% of 23 assessed females within 4 weeks after mating. The discovery of a novel fecal pregnancy marker improves the ability to determine reproductive, especially gestational, status in cheetahs managed in an *ex situ* insurance and source population.

## Introduction

Cheetah (*Acinonyx jubatus*) numbers continue to decline markedly in nature, with a recent, highly publicized report predicting only ~7,100 individuals remaining in fragmented habitat throughout continental Africa [1]. In the face of these withering decreases in nature, it is prudent to create a sustainable, *ex situ* cheetah population [2]. Such a collection can serve as insurance and for research (for studies that cannot be conducted on elusive, wild animals) and public awareness, including inspiring people to support *in situ* conservation. However, the captive cheetah population has never been self-sustaining, with more animals dying every year than being born [3]. The species is challenging to reproduce, with females having short waves of follicular activity (rather than a true estrous cycle), often punctuated with inexplicable periods of ovarian acyclicity [4]. Unlike the human and most commonly studied livestock species that ‘spontaneously’ ovulate [5], the cheetah is an induced ovulator, that is, mating or exogenous hormone therapy is required for ovarian ova release [4]. One particular oddity is that copulations (even with a proven breeder male) often fail to produce offspring [6]. For example, more than 60% of matings recorded to occur in North American zoos since 2013 have not resulted in parturition. These cheetahs often are referred to as ‘pseudopregnant’, an ambiguous term since there is no scientific knowledge yet about intra-oviductal/intra-uterine events post-mating. However, it is known that virtually all copulating females are ovulating based on physical observations of ovarian luteal tissue [7, 8] that produces progestagens detectable in a rising and then falling profile within voided feces [4]. However, there are no differences in the concentrations or temporal patterns of progestagen metabolites between pregnant and non-pregnant females for at least 55 d post-mating (with parturition normally occurring at 94 d) [4]. Therefore, there are no distinguishing hormonal features between a gravid and non-gravid cheetah until late in gestation.

Although data are largely unavailable for felids, maternal recognition of pregnancy, embryonic signals, mechanisms of implantation and pregnancy-specific changes in the uterine environment have been extensively studied in domestic livestock and human [9, 10]. There also have been recent, significant advances in metabolic profiling and proteomic analyses using mass spectrometry (MS) to identify circulating or excreted ‘biomarkers’ (small molecules) produced during specific physiological states. These studies are revolutionizing our understanding of biological mechanisms associated with physiological and biomedical events, including early pregnancy in the human [11–17]. For example, biomarker detection has identified 14 indicators reflective of preeclampsia in women that, in turn, have enhanced prediction, diagnosis, monitoring, and patient care [13, 15–17]. Circulating biomarker proteins also have been found indicative of pregnancy in the domestic dog [18], several wild canid species (island fox, *Urocyon littoralis*, fennec fox, *Vulpes zerda*, gray wolf, *Canis lupis*, and Mexican gray wolf, *Canis lupus baileyi*) [19], and the black bear (*Ursus americanus*) [20].

All of these studies to-date have relied on blood serum or plasma analysis. But for biomarkers to be useful for diagnoses or understanding bioprocesses in wild species requires non-invasive tactics to avoid physical or chemical restraint that can disrupt sensitive events, like pregnancy establishment and gestation [21–23]. For this reason, our laboratory has focused on feces as a resource for non-invasively understanding reproductive mechanisms and determining reproductive status, especially via hormone metabolite monitoring. For example, the evaluation of serially collected fecal samples from the cheetah has been useful for characterizing this species’ unique reproductive cycle [4, 24, 25], exploring the influence of exogenous gonadotropins [26], including for timed artificial insemination [8], and for examining the influence of management factors on reproductive success of both females [27, 28] and males [29, 30]. Particularly important is that fecal samples can be collected frequently from a cheetah’s enclosure without causing perceived stress or altering best practice animal care. For these reasons and because there has been exploding interest in biomarkers for predicting physiological and reproductive health [31], it was logical that we examine the hypothesis that systemic protein expression in feces is influenced by the gravid state.

Our target species, the cheetah, was also a natural priority given struggles to improve *ex situ* management. The ability to effectively determine why some mating cheetahs produce cubs and others do not has implications for understanding the nuances of fertility, including complex factors related to physiological versus environmental inadequacies or compromise. For example, there are decades of data published on the uniqueness of the cheetah ejaculate and the male’s routine production of low sperm concentrations and high proportions of sperm malformations [32]. Even with these distinctive features, a single mating can produce pregnancy [7]. Therefore, it is possible that multiple other factors are predominant drivers of pregnancy success, ranging from copulatory frequency/intensity to a subpar *ex situ* environment. However, the priority is to determine if mated females are actually establishing pregnancy, information perhaps to be gleaned from exploring biomarkers in feces. Increased support for a novel examination of the cheetah fecal proteome was derived from a recent finding that protein separation and identification have been useful to distinguishing pregnancy (from failed pregnancy) in another carnivore, the polar bear (*Ursus maritimus*) [33].

Our first objective was to identify via MS what was expected to be an expansive protein profile in fecal material of cheetah females. Secondly, we aimed to isolate proteins from this list that may be playing a role in early pregnancy establishment while verifying detection of these candidates via immunoblotting.

## Materials and methods

### Animals

The study was conducted in strict accordance with recommendations in the Guide for the Care and Use of Laboratory Animals of the National Institutes of Health. All samples required for this project were available in our biorepository and were collected non-invasively (feces), therefore not requiring specific IACUC approval or special permits. Female cheetahs (n = 26) were housed at seven institutions throughout the USA, all of which were accredited by the Association of Zoos & Aquariums. Each cheetah had been born earlier in captivity and managed according to protocols and husbandry guidelines established by the Cheetah Species Survival Plan (SSP), a cooperative, cross-institutional breeding program [6, 34]. The study was conducted in strict accordance with recommendations in the Guide for the Care and Use of Laboratory Animals of the National Institutes of Health. Study animals were adults and 2 to 10 yr of age (mean ± standard error of the mean [SEM], 5.7 ± 0.4 yr). Diet included feeding a commercial meat product (beef or horse-based) at least 5 d/wk with occasional supplements of whole rabbit, bones (beef, horse, bison, venison), and/or organ meat (beef, horse, venison); water was available *ad libitum* [35, 36]. Fecal samples were collected opportunistically from females that were scheduled for natural breeding (on the basis of SSP breeding management recommendations) or to be given exogenous gonadotropins with no subsequent semen deposition (via natural or by artificial insemination). Details on the specifics of the simplistic, exogenous hormone protocols used to stimulate follicle development (with equine chorionic gonadotropin, eCG; Sigma-Aldrich, St. Louis, MO) and then ovulation (with human chorionic gonadotropin [hCG; Sigma-Aldrich] or porcine luteinizing hormone [LH; Sioux Biochemical, Sioux Center, IA]) are found elsewhere [26, 37]. Pregnancy was verified by birth of offspring. A non-pregnant (luteal) phase was confirmed by an increase in fecal progestagen metabolite concentration after gonadotropin (eCG/hCG or eCG/LH) administration [8, 24]. Non-ovulatory (control) episodes for each female were verified by lack of an increase in progestagens during the fecal sampling period.

### Fecal collection and steroid and protein extraction

Freshly-voided feces were collected 3 d/wk for 8 to 13 wk/female post-breeding or gonadotropin injections. Approximately 50 g of each sample were placed in a labeled, plastic bag and stored at -20°C. Fecal samples were lyophilized (VirTis, 35L Ultra Super XL-70, Gardiner, NY), pulverized using a mallet, and stored at -20°C until processed further.

To verify reproductive cyclicity (baseline) or ovulation via a significant progestagen increase above baseline [4], steroid hormone metabolites were extracted from fecal sample aliquots as previously described [38]. MS analysis was also completed on fecal aliquots from samples submitted to steroid hormone extraction and examination. Efficiency of steroid extraction was evaluated by adding radiolabeled hormone (^3^H-progesterone or ^3^H-cortisol; 4,000–8,000 dpm) to each fecal sample prior to boiling extraction. The overall mean (± SEM) extraction efficiency for all samples was 75.7% ± 0.3%. Fecal extracts were diluted 1:20 to 1:10,000 in BSA-free phosphate buffer (2.2 M NaH_2_PO_4_, 3.5 M Na_2_HPO_4_, 0.3 M NaCl, H_2_O; pH, 7.0) for analysis by enzyme immunoassay (EIA). Fecal hormone data were expressed as μg/g dry feces.

Four fecal samples from each animal were pooled for each biological replicate, one sample/wk for 4 consecutive wk. For pregnant or non-pregnant (luteal phase) females, aliquots were taken from resource fecal samples collected during the first 4 wk post-breeding or gonadotropin treatment. To extract total protein, ~0.5 g of frozen, dried feces from each female was suspended in phosphate buffered saline with protease inhibitor (1:1,000), agitated for 30 min, and an ammonium sulfate saturation (60%) used to precipitate proteins. Protein extract solution was filtered to reduce bacterial contamination and desalted using Millipore spin columns (Amicon Ultra-0.5; 3 kDa MWCO). All steps were performed at 4°C. The Bradford assay (Bio-Rad Protein Assay, Hercules, CA) was used to determine sample protein concentration. Differences in sample steroid and protein concentrations among groups were determined using a linear mixed effect model in R package ‘nlme’ (R version 3.1.3) that included sampling date nested within individual as a random effect [39]. Effects were considered significant at *P* < 0.05.

### Fecal estrogen metabolite analysis

Estrogen metabolite concentrations in diluted fecal extracts were determined using an estradiol EIA validated in our laboratory for use in the cheetah [24, 25]. This assay relied on a polyclonal anti-estradiol antibody (R4972; C. Munro, University of California, Davis, CA) that cross-reacted with 17β-estradiol (100%), estrone (3.3%), and < 0.01% with estrone sulfate, progesterone, testosterone, cortisol, and corticosterone. Antibody (0.05 ml) was added to 96-well microtiter plates (Nunc-Immuno, Maxisorp; Fisher Scientific, Waltham, MA) and equilibrated for 12 to 48 h (4°C). Unbound antibody was removed with wash solution (1.5 M NaCl, 0.06 mM, 5.0 ml Tween 20 [Sigma-Aldrich, P1379]), and diluted samples (in duplicate) and standards (in triplicate) (0.02 ml; 97.5–25,000 pg/ml; 17β-estradiol; Sigma Diagnostics) were added along with a peroxidase enzyme-conjugated 17β-estradiol (1:50,000; 0.05 ml; C. Munro). The plate was incubated for 2 h (23°C) before unbound components were removed with wash solution. A chromagen solution (0.1 ml; 2,2'-azino-di-[3-ethyl-benzthiazoline-6-sulphonic acid], ABTS) was added to each well and incubated ~40 min before optical densities were determined using a microplate reader (Dynex MRX, reading filter at 405 nm, reference filter at 540 nm). Sensitivity of the estradiol EIA at maximum binding was 1.95 pg/well. The inter-assay coefficients of variation (CV) for two internal controls were 9.2% (mean binding, 30.2%) and 13.8% (mean binding, 74.6%), and CV for all sample duplicates was <10% (n = 30 assays). The intra-assay CV of two internal controls and a sample pool at multiple points across a microplate was <10%. There was no evidence of matrix interference as addition of diluted fecal extract to synthetic standards did not alter the amount observed (y = 1.28x – 4.03, R^2^ = 0.998, F_1,7_ = 4193.7, *P* < 0.001). Serial dilutions of fecal extract yielded a displacement curve parallel to the standard curve (y = 1.05x – 5.80, R^2^ = 0.978, F_1,7_ = 314.5, *P* < 0.001) indicating that antibody was recognizing the fecal metabolites proportionally to the synthetic standard curve. Estrogen baselines were calculated using an iterative process, excluding values greater than the overall mean plus 1.5 standard deviations [4, 40].

### Fecal progestagen metabolite analysis

Progestagen metabolite concentrations in diluted fecal extracts were determined using a monoclonal antibody assay routinely applied in our laboratory (no. CL42, Quidel Co., San Diego, CA) and the associated horseradish-peroxidase ligand [24, 25]. Plates were prepared as for the estradiol assays, and diluted samples (in duplicate) and standards (in triplicate) (0.05 ml; 156–40,000 pg/ml; Sigma Diagnostics) were added along with a peroxidase enzyme-conjugated progesterone (1:40,000; 0.05 ml; C. Munro). Plates were incubated 15 to 30 min after adding the chromagen solution, and optical densities were determined using a microplate reader (as for estradiol). The inter-assay coefficients of variation (CV) for two internal controls were 12.9% (mean binding, 30.9%) and 14.2% (mean binding, 69.2%), and the CV for all sample duplicates was <10% (n = 24 assays). The intra-assay CV of two internal controls and a sample pool at multiple points across a microplate was <10%. There was no evidence of matrix interference as adding diluted fecal extract to synthetic standards did not alter amount observed (y = 1.17x – 3.15, R^2^ = 0.985, F_1,7_ = 454.8, *P* < 0.001). Serial dilutions of fecal extract yielded a displacement curve parallel to the standard curve (y = 1.14x – 21.25, R^2^ = 0.895, F_1,7_ = 59.9, *P* < 0.001) indicating that antibody was recognizing the fecal metabolites proportionally to the synthetic standard curve.

### Protein digestion and TMT labeling

Protein extracts from the pooled fecal samples were prepared in two separate analyses: 1) protein extracts from five confirmed pregnant cheetahs (with eventual cub births) compared to extracts from those same five individuals in a non-ovulatory, control state; and 2) protein extracts from five pregnant females compared to extracts from different, but age-matched, similarly healthy individuals experiencing a non-pregnant luteal phase. For logistical reasons, it was impossible to have females in the second analysis experience both a pregnancy and a non-pregnant, luteal phase.

Protein labeling and nano-scale, reverse phase chromatography and tandem mass spectrometry (nanoLC-MS/MS) were used to generate protein lists and relative concentrations for pregnant versus non-pregnant sample extracts via a commercial proteomics service provider (Cornell University Proteomics and Mass Spectrometry Facility, Ithaca, NY). Proteins were denatured in a final concentration of 0.1 M phosphate buffer, 8 M urea, and 0.15% sodium dodecyl sulfate (SDS). Protein concentration for each sample was determined by the Bradford assay using bovine serum albumin as the calibrant, and further quantified by a precast NOVEX 10% Bis-Tris mini-gel (Invitrogen, Carlsbad, CA) along with serial amounts of *E*. *coli* lysates (2, 5, 10, 20 μg/lane). SDS gels were visualized with colloidal Coomassie blue stain (Invitrogen) and imaged by a Typhoon 9400 scanner using ImageQuant Software version TL 8.1 (GE Healthcare, Little Chalfont, United Kingdom). Further protein processing was according to the Thermo Scientific’s TMT 10plex Mass Tagging Kits and Reagents protocol with a slight modification; a total of 50 μg protein of each sample was first reduced with 20 mM tris(2-carboxyethyl)phosphine for 1 h (room temperature), alkylated with 20 mM iodoacetamide for 1 h in the dark, and then quenched by addition of 20 mM dithiothreitol (DTT). Alkylated proteins were precipitated by adding 6 volumes of ice-cold acetone and incubating at -20°C overnight, and reconstituted in 50 μl of 100 mM triethylammoniumbicarbonate. Each sample was digested with 5 μg trypsin for 18 h (37°C). The TMT 10-plex labels (dried powder) were reconstituted with 25 μl of anhydrous ACN prior to labeling and added in a 1:2 ratio to each of the tryptic digest samples for labeling over 1 h (room temperature). Peptides from the 10 samples (5 pregnant and 5 non-pregnant) were mixed with each tag, respectively: 126-tag, 127N-tag, 127C-tag, 128N-tag, 128C-tag, 129N-tag, 129C-tag, 130N-tag, 130C-tag, and 131-tag. The same labeling as above was conducted for the second set of 10 samples.

After checking label incorporation using Orbitrap Elite (Thermo-Fisher Scientific, San Jose, CA) by mixing 1 μl aliquots from each sample and desalting with SCX (strong cation-exchange) ziptip (Millipore, Billerica, MA), the 10 samples were pooled, evaporated to dryness, and subjected to cation exchange chromatography using a PolyLC SCX cartridge (PolyLC Inc. Columbia, MD). The SCX cartridge (10 mm id x 14 mm) was Polysulfoethyl Aspartamide with a particle size at 12 μm and 300 Å pore size. The pooled TMT labeled, tryptic peptides were then reconstituted with 3.0 ml of loading buffer (10 mM potassium phosphate, pH 3.0, 25% acetonitrile [ACN]). Sample pH was adjusted to 3.0 with formic acid prior to cartridge separation. After conditioning of the SCX cartridge with loading buffer, each sample (~500 μg) was added and washed with 2.0 ml more of loading buffer. Peptides were eluted in one step by 1.0 ml of loading buffer containing 500 mM KCl. Desalting of SCX fractions was carried out using solid phase extraction (SPE) on Sep-Pak^®^ Cartridges (Waters, Milford, MA) and the eluted tryptic peptides evaporated to dryness for the first dimensional LC fractionation via a high pH, reverse phase chromatography (as described below).

### High pH reverse phase (hpRP) fractionation

The hpRP chromatography was carried out using a Dionex UltiMate 3000 HPLC system with the built-in micro-fraction collection option in its autosampler and UV detection (Sunnyvale, CA) as reported previously [41]. The TMT 10-plex tagged tryptic peptides were reconstituted in buffer A (20 mM ammonium formate, pH 9.5 in water) and loaded onto an XTerra MS C18 column (3.5 μm, 2.1x150 mm; Waters, Milford, MA) with 20 mM ammonium formate (NH_4_FA), pH 9.5 as buffer A and 80% ACN/20% 20 mM NH_4_FA as buffer B. Liquid chromatography (LC) was performed using a gradient from 10 to 45% of buffer B in 30 min (flow rate 200 μl/min). Forty-eight fractions were collected at 1 min intervals and pooled into a total of 18 fractions based on UV absorbance at 214 nm and with a multiple fraction concatenation strategy [42]. The latter is a retention time multiplexing approach through pooling disparate first dimensional hpRP fractions prior to the second dimension nanoLC-MS/MS analysis. Under these conditions, there is no appreciable degradation in chromatographic resolution or reduction in peptide identifications compared to individually analyzed fractions [42]. All fractions were dried and reconstituted in 100 μl of 2% ACN/0.5% formic acid (FA) for nanoLC-MS/MS analysis.

### Nano-scale reverse phase chromatography and tandem mass spectrometry (nanoLC-MS/MS)

The nanoLC-MS/MS analysis was carried out using an Orbitrap Elite (Thermo-Fisher Scientific) mass spectrometer equipped with a nano ion source using high-energy collision dissociation (HCD). The Orbitrap was fitted with a CorConneX nano ion source (CorSolutions LLC, Ithaca, NY) coupled with the UltiMate3000 RSLCnano (Dionex). Each reconstituted fraction (5 μl) was injected onto a PepMap 100 C-18 RP nano trap column (5 μm, 100 μm x20 mm, Thermo) with nanoViper Fittings at 20 μl/min flow rate for on-line desalting. Fractions then were separated on a PepMap C-18 RP nano column (3 μm, 75 μm x 25 cm) and eluted in a 120 min gradient of 5 to 35% ACN in 0.1% FA at 300 nl/min. This was followed by a 7 min ramping to 95% ACN-0.1% FA and a 7 min hold at 95% ACN-0.1% FA. The column was re-equilibrated with 2% ACN-0.1% FA for 28 min prior to the next run. Eluted peptides were detected by Orbitrap through a ‘Plug and Play’ nano ion source housing a 10 μm analyte emitter (NewObjective, Woburn, MA). The Orbitrap was operated in the positive ion mode with nano spray voltage set at 1.6 kV and source temperature at 250°C. External calibration for the Fourier transform (FT) mass analyzer was performed. This instrument was operated in data-dependent acquisition (DDA) mode using the FT mass analyzer for one survey MS scan for selecting the top 15 precursor ions. This process was followed by data-dependent HCD-MS/MS scans on the precursor peptides with multiple charged ions above a threshold ion count of 8,000 with a normalized collision energy of 37.5%. MS survey scans were set at a resolution of 60,000 (fwhm at *m*/*z* 400) for the mass range of m/z 375 to 1,800 with AGC = 1e6 and Max IT of 100 ms. MS/MS scans were set at 30,000 resolutions with a 1.5 amu of isolation width with AGC = 1e5 and Max IT of 250 ms for the mass range m/z 100 to 2,000. Dynamic exclusion parameters were set at a repeat count 1 with a 30 s repeat duration, an exclusion list size of 500, and a 60 s exclusion duration with ±10 *ppm* exclusion mass width. Activation time was 0.1 ms for HCD analysis. All data were acquired using Xcalibur 2.2 operation software (Thermo-Fisher Scientific).

### Data processing, protein identification, and data analysis

All MS and MS/MS raw spectra from each set of TMT10-plex experiments were processed using the Proteome Discoverer 1.4 (PD1.4, Thermo). Spectra from each DDA file were output as an MGF file for a subsequent database search using in-house licensed Mascot Daemon (version 2.5.1, Matrix Science, Boston, MA). The Cat RefSeq database containing 33,288 sequence entries was downloaded on March 20, 2015 from NCBInr and used for database searches. Default search settings used for 10-plex TMT quantitative processing and protein identification in Mascot server were: two mis-cleavage for full trypsin with fixed carbamidomethyl modification of cysteine, fixed 10-plex TMT modifications on lysine and N-terminal amines, and variable modifications of methionine oxidation and deamidation on asparagines/glutamine residues. Peptide mass tolerance and fragment mass tolerance values were 10 ppm and 20 mmu, respectively. To estimate the false discovery rate (FDR) for a measure of identification certainty in each replicate set, an automatic decoy database search was performed in Mascot by choosing the decoy checkbox in which a random sequence of database was generated and tested for raw spectra along with the real database. The significant scores at a 95% confidence interval for the peptides defined by a Mascot probability analysis greater than ‘identity’ along with ~0.5% FDR, and ions score cutoff >20 were used as filters. FDRs of MS analyses of pregnant to non-ovulatory control samples and pregnant to non-pregnant luteal phase samples were 4.7% and 6.7%, respectively. Proteins identified in all 10 TMT channels that contained at least two peptides were further analyzed. Intensities of the reporter ions from TMT 10-tags upon fragmentation were used for quantification, and the relative quantitation ratios were normalized to median ratio for the 10-plex in each set of experiments. Comparison between paired samples in the two groups was conducted using Microsoft Excel software.

### Isolation of candidate pregnancy biomarkers

Candidate biomarkers of pregnancy were selected by subjecting each of the two sets of analyses (pregnant versus non-pregnant luteal and pregnant versus non-ovulatory control) to a series of elimination steps, then comparing remaining proteins in each list to each other to find commonalities. For the first elimination step and to secure relative protein quantitation ratios, the candidate protein had to be comprised of at least two unique peptides that produced a complete TMT reporter ion series; those failing to meet this criterion were eliminated from further consideration. Next, *P*-values for each protein ratio were considered for each set of samples (pregnant versus non-pregnant) in each of the two analyses. Identified proteins with no significant difference (*P* > 0.05) in relative quantification between reproductive groups were deleted from pregnancy biomarker consideration. Remaining proteins were then cross-checked to ensure that existing significance was consistent among all sample sets such that the protein in question was either consistently over- or under-expressed in pregnant versus non-pregnant samples. The final remaining protein lists were compared between the two analyses to identify common proteins, with these then designated as candidate biomarkers of pregnancy.

### Verification of selected biomarker candidates via western blotting analysis

Identified protein candidates were verified for ability to be detected in cheetah fecal samples and relative value for determining pregnancy using commercially available antibodies and western blotting analysis. Total proteins in fecal extracts were separated by SDS-PAGE, transferred to a PVDF membrane, blocked with 5% milk, and incubated overnight at 4°C with primary antibody at a dilution determined by optimization (Table 1). Membranes were then incubated with a 1:2,500 dilution of antisera of the appropriate species IgG conjugated with horseradish peroxidase. Proteins were visualized on a G:Box Chemi XRQ (Syngene) and images analyzed using GeneTools software version 4.03 (Syngene). The positive control varied based on the antibody tested (Table 1). Coomassie staining and subsequent imaging and analysis of total protein in each lane were completed to serve as a loading control across samples [43]. Samples to be compared were run on the same blot where possible to avoid inter-blot variation. Differences in protein intensity among reproductive groups were determined using a linear mixed effect model in R package ‘nlme’ (R version 3.1.3) that included sampling date nested within individual as a random effect. Effects were considered significant at *P* < 0.05 [39].

**Table 1:** Specific information for antibodies used in western blotting of fecal proteins from pregnant and non-pregnant cheetahs.

**Protein**	**Manufacterer (catalog number)**	**Primary dilution**	**Positive control**	**Specific band size(s) (kDa)**
Alkaline phosphatase	Abcam (ab72629)	1:1,000	Small intestine lysate (mouse)	57
Complement C3	Abcam (ab14232)	1:1,000	Pooled serum (cheetah)	40, 68
Immunoglobulin J chain	Aviva Systems Biology (ARP55440_P050)	1:1,000	Ig J chain 293T lysate (mouse)	18
Myosin-binding protein C	Abcam (ab124196)	1:1,000	Skeletal muscle lysate (human)	37
Trefoil factor 3	Abcam (ab101099)	1:1,000	Colon tissue lysate (human)	10

## Results

### Influence of reproductive status on fecal estrogen, progestagen, and total protein concentrations

**Fig 1:**
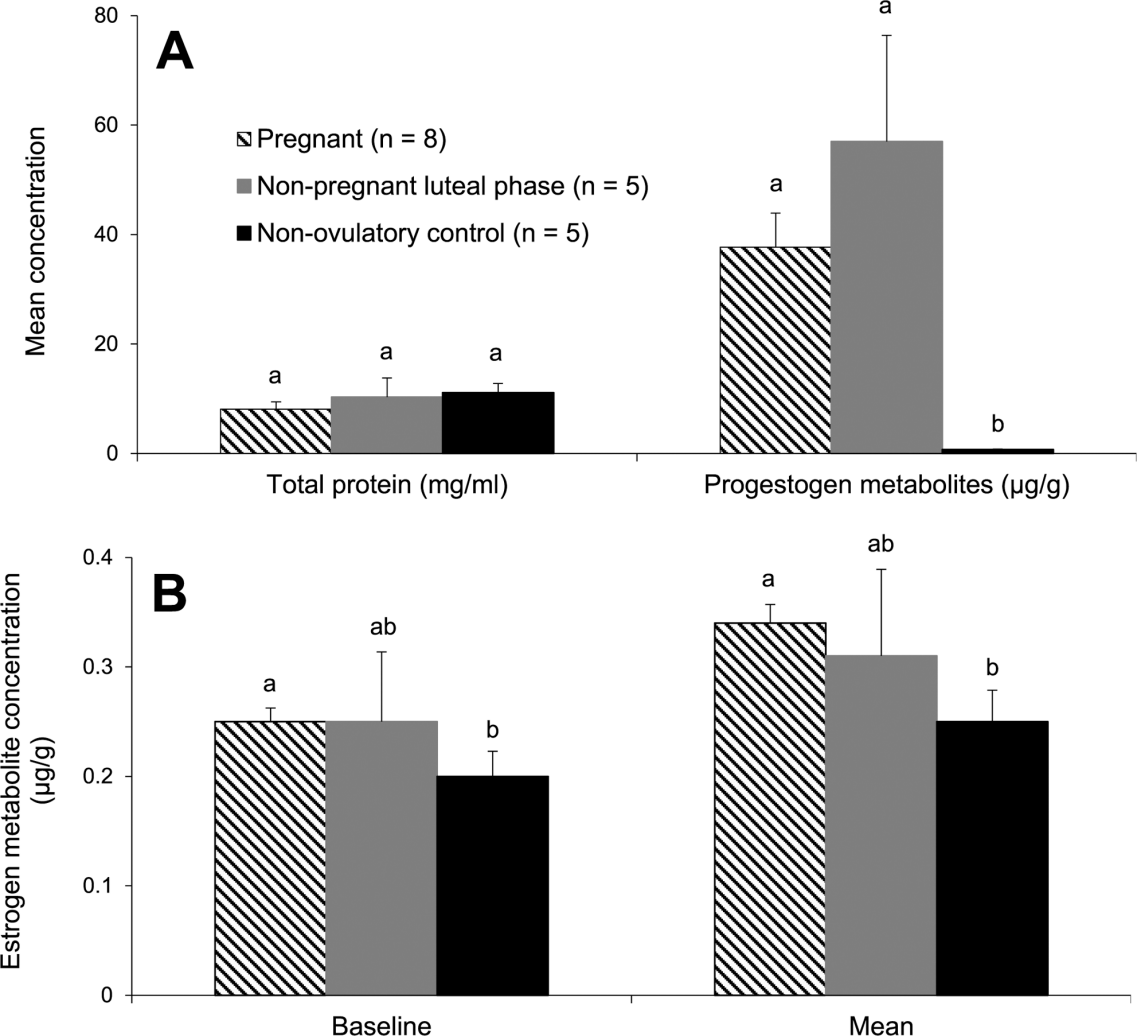
Mean (± SEM) total protein and steroid hormone metabolite concentrations of fecal extracts from female cheetahs. Protein was extracted from pooled samples collected over 28 d and steroid hormone metabolites extracted from individual fecal samples collected over 8 to 13 wk. Bars with different letters within a metric were different (*P* < 0.05).

**Fig 2:**
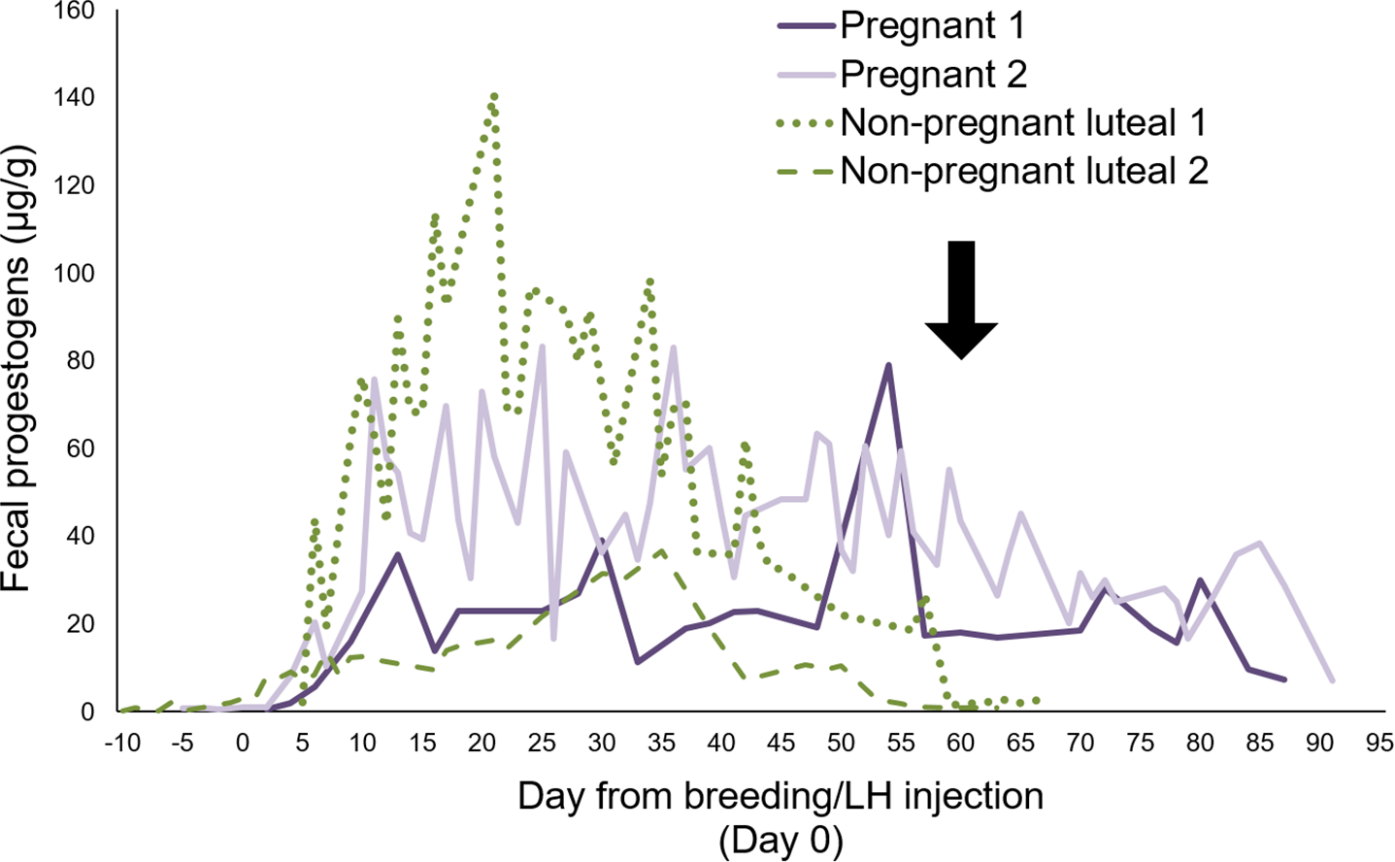
Representative fecal progestogen metabolite profiles of four cheetahs that were pregnant or experiencing a non-pregnant luteal phase (after gonadotropin treatment with LH/hCG to stimulate ovulation). Arrow indicates approximate termination of elevated progestogens associated with a non-pregnant luteal phase.

Mean total protein extracted from samples pooled over 28 d was not different (*P* > 0.05) among reproductive groups (Fig 1A). Fecal estrogen and progestagen metabolite concentrations were found to validate our assignments of specific females to various reproductive groups for mass spectrometry and then western blot analysis. Fecal estrogen metabolite concentrations were higher (*P* < 0.01) in pregnant than non-ovulatory females (Fig 1B), but were not different (*P* > 0.05) between non-pregnant luteal phase females and either other group. Although mean progestagen concentrations were expectedly lower (*P* < 0.03) in non-ovulatory females, comparing sample values from pregnant versus non-pregnant luteal phase females revealed no difference (*P* > 0.05; Fig 1A). Complete protein and steroid hormone data by reproductive group, female, and date are included in supplemental information S1 Table and S1 Appendix. When excreted progestogen patterns were compared among treatment groups, we found variable, but consistently elevated concentrations of this metabolite for 8 to 12 wk. However, fecal progestogen profiles were clearly differentiated between non-pregnant and pregnant cheetahs at ~60 d of monitoring, with gestating females producing extended excretion patterns, usually until the time of parturition (representative females depicted in Fig 2).

### Influence of reproductive status on protein expression

**Fig 3:**
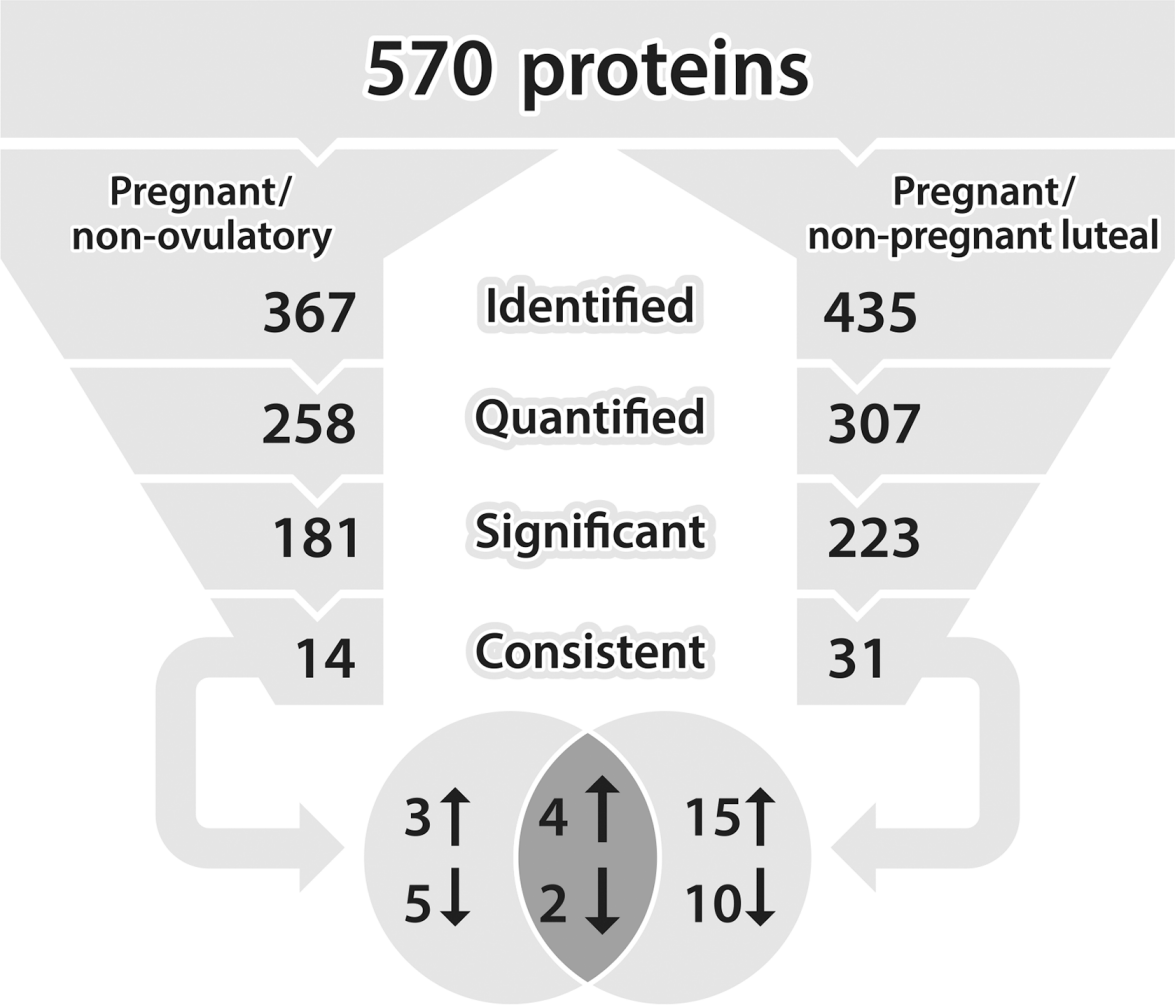
Workflow used to reduce total protein output lists from cheetah fecal samples analyzed with mass spectrometry in two sets: 1) pregnant (n = 5) paired with non-ovulatory (control) samples (n = 5) from the same females; and 2) pregnant (n = 5) paired with matched samples from other non-pregnant females experiencing a luteal phase (n = 5). Samples were labeled with reporter ions for relative quantification of each protein between pregnant and non-pregnant reproductive groups. From all ‘identified’ proteins in both lists, those proteins failing to produce at least two unique peptides for relative quantitation were deleted from further consideration, leaving only those that could be ‘quantified’. After comparing individual protein quantifications between the five pregnant and non-pregnant paired samples in each analysis, lists were further reduced to include proteins with expression differences that were significant. Finally, only those proteins were retained that were consistent in over- or under-expression in all pregnant versus non-pregnant sample comparisons. Black arrows indicate increased or decreased protein expression in pregnant versus non-pregnant (i.e., non-ovulatory or non-pregnant luteal) samples.

The two MS evaluations yielded 570 proteins from the 20 analyzed fecal extracts (Fig 3). Of the 10 extracts assessed for the pregnant and non-ovulatory groups, 367 proteins were revealed (Fig 3). The same evaluation of 10 extracts from the pregnant and non-pregnant luteal phase groups identified 435 proteins (Fig 3). Complete protein lists from both nanoLC-MS/MS analyses are presented in supplemental information S2 and S3 Tables. Of all relatively quantified proteins, ~200 in each comparison demonstrated different (*P* < 0.05) expression between pregnant and non-pregnant samples (Fig 3). Fourteen proteins were consistently either over- or under-expressed in all pregnant versus non-ovulatory sample comparisons, and 31 were consistently different in all pregnant versus non-pregnant luteal phase sample comparisons (Fig 3). Eight proteins (3 over- and 5 under-expressed) in the pregnant versus non-ovulatory comparison and 25 (15 over- and 10 under-expressed) in the pregnant versus non-pregnant luteal phase comparison were unique. Of these, four were more abundant and two less abundant in both analyses of pregnant compared to non-pregnant individuals (i.e., a combination of the non-pregnant luteal phase and non-ovulatory samples) (Fig 3).

Isolation of pregnancy biomarker candidates yielded four proteins that were over-expressed in pregnant compared to non-pregnant samples: immunoglobulin J chain, trefoil factor 3, nebulin, and complement C3 (Table 2, above dotted line). This analysis yielded two proteins that were under-expressed in pregnant samples: alkaline phosphatase and myosin-binding protein C (Table 2, below dotted line). As revealed in Table 2, protein expression differed as much as 3-fold between the pregnant cheetahs and either their luteal phase or non-ovulatory control counterparts. Protein immunoglobulin J chain demonstrated the most uniform expression change, with 70% of sample comparisons different (*P* < 0.05) in abundance between pregnant and non-pregnant groups (Table 2). Complete protein abundance ratios for six pregnancy biomarker candidates are available in supplemental information S4 Table.

**Table 2:** Protein identification and relative quantification ratios (± SEM) of pregnancy biomarker candidates (n = 6) from cheetah fecal extracts in two mass spectrometry analyses.

**Protein (***Felis catus***)**	**NCBI accession no.**	**MW (kDa)**	**Biological process**	**Mean P**^**a**^**/L**^**b**^ **ratio**	**Mean P**^**a**^**/N**^**c**^ **ratio**	**Samples with significant expression change**^**d**^
Immunoglobulin J chain	gi|410957470	18	Adaptive immune response	2.45 ± 0.6	1.92 ± 0.4	70%
Trefoil factor 3	gi|194353917	9	Defense response, mucosal	3.41 ± 1.0	1.88 ± 0.5	50%
Nebulin	gi|755758826	500	Muscle filament sliding	2.87 ± 0.9	1.52 ± 0.3	50%
Complement C3	gi|755695108	187	Complement activation	1.31 ± 0.1	1.50 ± 0.6	40%
Alkaline phosphatase	gi|586998392	60	Dephosphorylation	0.93 ± 0.2	0.88 ± 0.1	30%
Myosin-binding protein C	gi|755751065	69	Cell adhesion	0.41 ± 0.1	0.93 ± 0.2	50%

^a^P = pregnant (n = 5).

^b^L = non-pregnant luteal phase (n = 5); ovulation induced with exogenous gonadotropins, and no sperm deposited.

^c^N = non-ovulatory control (n = 5).

^d^Percentage of analyzed samples that yielded significant (*P* < 0.05) expression change between pregnant and non-pregnant groups (n = 10 total).

### Pregnancy prediction capability of selected protein biomarker candidates

**Fig 4:**
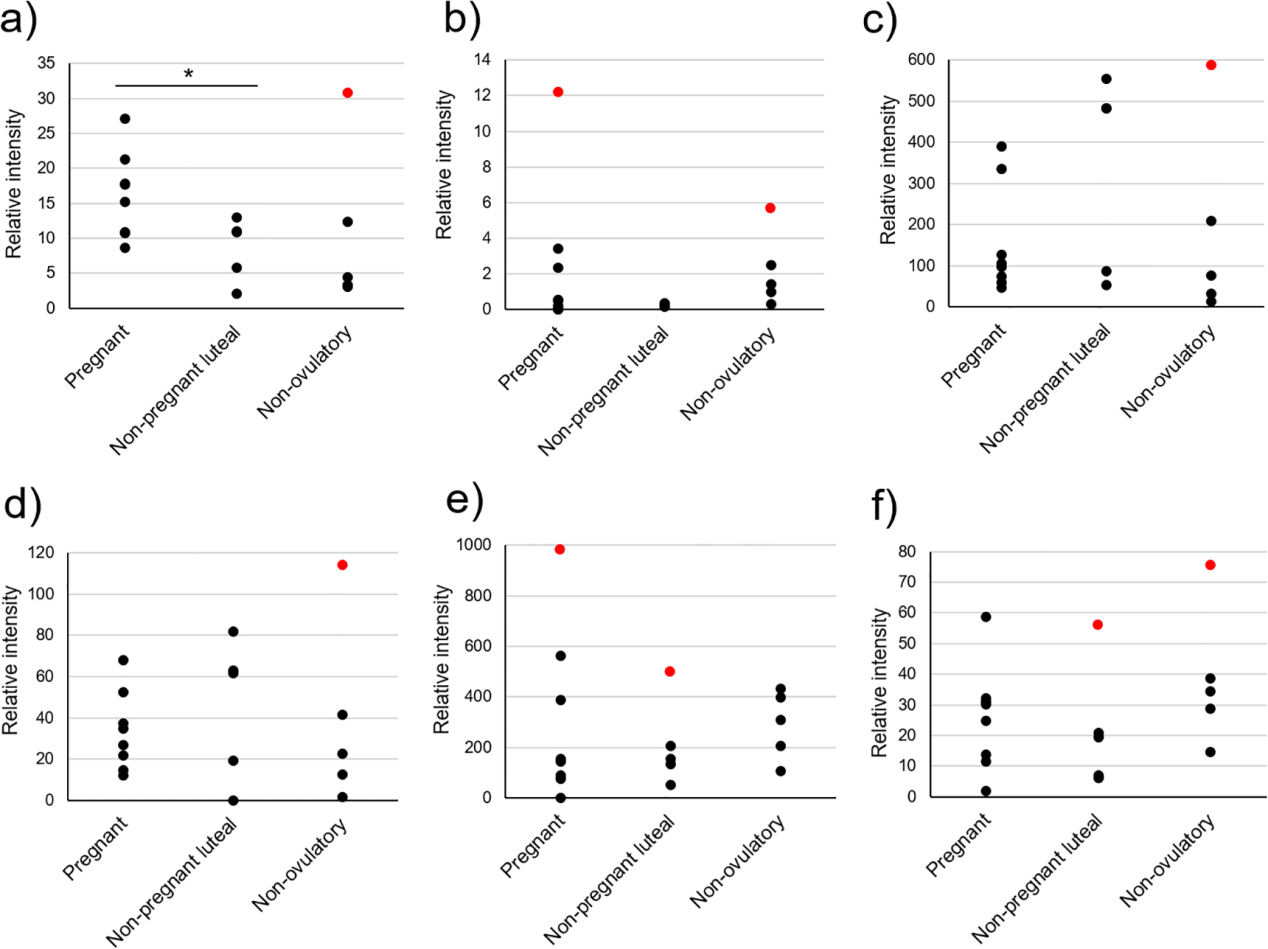
**Western blot intensities for protein candidates: a) immunoglobulin J chain; b) trefoil factor 3; c) complement C3 (iC3b fragment); d) complement C3 (C3dg fragment); e) alkaline phosphatase; and f) myosin binding protein C.** Source was fecal extracts from female cheetahs during the first 4 wk of pregnancy (n = 8), a non-pregnant luteal phase (n = 5), or non-ovulatory controls (n = 5). Red dots indicate outliers (i.e., >1.5 interquartile ranges above the third quartile or below the first quartile). Asterisk indicates difference trend (*P* < 0.1).

**Fig 5:**
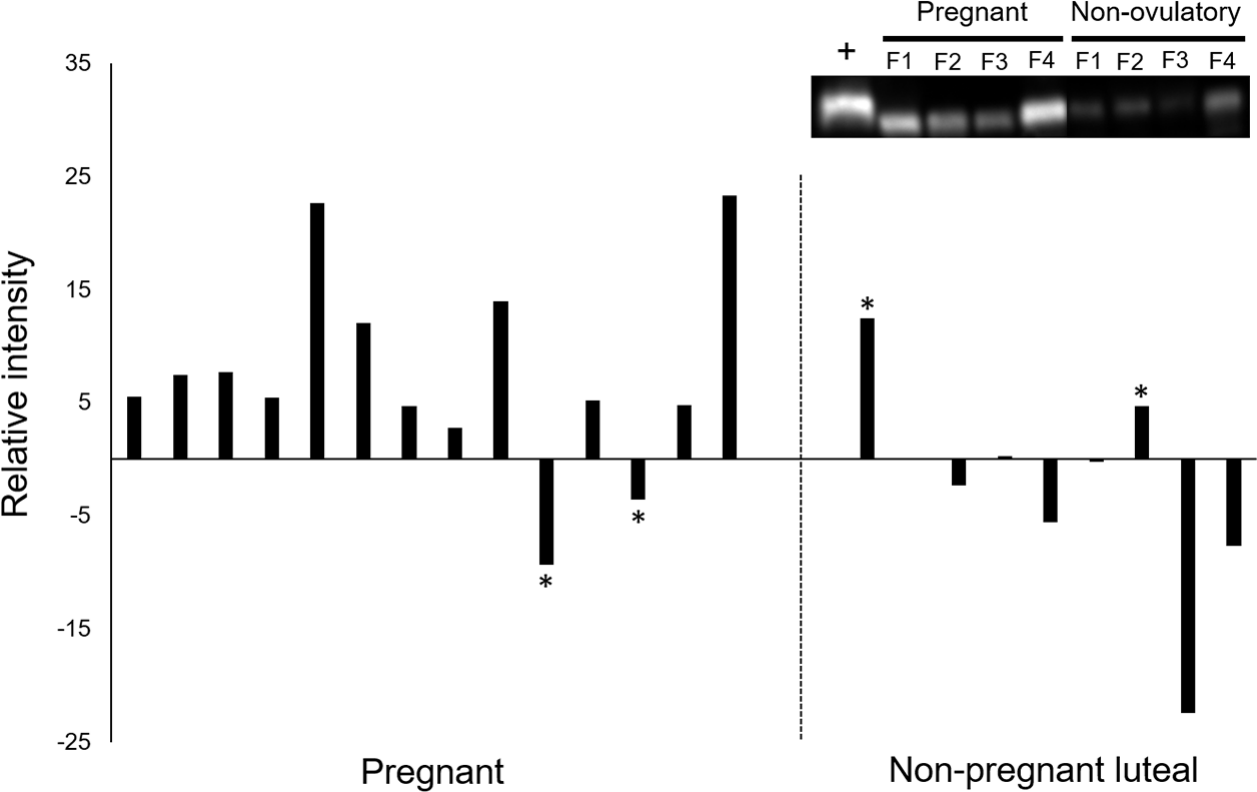
Intensity of immunoglobulin J chain expression for cheetahs that were pregnant (n = 14) or in a non-pregnant luteal phase (n = 9) compared to samples from the same females during a non-ovulatory control period. Each bar represents a single female. Inset representative blot image of samples from the same four pregnant females during a non-ovulatory period. Asterisks indicate instances when immunoglobulin J chain expression did not change between ovulatory and non-ovulatory states to allow accurate pregnancy determination.

Commercially available antibodies allowed successful detection of five of our six protein biomarker candidates in cheetah fecal extracts. An available antibody to reliably detect the protein nebulin in cheetah feces could not be verified (despite testing multiple antibody sources). Relative intensities of trefoil factor 3 were the lowest of all biomarker candidates at less than half the intensity of the nearest neighboring protein (Fig 4). Immunoblotting for complement C3 yielded two specific protein bands of sizes that corresponded to two known complement C3 fragments, iC3b (68 kDa) and C3dg (40 kDa); however, the full length protein was not detected. Relative intensities of biomarker candidates were widely variable across females for some reproductive groups (Fig 4). Biomarker candidate immunoglobulin J chain (IGJ) expression tended (*P* < 0.1) to be increased during the first 4 wk of pregnancy compared to non-ovulatory counterparts (Fig 4A). Post-hoc analysis after deletion of a maximum of one outlier value per group (Fig 4) revealed higher IGJ expression in pregnant compared to either the non-pregnant luteal phase (*P* < 0.03) or non-ovulatory (*P* < 0.02) controls. There was no difference (*P* > 0.05) in expression of any other analyzed protein after outlier removal. Comparison of known pregnant (n = 14) and non-pregnant luteal phase (n = 9) females to their own, non-ovulatory control samples yielded IGJ expression that was (1) at least one- to two-fold higher in all but two pregnant females and (2) not different or lower in all but two non-pregnant luteal phase individuals (Fig 5 and inset; see S2 Appendix for full blot image). In total, detectable protein expression of IGJ accurately determined pregnancy in 19 of 23 females (82.6%).

## Discussion

The cheetah is one of the world’s most investigated wildlife species. Scientists have intensively studied the biology of this species *ex situ* and *in situ* for almost 4 decades. Our laboratory has focused on understanding the reproductive physiology of this species in areas related to: ovarian activity (morphologically [8, 26] and endocrinologically [4, 25, 44, 45]); sensitivity to exogenous gonadotropins [8, 26, 37]; oocyte development and fertilization *in vitro* [46]; understanding the phenomenon of teratospermia [47] and its relatedness to genetic homozygosity [48]; sperm cryo-sensitivity [35, 49, 50]; and demonstrating the value of artificial insemination with fresh or thawed spermatozoa to produce offspring for enhanced management [8, 26]. Even with this significant amount of fundamental and applied knowledge, it remains difficult to breed cheetahs consistently in zoological environments. For example, only ~20% of individuals in the North American zoo-managed population have ever reproduced [6]. While part of this problem can be attributed to the captive environment itself and suboptimal husbandry, there is a mystery about females that mate with fertile males, but fail to deliver cubs. This issue is compounded by a paucity of information concerning intra-uterine/intra-oviductal events occurring between copulation and a few weeks before parturition. Here, we did the first study of the fecal proteome of the female cheetah at various reproductive states for the purpose of identifying proteins likely involved in early pregnancy establishment. One protein in particular, immunoglobulin J chain (IGJ), was readily detectable and differentially expressed early in pregnancy compared to a non-pregnant state. While laying a foundation for understanding protein-associated mechanisms in maternal pregnancy recognition and gestational maintenance, our findings also can be applied to developing a cheetah (and perhaps carnivore-wide) pregnancy diagnostic tool to improve *ex situ* management.

‘Shotgun proteomics’ through MS analysis has been instrumental in discovery of protein candidates with diagnostic potential [51]. With those efforts as incentive and using two separate MS analyses and the domestic cat RefSeq database (downloaded from NCBInr), we established that there were 570 proteins making up the cheetah fecal proteome. Our findings were within the range of 300 to 900 proteins in human [52] and mouse [53] fecal material as identified using analogous methods. Authors of these publications found variation in the fecal proteome among individuals, akin to our results of differences among cheetahs, as well as those among individual polar bears noted by Curry and colleagues [33]. Such findings emphasize the importance of evaluating multiple animals in a given species to overcome variance among individuals. Additionally, it was useful to calculate protein quantification ratios (Table 2) that facilitated distinguishing the proteome between certain reproductive states, for example, especially pregnancy versus control conditions. Furthermore, it was prudent to minimize the potential impact of daily variations in diet by analyzing pooled fecal samples from each individual, a technique also employed in recent polar bear fecal proteomic work [33]. In the latter study, there were seven protein candidates [33] over-expressed in the feces of pregnant animals, five of which were present in the cheetah fecal proteome. However, none of these five were consistently differentially expressed across all sample comparisons, probably because the polar bear study and our cheetah investigation used different protein separation and quantification techniques [51]. It was noteworthy, however, that three of the proteins making the short list as promising pregnancy biomarkers for both the polar bear and cheetah were functionally related to the immune system. Therefore, it appears that more detailed future examinations focus on proteins associated with the maternal immune system that undergoes significant alterations during gestation, including the early phases of establishing pregnancy [54, 55].

Although most studies to-date (usually in humans) have identified fecal proteins as biomarkers for gastrointestinal health [56–58], here we have shown the value of feces as a source material indicative of reproductive state, specifically early pregnancy. This is an extension of substantial evidence presented over the last 2 decades demonstrating how sequential measurement of steroidal metabolites found in feces is useful for tracking endocrine (usually gonadal and adrenal) status in an array of mammalian species [2, 45, 59]. Steroid hormone metabolite monitoring in voided feces has been widely utilized in the cheetah for understanding basic reproductive cycle characteristics [4], control of ovarian function for assisted reproductive technologies [25], and the impact of the *ex situ* environment on reproductive potential [27, 29] and adrenal function [60, 61]. Expanding discovery to include fecal protein data is a logical next step to addressing persisting physiological gaps in knowledge. Previous work presumed that it would be possible to address the issue of failed pregnancy establishment in mated female cheetahs through non-invasive monitoring of fecal progestogen concentrations and patterns [4, 62]. This approach has been only partially informative, and not until the last trimester of gestation when progestogen level remains elevated in the pregnant cohort [4]. Urinary relaxin, a protein hormone of ovarian origin and predictive of pregnancy in certain mammals, including two felids (e.g., domestic cat [63, 64], Iberian lynx, *Lynx pardinus* [65]), has presented only a weak signal after 50 days of gestation in the cheetah [64]. Therefore, the present study was important in providing the first evidence that there may be one or more early protein signals for pregnancy and detectable in feces. Western blotting verified the ability of our protein candidates to be readily detected with commercial antibodies, thus suggesting the potential of eventually developing a cost-effective, benchtop pregnancy assay for this rare species. We also relied upon the common practice of normalization by fixed point (i.e., positive controls) to ensure comparable quantitation between the western blot experiments. Although conventional, this strategy is known to increase variability among biological replicates, therefore, making it challenging to statistically detect conditions yielding differing results [66]. Indeed, as illustrated in Fig 4, we discovered outlier individuals within our comparison groups, which likely prevented us reaching statistical significance in some cases, including for protein IGJ (Fig 4A). Interestingly, the outlying points within the non-ovulatory group for each of the immune/defense-related proteins are derived from the same individual (Fig 4A–4D). This suggests that the immune system of this cheetah may have been experiencing an unknown challenge at the time of sampling that artificially inflated expression of key immune-related proteins. Post-hoc analysis that excluded these outliers revealed more robust differences and exclusively for the biomarker IGJ.

Among the six identified protein candidates after MS analysis and testing of commercial antibodies, it was the IGJ that presented consistently increased abundance in samples from pregnant compared to non-pregnant cheetahs. When immunoblotting was conducted on samples from pregnant females compared to these same individuals during self, non-ovulatory control periods, IGJ abundance was so robust that this marker was capable of accurately determining pregnancy in >80% of assessed cases. It is known that functionally, IGJ links IgA and IgM to their dimeric and pentameric forms, respectively, and to bind these configurations to their secretory components to allow exocrine immunoglobulin transfer [67]. Due to this role, IGJ as well as IgA, are vital to the secretory immune system. During gestation in the human, there appears to be separate maternal and fetal secretory immune systems that form the barrier between mother and fetus during the first trimester [68–70]. There also is a role in activity of mucosal membranes throughout the body [70], including IGJ production enhanced in human uterine endometrium during the luteal phase [71] and within cervical mucus during pregnancy [72]. Based on these earlier findings, it is possible that we are measuring excreted IGJ as a result of one or both of these mechanisms. One is an activated secretory immune system during formation of the maternal-fetal barrier immediately post-implantation. The other is increased production from mucus-generating cells in the reproductive and/or gastrointestinal tract in response to pregnancy. Both possibilities deserve further research attention.

The mucus producing theory may be less plausible given our findings in attempting to measure trefoil factor 3 (TF3), a group of small peptides that are present in tissues lining the gastrointestinal tract of humans [73]. The trefoil factor family is known to have functions that include interactions with the immune system as well as conspicuous increases in circulation during early to mid-pregnancy in women [74]. Of course, because we were studying intractable, wild cheetahs, it was not possible to collect peripheral blood samples without imposing a potential stress on pregnancy. Meanwhile, our western blots were ineffective in detecting a predictive trend in TF3 abundance in fecal samples of pregnant versus non-pregnant individuals. In fact, this protein was in such low abundance throughout our sample cohort that it proved difficult to even detect regardless of the commercial antibody tested. Therefore, it may well be that markers that may be informative for one species are not in another. More likely, certain valuable biomarker proteins (like peptides in the TF3 family) will be deconstructed during digestion and simply not detectable in voided feces.

Mass spectrometry identified four other protein candidates, nebulin, intestinal alkaline phosphatase, myosin binding protein C, and complement C3 that were non-verifiable as pregnancy biomarkers using commercial antibodies and western blotting. We also were unable to identify potential mechanistic linkages in the scientific literature between expression of nebulin, intestinal alkaline phosphatase, or myosin binding protein C to reproductive state, including pregnancy. None of the commercial antibodies tested recognized nebulin in cheetah feces. This perhaps was due to this immense-sized protein (500 kDa) being fragmented during excretion into peptides that are identifiable by MS, but too small or indistinctive to be recognized by heterologous antibodies. Similarly, rather than detecting full length complement C3 in our samples via western blotting, we observed two distinguishing, predictive bands for the C3 fragments, iC3b and C3dg. Complement C3 and the associated complement system have been reported to increase during healthy pregnancies in women [75], and C3 is detectable as it increases in secretions into the uterine lumen during certain stages of pregnancy in the human [76] as well as mouse [77]. Although identified by MS as increased in certain pregnant cheetahs, it was possible that complement C3 was only being detected as a few specific fragments that varied in abundance from all the peptides labeled as C3. For this reason, this protein did not present as a likely candidate for future pregnancy related studies in the cheetah, especially as we pursue further fecal evaluations.

In summary, this is the first report on detection and abundance of proteins in voided feces of cheetahs managed *ex situ*. This is an initial step to understanding the frequent occurrence of failed parturition in the presence of observed copulations between prime, breeding age adults. The conservation breeding community also is keen to have an early pregnancy diagnostic tool. Importantly, we discovered a novel biomarker of early pregnancy, IGJ, with increased expression distinguishing pregnant from non-pregnant cheetahs within the first 4 wk after mating. From a basic research perspective, the temporal tracking of IGJ concentration throughout gestation now will allow testing the hypothesis that copulations are resulting in fertilization and implantation, but embryonic mortality or fetal resorption are occurring and over a specific timeline. Alternatively, the null hypothesis is that absence of IGJ detection in copulating cheetahs will infer compromises in gamete interaction and placentation. Either cause will be informative in plotting more detailed studies on the impact of universally observed teratospermia [32, 78] or the influence of *ex situ*/environmental conditions [27, 29] on reproductive success. We are also excited about using these findings to advance a cheetah (or carnivore-wide) pregnancy diagnostic tool to improve *ex situ* management. Rapid pregnancy detection means better resource preparation and mobilization to ensure best care of females gestating litters. Likewise, identification of non-pregnant females soon after an unsuccessful mating allows returning these individuals quickly to the breeding population or for re-assignment to infertility treatments. Finally, a predictive assay also may well have relevance under field conditions where females are being monitored closely by radiotelemetry [79]. In such cases, fresh fecal samples could be recovered and evaluated to determine incidence of pregnancy success or failure in nature, thereby giving new insights into the status of this iconic species that remains under ever-increasing and dire threats [1].

## Supporting information

S1 Table Total protein and mean (±SEM) steroid hormone metabolite concentrations of fecal extracts from individual female cheetahs from three reproductive groups: pregnant (P), non-pregnant luteal phase (L), and non-ovulatory control (N). Total protein was determined from analysis of extracts of pooled fecal samples collected over 28 d and steroid hormone metabolite concentrations were determined from extracts of individual fecal samples collected over 8 to 13 wk.
(DOCX)

S2 Table Complete protein identification output (n = 367) of nanoLC-MS/MS analysis of cheetah fecal samples from females in both a pregnant (n = 5) and non-ovulatory control (n = 5) state. (DOCX)

S3 Table Complete protein identification output (n = 435) of nanoLC-MS/MS analysis of cheetah fecal samples from pregnant (n = 5) and non-pregnant luteal phase (n = 5) females. (DOCX)

S4 Table Protein quantitation ratios for identified pregnancy biomarker candidates (n = 6) for each set of individual female cheetah fecal samples that were labeled with TMT reporter ions and compared after nano-scale, reverse phase chromatography and tandem mass spectrometry. Comparisons were made between samples from pregnant and non-pregnant (i.e., non-pregnant luteal phase and non-ovulatory) individuals.
(DOCX)

S1 Appendix Steroid hormone metabolite concentrations (μg/g dry feces) of each fecal sample extracted for individual female cheetahs by date in three reproductive groups: pregnant, non-pregnant luteal phase (luteal), and non-ovulatory control (control). (XLSX)

S2 Appendix Western blot raw images. Images utilized to generate protein intensity quantification. Each set of images represents chemiluminescence of specific antibody binding (left side) and corresponding light image (right side) to visualize molecular size markers (MW) and Coomassie stain of total lane protein as the loading control. Location of positive control (+), pregnant (P), non-pregnant luteal phase (NP), and non-ovulatory control (NO) samples are indicated at the top of each image. Red outline indicates blot sections shown in inset of Fig 5.
(PDF)
