# Supplementary material for: Non-invasive identification of protein biomarkers for early pregnancy diagnosis in the cheetah (Acinonyx jubatus)
Source: PLoS One. 2017 Dec 13;12(12):e0188575. doi: 10.1371/journal.pone.0188575 (PMC5728495; doi:10.1371/journal.pone.0188575)
Supplement: S1 Table — Total protein was determined from analysis of extracts of pooled fecal samples collected over 28 d and steroid hormone metabolite concentrations were determined from extracts of individual fecal samples collected over 8 to 13 wk. (DOCX) [file pone.0188575.s001.docx]

**S1 Table.**

| Reproductive group.Individual code | Total fecal protein (mg/ml) | Baseline estrogen metabolites (µg/g) | Mean (± SEM) estrogen metabolites (µg/g) | Mean (±SEM) progestogen metabolites (µg/g) |
| --- | --- | --- | --- | --- |
| P^a^.MT | 6.60 | 0.21 | 0.30 ± 0.02 | 24.80 ± 2.86 |
| P.AL | 9.51 | 0.21 | 0.26 ± 0.01 | 20.32 ± 1.54 |
| P.AM | 16.68 | 0.24 | 0.34 ± 0.03 | 31.79 ± 2.97 |
| P.SW | 2.79 | 0.32 | 0.42 ± 0.03 | 63.54 ± 11.63 |
| P.AM | 8.37 | 0.28 | 0.35 ± 0.02 | 40.22 ± 2.36 |
| P.MD | 10.02 | 0.28 | 0.38 ± 0.03 | 55.31 ± 6.37 |
| P.TM | 3.69 | 0.23 | 0.34 ± 0.04 | 20.67 ± 2.75 |
| P.ZZ | 6.84 | 0.24 | 0.33 ± 0.03 | 45.16 ± 5.95 |
| L^b^.SR | 6.39 | 0.27 | 0.33 ± 0.02 | 84.25 ± 9.63 |
| L.DM | 3.60 | 0.15 | 0.20 ± 0.01 | 11.58 ± 1.31 |
| L.SV | 6.90 | 0.15 | 0.23 ± 0.01 | 11.76 ± 1.58 |
| L.KB | 26.10 | 0.17 | 0.22 ± 0.02 | 96.51 ± 9.27 |
| L.NR | 8.46 | 0.52 | 0.58 ± 0.03 | 55.75 ± 6.13 |
| N^c^.AL | 6.30 | 0.24 | 0.27 ± 0.02 | 0.61 ± 0.07 |
| N.TM | 10.98 | 0.20 | 0.25 ± 0.02 | 0.62 ± 0.05 |
| N.ZZ | 9.24 | 0.12 | 0.17 ± 0.01 | 0.53 ± 0.06 |
| N.MT | 16.44 | 0.28 | 0.29 ± 0.02 | 0.76 ± 0.05 |
| N.AM | 12.60 | 0.17 | 0.26 ± 0.03 | 0.89 ± 0.09 |

^a^P = pregnant.

^b^L = non-pregnant luteal phase; ovulation induced with exogenous gonadotropins, and no sperm deposited.

^c^N = non-ovulatory control.
