# Supplementary material for: Non-invasive identification of protein biomarkers for early pregnancy diagnosis in the cheetah (Acinonyx jubatus)
Source: PLoS One. 2017 Dec 13;12(12):e0188575. doi: 10.1371/journal.pone.0188575 (PMC5728495; doi:10.1371/journal.pone.0188575)
Supplement: S2 Table — (DOCX) [file pone.0188575.s002.docx]

**S2 Table.**

|  | Accession number | Protein name | Mass (Da) | Peptide matches | Significant peptide matches | Unique peptide matches | Significant unique peptide matches | Protein sequence coverage (%) | Isoelectric point | Exponential modified protein abundance Index |
| --- | --- | --- | --- | --- | --- | --- | --- | --- | --- | --- |
| 1 | gi\|755759641\|ref\|XP_011283693.1\| | PREDICTED: LOW QUALITY PROTEIN: titin [Felis catus] | 4525854 | 763 | 763 | 160 | 160 | 5.2 | 6.19 | 0.21 |
| 2 | gi\|57527809\|ref\|NP_001009252.2\| | aminopeptidase N [Felis catus] | 120525 | 530 | 530 | 38 | 38 | 35.1 | 5.91 | 12.24 |
| 3 | gi\|755797159\|ref\|XP_011288253.1\| | PREDICTED: IgGFc-binding protein [Felis catus] | 347013 | 467 | 467 | 60 | 60 | 28 | 5.23 | 2.07 |
| 4 | gi\|410968346\|ref\|XP_003990668.1\| | PREDICTED: selenium-binding protein 1 [Felis catus] | 59581 | 344 | 344 | 23 | 23 | 57 | 5.93 | 11 |
| 5 | gi\|755773894\|ref\|XP_011285459.1\| | PREDICTED: LOW QUALITY PROTEIN: mucin-2 [Felis catus] | 336641 | 198 | 198 | 32 | 32 | 12.2 | 5.17 | 0.79 |
| 6 | gi\|410953013\|ref\|XP_003983171.1\| | PREDICTED: cationic trypsin [Felis catus] | 30220 | 159 | 159 | 10 | 10 | 41.5 | 8.7 | 36.31 |
| 7 | gi\|410967641\|ref\|XP_003990326.1\| | PREDICTED: calcium-activated chloride channel regulator 1 [Felis catus] | 115448 | 205 | 205 | 35 | 35 | 43.7 | 6.54 | 6.03 |
| 8 | gi\|755740581\|ref\|XP_011281297.1\| | PREDICTED: fibrillin-1 [Felis catus] | 352582 | 207 | 207 | 32 | 32 | 14.1 | 4.79 | 0.9 |
| 9 | gi\|410959337\|ref\|XP_003986267.1\| | PREDICTED: meprin A subunit alpha [Felis catus] | 93521 | 178 | 178 | 19 | 19 | 26 | 5.2 | 3.19 |
| 10 | gi\|410953015\|ref\|XP_003983172.1\| | PREDICTED: trypsin-2 [Felis catus] | 29661 | 164 | 164 | 4 | 4 | 25.9 | 4.82 | 3.23 |
| 11 | gi\|586998392\|ref\|XP_006935789.1\| | PREDICTED: intestinal-type alkaline phosphatase [Felis catus] | 54770 | 100 | 100 | 15 | 15 | 48.9 | 8.68 | 4.25 |
| 12 | gi\|566559804\|ref\|NP_001274485.1\| | transthyretin precursor [Felis catus] | 18171 | 72 | 72 | 5 | 5 | 39.7 | 5.55 | 5.13 |
| 13 | gi\|57977283\|ref\|NP_001009961.1\| | serum albumin precursor [Felis catus] | 82985 | 122 | 122 | 23 | 23 | 35.5 | 5.46 | 4.32 |
| 14 | gi\|587018664\|ref\|XP_006943006.1\| | PREDICTED: polymeric immunoglobulin receptor [Felis catus] | 95652 | 84 | 84 | 12 | 12 | 20.7 | 5.55 | 1.01 |
| 15 | gi\|586979587\|ref\|XP_006929424.1\| | PREDICTED: filamin-C isoform X1 [Felis catus] | 331970 | 116 | 116 | 31 | 31 | 13.1 | 5.75 | 0.61 |
| 16 | gi\|755747281\|ref\|XP_011282111.1\| | PREDICTED: chymotrypsin-like elastase family member 1 isoform X1 [Felis catus] | 31303 | 74 | 74 | 7 | 7 | 40.2 | 9.24 | 3.56 |
| 17 | gi\|755795363\|ref\|XP_006941006.2\| | PREDICTED: kallikrein-1 [Felis catus] | 37104 | 73 | 73 | 6 | 6 | 24 | 5.53 | 1.79 |
| 18 | gi\|586987428\|ref\|XP_006932074.1\| | PREDICTED: ectonucleotide pyrophosphatase/phosphodiesterase family member 3 [Felis catus] | 113471 | 72 | 72 | 11 | 11 | 16.5 | 6.48 | 0.73 |
| 19 | gi\|57618968\|ref\|NP_001009838.1\| | dipeptidyl peptidase 4 [Felis catus] | 98811 | 100 | 100 | 21 | 21 | 25 | 5.59 | 2.53 |
| 20 | gi\|86604717\|ref\|NP_001034545.1\| | angiotensin-converting enzyme 2 precursor [Felis catus] | 104105 | 76 | 76 | 17 | 17 | 27.7 | 5.64 | 1.75 |
| 21 | gi\|755781682\|ref\|XP_011286403.1\| | PREDICTED: meprin A subunit beta [Felis catus] | 102548 | 89 | 89 | 12 | 12 | 21.5 | 5.31 | 1.67 |
| 22 | gi\|755777980\|ref\|XP_011285956.1\| | PREDICTED: LOW QUALITY PROTEIN: deleted in malignant brain tumors 1 protein [Felis catus] | 152414 | 70 | 70 | 13 | 13 | 11.2 | 5.36 | 0.76 |
| 23 | gi\|410957015\|ref\|XP_003985130.1\| | PREDICTED: glutamyl aminopeptidase [Felis catus] | 122471 | 80 | 80 | 22 | 22 | 22.2 | 5.65 | 1.88 |
| 24 | gi\|755779025\|ref\|XP_011286065.1\| | PREDICTED: LOW QUALITY PROTEIN: Ig lambda chain V-I region BL2 [Felis catus] | 28048 | 43 | 43 | 2 | 2 | 13.9 | 6.71 | 0.97 |
| 25 | gi\|755704148\|ref\|XP_011278892.1\| | PREDICTED: maltase-glucoamylase, intestinal [Felis catus] | 223639 | 65 | 65 | 21 | 21 | 12.5 | 5.74 | 0.67 |
| 26 | gi\|410968584\|ref\|XP_003990782.1\| | PREDICTED: lactase-phlorizin hydrolase [Felis catus] | 240465 | 70 | 70 | 21 | 21 | 10.8 | 6.77 | 0.58 |
| 27 | gi\|755799876\|ref\|XP_011288556.1\| | PREDICTED: chymotrypsinogen B-like [Felis catus] | 40774 | 40 | 40 | 6 | 6 | 22.2 | 8.97 | 1.87 |
| 28 | gi\|755712918\|ref\|XP_011279602.1\| | PREDICTED: phospholipase B1, membrane-associated [Felis catus] | 185154 | 56 | 56 | 12 | 12 | 9.5 | 5.92 | 0.4 |
| 29 | gi\|755799802\|ref\|XP_011288550.1\| | PREDICTED: chymotrypsinogen B [Felis catus] | 31269 | 38 | 38 | 5 | 5 | 30.4 | 7.48 | 3.56 |
| 30 | gi\|755740797\|ref\|XP_011281331.1\| | PREDICTED: creatine kinase U-type, mitochondrial [Felis catus] | 51814 | 43 | 43 | 7 | 7 | 19.7 | 8.59 | 1.09 |
| 31 | gi\|755790190\|ref\|XP_011287452.1\| | PREDICTED: keratin, type I cytoskeletal 10 [Felis catus] | 52941 | 32 | 32 | 9 | 9 | 24.3 | 4.77 | 1.25 |
| 32 | gi\|587014614\|ref\|XP_006941518.1\| | PREDICTED: xaa-Pro dipeptidase [Felis catus] | 62995 | 33 | 33 | 8 | 8 | 15 | 5.89 | 0.98 |
| 33 | gi\|587000113\|ref\|XP_006936407.1\| | PREDICTED: carboxypeptidase B [Felis catus] | 55482 | 39 | 39 | 11 | 11 | 27.7 | 6.77 | 2.06 |
| 34 | gi\|755791666\|ref\|XP_011287599.1\| | PREDICTED: angiotensin-converting enzyme isoform X1 [Felis catus] | 97746 | 47 | 47 | 10 | 10 | 13.7 | 6.57 | 0.63 |
| 35 | gi\|410957884\|ref\|XP_003985554.1\| | PREDICTED: cytosol aminopeptidase [Felis catus] | 65141 | 45 | 45 | 16 | 16 | 38.1 | 6.65 | 2.74 |
| 36 | gi\|586990665\|ref\|XP_006933138.1\| | PREDICTED: alpha-1-antitrypsin [Felis catus] | 54996 | 33 | 33 | 5 | 5 | 13.1 | 5.62 | 0.68 |
| 37 | gi\|586989631\|ref\|XP_006932808.1\| | PREDICTED: myosin-7 [Felis catus] | 270150 | 33 | 33 | 8 | 8 | 5.8 | 5.59 | 0.17 |
| 38 | gi\|755765573\|ref\|XP_006936377.2\| | PREDICTED: neprilysin [Felis catus] | 98576 | 39 | 39 | 13 | 13 | 19.6 | 5.48 | 0.97 |
| 39 | gi\|586980623\|ref\|XP_006929794.1\| | PREDICTED: adenosine deaminase isoform X2 [Felis catus] | 46670 | 49 | 49 | 13 | 13 | 29.6 | 5.53 | 3.64 |
| 40 | gi\|410949124\|ref\|XP_003981274.1\| | PREDICTED: cadherin-related family member 2 [Felis catus] | 151082 | 37 | 37 | 12 | 12 | 11.4 | 4.4 | 0.51 |
| 41 | gi\|410983827\|ref\|XP_003998238.1\| | PREDICTED: cadherin-1 [Felis catus] | 106271 | 55 | 55 | 12 | 12 | 13.3 | 4.62 | 0.72 |
| 42 | gi\|410971829\|ref\|XP_003992365.1\| | PREDICTED: interstitial collagenase-like [Felis catus] | 60684 | 35 | 35 | 8 | 8 | 17.6 | 5.24 | 0.88 |
| 43 | gi\|755791670\|ref\|XP_011287600.1\| | PREDICTED: angiotensin-converting enzyme isoform X2 [Felis catus] | 92416 | 42 | 42 | 8 | 8 | 10.5 | 6.26 | 0.51 |
| 44 | gi\|755772832\|ref\|XP_011285314.1\| | PREDICTED: N-acetylated-alpha-linked acidic dipeptidase-like protein [Felis catus] | 84563 | 26 | 26 | 9 | 9 | 18.4 | 5.44 | 0.66 |
| 45 | gi\|755787436\|ref\|XP_006939853.2\| | PREDICTED: myosin-2 [Felis catus] | 272107 | 26 | 26 | 8 | 8 | 4.9 | 5.64 | 0.15 |
| 46 | gi\|755695102\|ref\|XP_011286658.1\| | PREDICTED: complement C3-like [Felis catus] | 205273 | 35 | 35 | 14 | 14 | 10.3 | 6.12 | 0.39 |
| 47 | gi\|755744670\|ref\|XP_011281771.1\| | PREDICTED: alpha-1-antichymotrypsin [Felis catus] | 52251 | 25 | 25 | 7 | 7 | 19.2 | 7.72 | 0.89 |
| 48 | gi\|755745696\|ref\|XP_011281910.1\| | PREDICTED: integrin beta-1 isoform X1 [Felis catus] | 105003 | 36 | 36 | 10 | 10 | 11.5 | 5.33 | 0.58 |
| 49 | gi\|410989433\|ref\|XP_004000966.1\| | PREDICTED: xaa-Pro aminopeptidase 2 [Felis catus] | 82996 | 30 | 30 | 11 | 11 | 20.1 | 6.04 | 0.89 |
| 50 | gi\|410981095\|ref\|XP_003996908.1\| | PREDICTED: keratin, type I cytoskeletal 19 [Felis catus] | 48389 | 29 | 29 | 7 | 7 | 16.3 | 4.92 | 1.2 |
| 51 | gi\|410952867\|ref\|XP_003983099.1\| | PREDICTED: carboxypeptidase A2 [Felis catus] | 52223 | 23 | 23 | 8 | 8 | 23.5 | 5.93 | 1.08 |
| 52 | gi\|112983638\|ref\|NP_001036821.1\| | dipeptidase 1 precursor [Felis catus] | 48060 | 27 | 27 | 8 | 8 | 18.8 | 6.01 | 1.21 |
| 53 | gi\|755774158\|ref\|XP_011285490.1\| | PREDICTED: cadherin-related family member 5 [Felis catus] | 84363 | 29 | 29 | 7 | 7 | 9.4 | 4.6 | 0.57 |
| 54 | gi\|410951463\|ref\|XP_003982416.1\| | PREDICTED: filamin-B isoform X1 [Felis catus] | 319897 | 24 | 24 | 5 | 5 | 2.4 | 5.42 | 0.11 |
| 55 | gi\|755776713\|ref\|XP_011285783.1\| | PREDICTED: aspartate aminotransferase, cytoplasmic [Felis catus] | 51627 | 22 | 22 | 8 | 8 | 18.6 | 7.08 | 1.1 |
| 56 | gi\|410969366\|ref\|XP_003991167.1\| | PREDICTED: fibronectin isoform X6 [Felis catus] | 294420 | 21 | 21 | 5 | 5 | 2.1 | 5.51 | 0.08 |
| 57 | gi\|410971077\|ref\|XP_003992000.1\| | PREDICTED: latexin [Felis catus] | 29766 | 27 | 27 | 4 | 4 | 23.9 | 5.83 | 2.06 |
| 58 | gi\|755697522\|ref\|XP_011289429.1\| | PREDICTED: lactotransferrin [Felis catus] | 84529 | 22 | 22 | 7 | 7 | 13.6 | 7.62 | 0.49 |
| 59 | gi\|410955202\|ref\|XP_003984246.1\| | PREDICTED: lithostathine-like [Felis catus] | 22063 | 25 | 25 | 1 | 1 | 6.2 | 6.93 | 0.9 |
| 60 | gi\|755751065\|ref\|XP_011282588.1\| | PREDICTED: myosin-binding protein C, slow-type [Felis catus] | 156237 | 20 | 20 | 4 | 4 | 1.9 | 5.58 | 0.17 |
| 61 | gi\|586979257\|ref\|XP_006929319.1\| | PREDICTED: cytochrome c [Felis catus] | 16063 | 20 | 20 | 6 | 6 | 49.5 | 9.61 | 6.74 |
| 62 | gi\|57163759\|ref\|NP_001009222.1\| | lysosomal alpha-mannosidase precursor [Felis catus] | 120089 | 31 | 31 | 11 | 11 | 11.8 | 7.42 | 0.55 |
| 63 | gi\|312147375\|ref\|NP_001185857.1\| | haptoglobin precursor [Felis catus] | 45282 | 22 | 22 | 6 | 6 | 19.9 | 5.55 | 0.88 |
| 64 | gi\|410964505\|ref\|XP_003988794.1\| | PREDICTED: keratin, type II cytoskeletal 8 [Felis catus] | 62044 | 20 | 20 | 6 | 6 | 13.1 | 5.6 | 0.59 |
| 65 | gi\|410965016\|ref\|XP_003989048.1\| | PREDICTED: N-acetylglucosamine-6-sulfatase [Felis catus] | 69330 | 28 | 28 | 6 | 6 | 11 | 8.4 | 0.51 |
| 66 | gi\|410952877\|ref\|XP_003983104.1\| | PREDICTED: carboxypeptidase A1 isoform X2 [Felis catus] | 52761 | 23 | 23 | 6 | 6 | 14.1 | 5.84 | 0.88 |
| 67 | gi\|755688916\|ref\|XP_011279149.1\| | PREDICTED: collagen alpha-1(IV) chain [Felis catus] | 181290 | 20 | 20 | 5 | 5 | 4.8 | 8.36 | 0.17 |
| 68 | gi\|755732164\|ref\|XP_011280961.1\| | PREDICTED: superoxide dismutase [Mn], mitochondrial [Felis catus] | 50503 | 20 | 20 | 4 | 4 | 10.5 | 9.35 | 0.46 |
| 69 | gi\|410962659\|ref\|XP_003987886.1\| | PREDICTED: dihydrolipoyllysine-residue succinyltransferase component of 2-oxoglutarate dehydrogenase complex, mitochondrial [Felis catus] | 55586 | 17 | 17 | 3 | 3 | 7 | 9 | 0.29 |
| 70 | gi\|410952034\|ref\|XP_003982694.1\| | PREDICTED: dihydrolipoyl dehydrogenase, mitochondrial [Felis catus] | 63449 | 18 | 18 | 6 | 6 | 13.8 | 7.59 | 0.57 |
| 71 | gi\|410964495\|ref\|XP_003988789.1\| | PREDICTED: keratin, type II cytoskeletal 1 [Felis catus] | 71719 | 21 | 21 | 4 | 4 | 7 | 7.04 | 0.31 |
| 72 | gi\|410987534\|ref\|XP_004000054.1\| | PREDICTED: ribonuclease UK114 [Felis catus] | 16243 | 13 | 13 | 3 | 3 | 29.9 | 7.82 | 1.39 |
| 73 | gi\|410964485\|ref\|XP_003988785.1\| | PREDICTED: keratin, type II cytoskeletal 75 [Felis catus] | 66147 | 13 | 13 | 4 | 4 | 6.5 | 8.16 | 0.34 |
| 74 | gi\|755796690\|ref\|XP_011288203.1\| | PREDICTED: carcinoembryonic antigen-related cell adhesion molecule 1 isoform X1 [Felis catus] | 61602 | 14 | 14 | 3 | 3 | 6.3 | 5.2 | 0.26 |
| 75 | gi\|755704144\|ref\|XP_011278891.1\| | PREDICTED: probable maltase-glucoamylase-like protein [Felis catus] | 311591 | 17 | 17 | 7 | 7 | 4 | 5.27 | 0.13 |
| 76 | gi\|410980935\|ref\|XP_003996829.1\| | PREDICTED: telethonin [Felis catus] | 20680 | 22 | 22 | 3 | 3 | 23.4 | 5.51 | 0.99 |
| 77 | gi\|755773699\|ref\|XP_011285433.1\| | PREDICTED: glutathione S-transferase P [Felis catus] | 26861 | 13 | 13 | 2 | 2 | 11 | 8.34 | 0.7 |
| 78 | gi\|57163839\|ref\|NP_001009307.1\| | glyceraldehyde-3-phosphate dehydrogenase [Felis catus] | 42206 | 21 | 21 | 1 | 1 | 2.4 | 8.21 | 0.25 |
| 79 | gi\|587015111\|ref\|XP_006941698.1\| | PREDICTED: chymotrypsin-like protease CTRL-1 isoform X2 [Felis catus] | 30707 | 15 | 15 | 4 | 4 | 27.7 | 9.1 | 1.17 |
| 80 | gi\|410959962\|ref\|XP_003986567.1\| | PREDICTED: acid sphingomyelinase-like phosphodiesterase 3a [Felis catus] | 57229 | 21 | 21 | 4 | 4 | 10.9 | 5.78 | 0.52 |
| 81 | gi\|755807803\|ref\|XP_011289415.1\| | PREDICTED: nicastrin [Felis catus] | 83853 | 15 | 15 | 7 | 7 | 11.3 | 5.59 | 0.49 |
| 82 | gi\|755693865\|ref\|XP_011285140.1\| | PREDICTED: integrin alpha-2 [Felis catus] | 140246 | 15 | 15 | 4 | 4 | 5.1 | 5.24 | 0.15 |
| 83 | gi\|410972925\|ref\|XP_003992906.1\| | PREDICTED: hemoglobin subunit beta-A/B [Felis catus] | 19159 | 11 | 11 | 3 | 3 | 28.6 | 7.1 | 1.67 |
| 84 | gi\|755822233\|ref\|XP_011278043.1\| | PREDICTED: keratin, type II cytoskeletal 6A, partial [Felis catus] | 56746 | 13 | 13 | 5 | 5 | 9.4 | 5.4 | 0.52 |
| 85 | gi\|410973069\|ref\|XP_003992978.1\| | PREDICTED: hemopexin [Felis catus] | 57279 | 8 | 8 | 2 | 2 | 6.9 | 6.57 | 0.18 |
| 86 | gi\|410987401\|ref\|XP_003999993.1\| | PREDICTED: carbonic anhydrase 1 isoform X1 [Felis catus] | 33194 | 12 | 12 | 5 | 5 | 22.2 | 7.19 | 1.05 |
| 87 | gi\|755765599\|ref\|XP_011284492.1\| | PREDICTED: serotransferrin [Felis catus] | 133794 | 15 | 15 | 5 | 5 | 5.2 | 8.41 | 0.2 |
| 88 | gi\|586998890\|ref\|XP_006935984.1\| | PREDICTED: superoxide dismutase [Cu-Zn] [Felis catus] | 18488 | 10 | 10 | 4 | 4 | 33.3 | 6.28 | 1.77 |
| 89 | gi\|587007294\|ref\|XP_006938893.1\| | PREDICTED: cadherin-2 [Felis catus] | 109022 | 12 | 12 | 3 | 3 | 4.6 | 4.63 | 0.14 |
| 90 | gi\|587016801\|ref\|XP_003998841.2\| | PREDICTED: pancreatic secretory granule membrane major glycoprotein GP2 [Felis catus] | 62967 | 18 | 18 | 8 | 8 | 14.8 | 6.59 | 0.84 |
| 91 | gi\|755753040\|ref\|XP_003989706.2\| | PREDICTED: tissue alpha-L-fucosidase [Felis catus] | 60069 | 17 | 17 | 7 | 7 | 15.4 | 6.2 | 0.75 |
| 92 | gi\|755695108\|ref\|XP_011286660.1\| | PREDICTED: complement C3-like [Felis catus] | 203390 | 11 | 11 | 4 | 4 | 3.2 | 6.01 | 0.13 |
| 93 | gi\|755753697\|ref\|XP_011282920.1\| | PREDICTED: basement membrane-specific heparan sulfate proteoglycan core protein [Felis catus] | 509414 | 11 | 11 | 3 | 3 | 0.6 | 6.09 | 0.04 |
| 94 | gi\|410954481\|ref\|XP_003983893.1\| | PREDICTED: cystatin-C-like [Felis catus] | 19027 | 14 | 14 | 3 | 3 | 28.8 | 8.33 | 1.11 |
| 95 | gi\|410950888\|ref\|XP_003982134.1\| | PREDICTED: cartilage oligomeric matrix protein [Felis catus] | 90189 | 8 | 8 | 2 | 2 | 2.5 | 4.36 | 0.11 |
| 96 | gi\|410964493\|ref\|XP_003988788.1\| | PREDICTED: keratin, type II cytoskeletal 2 epidermal [Felis catus] | 76576 | 10 | 10 | 3 | 3 | 4.2 | 8.52 | 0.21 |
| 97 | gi\|410969322\|ref\|XP_003991145.1\| | PREDICTED: carboxypeptidase O [Felis catus] | 94469 | 11 | 11 | 4 | 4 | 6.1 | 8.38 | 0.29 |
| 98 | gi\|755753611\|ref\|XP_011282904.1\| | PREDICTED: F-box only protein 6 [Felis catus] | 34274 | 10 | 10 | 3 | 3 | 13.7 | 5.52 | 0.52 |
| 99 | gi\|189303530\|ref\|NP_001121543.1\| | chloride anion exchanger precursor [Felis catus] | 31901 | 8 | 8 | 1 | 1 | 4.7 | 4.8 | 0.16 |
| 100 | gi\|410974296\|ref\|XP_003993583.1\| | PREDICTED: 4F2 cell-surface antigen heavy chain [Felis catus] | 64560 | 5 | 5 | 1 | 1 | 3.6 | 5.29 | 0.08 |
| 101 | gi\|410967853\|ref\|XP_003990428.1\| | PREDICTED: alpha-amylase 2B [Felis catus] | 64202 | 13 | 13 | 5 | 5 | 9.8 | 6.68 | 0.45 |
| 102 | gi\|755698816\|ref\|XP_011277887.1\| | PREDICTED: transketolase [Felis catus] | 78266 | 12 | 12 | 3 | 3 | 6.9 | 6.95 | 0.2 |
| 103 | gi\|410960327\|ref\|XP_003986744.1\| | PREDICTED: plasminogen isoform X2 [Felis catus] | 105576 | 7 | 7 | 3 | 3 | 5.2 | 7.16 | 0.15 |
| 104 | gi\|410979342\|ref\|XP_003996044.1\| | PREDICTED: neutrophil gelatinase-associated lipocalin-like [Felis catus] | 34414 | 10 | 10 | 3 | 3 | 10.8 | 5.96 | 0.51 |
| 105 | gi\|586998950\|ref\|XP_006936005.1\| | PREDICTED: amyloid beta A4 protein isoform X1 [Felis catus] | 94096 | 13 | 13 | 2 | 2 | 3 | 4.74 | 0.11 |
| 106 | gi\|586991840\|ref\|XP_006933559.1\| | PREDICTED: alpha-2-macroglobulin [Felis catus] | 185650 | 10 | 10 | 3 | 3 | 2.6 | 5.81 | 0.08 |
| 107 | gi\|410949453\|ref\|XP_003981436.1\| | PREDICTED: LOW QUALITY PROTEIN: glutathione peroxidase 3 [Felis catus] | 28966 | 10 | 10 | 4 | 4 | 21.2 | 8.27 | 0.93 |
| 108 | gi\|587017024\|ref\|XP_006942402.1\| | PREDICTED: 4-aminobutyrate aminotransferase, mitochondrial [Felis catus] | 64599 | 9 | 9 | 4 | 4 | 9.4 | 8.69 | 0.34 |
| 109 | gi\|755758826\|ref\|XP_011283568.1\| | PREDICTED: nebulin isoform X1 [Felis catus] | 1084405 | 15 | 15 | 1 | 1 | 0.1 | 9.09 | 0 |
| 110 | gi\|410982852\|ref\|XP_003997760.1\| | PREDICTED: persulfide dioxygenase ETHE1, mitochondrial [Felis catus] | 27091 | 11 | 11 | 4 | 4 | 19.8 | 5.77 | 1.02 |
| 111 | gi\|755748454\|ref\|XP_004001337.3\| | PREDICTED: keratin, type II cytoskeletal 5 [Felis catus] | 70076 | 12 | 12 | 7 | 7 | 9.9 | 7.59 | 0.61 |
| 112 | gi\|196259807\|ref\|NP_001009333.2\| | beta-hexosaminidase subunit beta [Felis catus] | 63612 | 6 | 6 | 2 | 2 | 5.4 | 6.44 | 0.16 |
| 113 | gi\|182509186\|ref\|NP_001116804.1\| | sucrase-isomaltase, intestinal [Felis catus] | 231560 | 13 | 13 | 6 | 6 | 3.5 | 5.7 | 0.16 |
| 114 | gi\|755807297\|ref\|XP_011289352.1\| | PREDICTED: laminin subunit gamma-1, partial [Felis catus] | 198018 | 10 | 10 | 2 | 2 | 1.8 | 5.03 | 0.05 |
| 115 | gi\|410979206\|ref\|XP_003995976.1\| | PREDICTED: neutrophil gelatinase-associated lipocalin-like isoform X1 [Felis catus] | 25605 | 8 | 8 | 3 | 3 | 20.2 | 5.46 | 0.74 |
| 116 | gi\|410955282\|ref\|XP_003984285.1\| | PREDICTED: fatty acid-binding protein, liver [Felis catus] | 17919 | 8 | 8 | 3 | 3 | 33.1 | 7.79 | 1.21 |
| 117 | gi\|755753175\|ref\|XP_011282860.1\| | PREDICTED: chymotrypsin-like elastase family member 3B [Felis catus] | 33029 | 20 | 20 | 1 | 1 | 3.1 | 5.57 | 0.15 |
| 118 | gi\|587015573\|ref\|XP_006941863.1\| | PREDICTED: N-acetylgalactosamine-6-sulfatase [Felis catus] | 57777 | 12 | 12 | 5 | 5 | 11.8 | 6.36 | 0.51 |
| 119 | gi\|410974061\|ref\|XP_003993466.1\| | PREDICTED: gastric intrinsic factor [Felis catus] | 50284 | 7 | 7 | 3 | 3 | 11.5 | 6.55 | 0.33 |
| 120 | gi\|410949312\|ref\|XP_003981367.1\| | PREDICTED: gastrotropin [Felis catus] | 17719 | 12 | 12 | 3 | 3 | 20.3 | 6.3 | 1.22 |
| 121 | gi\|309951108\|ref\|NP_001185483.1\| | protein AMBP precursor [Felis catus] | 44286 | 6 | 6 | 1 | 1 | 4 | 5.75 | 0.11 |
| 122 | gi\|410953638\|ref\|XP_003983477.1\| | PREDICTED: WAP four-disulfide core domain protein 2 isoform X2 [Felis catus] | 15669 | 7 | 7 | 2 | 2 | 21 | 5.42 | 0.82 |
| 123 | gi\|224994156\|ref\|NP_001116826.1\| | ubiquitin-60S ribosomal protein L40 precursor [Felis catus] | 19129 | 6 | 6 | 2 | 2 | 19.5 | 9.87 | 0.64 |
| 124 | gi\|114326410\|ref\|NP_001041615.1\| | ferritin light chain [Felis catus] | 22439 | 7 | 7 | 2 | 2 | 18.3 | 5.52 | 0.53 |
| 125 | gi\|410958594\|ref\|XP_003985901.1\| | PREDICTED: leukocyte elastase inhibitor [Felis catus] | 50000 | 6 | 6 | 2 | 2 | 6.6 | 5.39 | 0.33 |
| 126 | gi\|755773904\|ref\|XP_011285461.1\| | PREDICTED: mucin-5B [Felis catus] | 413889 | 16 | 16 | 4 | 4 | 1 | 5.54 | 0.07 |
| 127 | gi\|755801251\|ref\|XP_006942027.2\| | PREDICTED: zinc-alpha-2-glycoprotein [Felis catus] | 36679 | 8 | 8 | 6 | 6 | 26.4 | 4.93 | 1.48 |
| 128 | gi\|755757840\|ref\|XP_011283428.1\| | PREDICTED: cathepsin S isoform X1 [Felis catus] | 44950 | 8 | 8 | 4 | 4 | 10.2 | 7.52 | 0.53 |
| 129 | gi\|755805026\|ref\|XP_011289070.1\| | PREDICTED: serine protease 27-like [Felis catus] | 37262 | 14 | 14 | 4 | 4 | 17.8 | 4.89 | 0.67 |
| 130 | gi\|410964501\|ref\|XP_003988792.1\| | PREDICTED: keratin, type II cytoskeletal 79 [Felis catus] | 64892 | 7 | 7 | 3 | 3 | 4.7 | 8.11 | 0.25 |
| 131 | gi\|410981896\|ref\|XP_003997300.1\| | PREDICTED: galectin-3-binding protein [Felis catus] | 66901 | 4 | 4 | 2 | 2 | 4.6 | 4.75 | 0.15 |
| 132 | gi\|586983843\|ref\|XP_006930873.1\| | PREDICTED: fibrinogen alpha chain [Felis catus] | 78480 | 6 | 6 | 1 | 1 | 2 | 6.64 | 0.06 |
| 133 | gi\|410980769\|ref\|XP_003996748.1\| | PREDICTED: collagen alpha-1(I) chain isoform X1 [Felis catus] | 152845 | 8 | 8 | 4 | 4 | 3.1 | 5.75 | 0.13 |
| 134 | gi\|410951169\|ref\|XP_004001410.1\| | PREDICTED: LOW QUALITY PROTEIN: glutathione peroxidase 1 [Felis catus] | 24252 | 6 | 6 | 1 | 1 | 5.3 | 6.73 | 0.22 |
| 135 | gi\|410987405\|ref\|XP_003999995.1\| | PREDICTED: carbonic anhydrase 2 [Felis catus] | 34608 | 5 | 5 | 3 | 3 | 13.1 | 6.87 | 0.51 |
| 136 | gi\|587002983\|ref\|XP_006937403.1\| | PREDICTED: plasma protease C1 inhibitor [Felis catus] | 64245 | 6 | 6 | 2 | 2 | 6.1 | 5.47 | 0.16 |
| 137 | gi\|755797412\|ref\|XP_011288287.1\| | PREDICTED: LOW QUALITY PROTEIN: glucose-6-phosphate isomerase [Felis catus] | 71519 | 7 | 7 | 4 | 4 | 9 | 7.75 | 0.31 |
| 138 | gi\|410957470\|ref\|XP_003985350.1\| | PREDICTED: immunoglobulin J chain [Felis catus] | 20315 | 11 | 11 | 2 | 2 | 9.5 | 4.79 | 0.59 |
| 139 | gi\|410947812\|ref\|XP_003980636.1\| | PREDICTED: olfactory receptor 2A12-like [Felis catus] | 38613 | 6 | 6 | 1 | 1 | 2.9 | 8.47 | 0.13 |
| 140 | gi\|410962651\|ref\|XP_003987882.1\| | PREDICTED: epididymal secretory protein E1 [Felis catus] | 19409 | 8 | 8 | 3 | 3 | 18.8 | 8.52 | 1.07 |
| 141 | gi\|410947494\|ref\|XP_003980481.1\| | PREDICTED: olfactomedin-4 [Felis catus] | 63056 | 8 | 8 | 4 | 4 | 6.5 | 4.95 | 0.46 |
| 142 | gi\|755700303\|ref\|XP_011278526.1\| | PREDICTED: fibulin-2 isoform X1 [Felis catus] | 143033 | 4 | 4 | 1 | 1 | 1.1 | 4.75 | 0.03 |
| 143 | gi\|410970314\|ref\|XP_003991630.1\| | PREDICTED: omega-amidase NIT2 [Felis catus] | 35387 | 4 | 4 | 2 | 2 | 7.1 | 5.88 | 0.31 |
| 144 | gi\|755761351\|ref\|XP_011283930.1\| | PREDICTED: 26S proteasome non-ATPase regulatory subunit 1 [Felis catus] | 123056 | 11 | 11 | 1 | 1 | 1 | 5.25 | 0.08 |
| 145 | gi\|410970194\|ref\|XP_003991573.1\| | PREDICTED: coxsackievirus and adenovirus receptor isoform X2 [Felis catus] | 46047 | 2 | 2 | 1 | 1 | 6.8 | 8.27 | 0.11 |
| 146 | gi\|755696485\|ref\|XP_011288239.1\| | PREDICTED: cytochrome P450 4F22 isoform X1 [Felis catus] | 68558 | 8 | 8 | 1 | 1 | 1.3 | 8.67 | 0.07 |
| 147 | gi\|410966581\|ref\|XP_003989809.1\| | PREDICTED: acid sphingomyelinase-like phosphodiesterase 3b [Felis catus] | 55144 | 6 | 6 | 3 | 3 | 7 | 6.02 | 0.3 |
| 148 | gi\|410948892\|ref\|XP_003981161.1\| | PREDICTED: creatine kinase S-type, mitochondrial [Felis catus] | 53332 | 5 | 5 | 2 | 2 | 5.5 | 8.61 | 0.31 |
| 149 | gi\|410983998\|ref\|XP_003998322.1\| | PREDICTED: chymotrypsinogen B [Felis catus] | 27424 | 8 | 8 | 2 | 2 | 7.5 | 7.63 | 0.68 |
| 150 | gi\|755765419\|ref\|XP_011284468.1\| | PREDICTED: ceruloplasmin [Felis catus] | 141290 | 7 | 7 | 4 | 4 | 4.8 | 5.41 | 0.15 |
| 151 | gi\|755803944\|ref\|XP_011288956.1\| | PREDICTED: deoxyribonuclease-1 [Felis catus] | 34203 | 5 | 5 | 1 | 1 | 3.1 | 5.23 | 0.15 |
| 152 | gi\|755822903\|ref\|XP_011278187.1\| | PREDICTED: immunoglobulin omega chain-like, partial [Felis catus] | 15334 | 4 | 4 | 1 | 1 | 6.9 | 6.37 | 0.36 |
| 153 | gi\|410964647\|ref\|XP_003988864.1\| | PREDICTED: keratin, type II cytoskeletal 2 oral [Felis catus] | 73528 | 7 | 7 | 4 | 4 | 5.2 | 8.38 | 0.3 |
| 154 | gi\|755754367\|ref\|XP_011283013.1\| | PREDICTED: adenylyl cyclase-associated protein 1 isoform X1 [Felis catus] | 61612 | 5 | 5 | 1 | 1 | 2.3 | 7.16 | 0.08 |
| 155 | gi\|410977275\|ref\|XP_003995033.1\| | PREDICTED: macrophage migration inhibitory factor [Felis catus] | 13510 | 5 | 5 | 2 | 2 | 17.4 | 7.74 | 1 |
| 156 | gi\|410958194\|ref\|XP_003985704.1\| | PREDICTED: glutathione peroxidase 6 isoform X1 [Felis catus] | 28927 | 4 | 4 | 2 | 2 | 8.1 | 8.19 | 0.39 |
| 157 | gi\|410985421\|ref\|XP_003999021.1\| | PREDICTED: hemoglobin subunit alpha [Felis catus] | 18176 | 2 | 2 | 2 | 2 | 14.8 | 7.77 | 0.68 |
| 158 | gi\|410947740\|ref\|XP_003980600.1\| | PREDICTED: collagen alpha-2(IV) chain [Felis catus] | 188351 | 3 | 3 | 1 | 1 | 0.9 | 8.93 | 0.03 |
| 159 | gi\|755809147\|ref\|XP_003999831.2\| | PREDICTED: protein S100-A8 [Felis catus] | 12891 | 5 | 5 | 4 | 4 | 30.3 | 5.15 | 3.28 |
| 160 | gi\|325652162\|ref\|NP_001191706.1\| | Fel d 7 allergen precursor [Felis catus] | 24010 | 3 | 3 | 2 | 2 | 12.8 | 4.87 | 0.48 |
| 161 | gi\|755776189\|ref\|XP_011285723.1\| | PREDICTED: tankyrase-2 [Felis catus] | 145785 | 6 | 6 | 1 | 1 | 0.6 | 7.62 | 0.03 |
| 162 | gi\|410983725\|ref\|XP_003998188.1\| | PREDICTED: nuclear transport factor 2 [Felis catus] | 16244 | 5 | 5 | 1 | 1 | 6.3 | 5.1 | 0.34 |
| 163 | gi\|755762615\|ref\|XP_011284119.1\| | PREDICTED: enteropeptidase-like, partial [Felis catus] | 49472 | 8 | 8 | 2 | 2 | 4.9 | 4.51 | 0.21 |
| 164 | gi\|410959353\|ref\|XP_003986275.1\| | PREDICTED: phosphoglycerate kinase 2 [Felis catus] | 54837 | 5 | 5 | 1 | 1 | 1.9 | 8.02 | 0.09 |
| 165 | gi\|587017367\|ref\|XP_006942531.1\| | PREDICTED: acylpyruvase FAHD1, mitochondrial [Felis catus] | 27956 | 5 | 5 | 2 | 2 | 6.8 | 6.96 | 0.4 |
| 166 | gi\|410965731\|ref\|XP_003989395.1\| | PREDICTED: alpha-N-acetylgalactosaminidase [Felis catus] | 51200 | 2 | 2 | 1 | 1 | 3.6 | 5.55 | 0.1 |
| 167 | gi\|586986712\|ref\|XP_006931841.1\| | PREDICTED: bis(5~-adenosyl)-triphosphatase ENPP4 [Felis catus] | 57515 | 4 | 4 | 2 | 2 | 4 | 5.9 | 0.18 |
| 168 | gi\|410965904\|ref\|XP_003989478.1\| | PREDICTED: arylsulfatase A isoform X1 [Felis catus] | 56214 | 3 | 3 | 3 | 3 | 6.5 | 5.3 | 0.29 |
| 169 | gi\|410974180\|ref\|XP_003993525.1\| | PREDICTED: transcobalamin-1 [Felis catus] | 54680 | 3 | 3 | 1 | 1 | 3.7 | 6.99 | 0.3 |
| 170 | gi\|755750150\|ref\|XP_011282463.1\| | PREDICTED: leukotriene A-4 hydrolase [Felis catus] | 79241 | 5 | 5 | 3 | 3 | 4.6 | 6.09 | 0.2 |
| 171 | gi\|586998066\|ref\|XP_006935663.1\| | PREDICTED: aspartyl aminopeptidase isoform X1 [Felis catus] | 59999 | 3 | 3 | 1 | 1 | 2 | 6.21 | 0.08 |
| 172 | gi\|410989713\|ref\|XP_004001103.1\| | PREDICTED: coagulation factor VIII [Felis catus] | 299098 | 6 | 6 | 1 | 1 | 0.3 | 6.29 | 0.02 |
| 173 | gi\|410987424\|ref\|XP_004000002.1\| | PREDICTED: copine-3 [Felis catus] | 69136 | 3 | 3 | 2 | 2 | 3.4 | 5.56 | 0.15 |
| 174 | gi\|410963250\|ref\|XP_003988178.1\| | PREDICTED: cubilin [Felis catus] | 424183 | 4 | 4 | 2 | 2 | 0.4 | 5.29 | 0.02 |
| 175 | gi\|410981097\|ref\|XP_003996909.1\| | PREDICTED: keratin, type I cytoskeletal 14 [Felis catus] | 57477 | 3 | 3 | 3 | 3 | 7.6 | 5.08 | 0.28 |
| 176 | gi\|586999703\|ref\|XP_006936266.1\| | PREDICTED: alpha-2-HS-glycoprotein [Felis catus] | 42906 | 3 | 3 | 2 | 2 | 6.9 | 5.11 | 0.25 |
| 177 | gi\|755741361\|ref\|XP_011281388.1\| | PREDICTED: LOW QUALITY PROTEIN: formin-1 [Felis catus] | 157800 | 6 | 6 | 1 | 1 | 0.7 | 5.55 | 0.03 |
| 178 | gi\|587012579\|ref\|XP_006940733.1\| | PREDICTED: transcription factor MafG [Felis catus] | 22048 | 5 | 5 | 1 | 1 | 5.6 | 10.04 | 0.24 |
| 179 | gi\|410958590\|ref\|XP_003985899.1\| | PREDICTED: serpin B6 isoform X2 [Felis catus] | 49523 | 3 | 3 | 1 | 1 | 5.6 | 5.32 | 0.21 |
| 180 | gi\|755810241\|ref\|XP_011289700.1\| | PREDICTED: cadherin-17 [Felis catus] | 102941 | 6 | 6 | 3 | 3 | 3.7 | 4.88 | 0.15 |
| 181 | gi\|755721557\|ref\|XP_006930874.2\| | PREDICTED: fibrinogen beta chain [Felis catus] | 69261 | 2 | 2 | 1 | 1 | 5.8 | 8.67 | 0.07 |
| 182 | gi\|755721092\|ref\|XP_011279944.1\| | PREDICTED: LOW QUALITY PROTEIN: uncharacterized protein LOC105260498 [Felis catus] | 70766 | 6 | 6 | 1 | 1 | 1.5 | 9.67 | 0.07 |
| 183 | gi\|312147372\|ref\|NP_001185858.1\| | protein S100-A12 [Felis catus] | 12584 | 4 | 4 | 2 | 2 | 19.6 | 4.98 | 1.11 |
| 184 | gi\|755779082\|ref\|XP_011286069.1\| | PREDICTED: sushi domain-containing protein 2 [Felis catus] | 96191 | 3 | 3 | 2 | 2 | 3.4 | 5.82 | 0.1 |
| 185 | gi\|410987068\|ref\|XP_003999830.1\| | PREDICTED: protein S100-A6 [Felis catus] | 12682 | 4 | 4 | 1 | 1 | 8.9 | 5.06 | 0.45 |
| 186 | gi\|410977871\|ref\|XP_003995322.1\| | PREDICTED: cytosolic non-specific dipeptidase [Felis catus] | 61944 | 2 | 2 | 2 | 2 | 7.8 | 5.61 | 0.17 |
| 187 | gi\|587019161\|ref\|XP_006943179.1\| | PREDICTED: NAD(P)H-hydrate epimerase [Felis catus] | 32295 | 5 | 5 | 1 | 1 | 3 | 7.62 | 0.16 |
| 188 | gi\|410969050\|ref\|XP_003991010.1\| | PREDICTED: collagen alpha-1(III) chain [Felis catus] | 153840 | 2 | 2 | 1 | 1 | 0.8 | 6.22 | 0.03 |
| 189 | gi\|410956528\|ref\|XP_003984894.1\| | PREDICTED: cathepsin B [Felis catus] | 42177 | 3 | 3 | 1 | 1 | 2.4 | 5.45 | 0.12 |
| 190 | gi\|587011197\|ref\|XP_006940265.1\| | PREDICTED: nucleoside diphosphate kinase A [Felis catus] | 19979 | 3 | 3 | 2 | 2 | 16.4 | 5.78 | 0.6 |
| 191 | gi\|170763531\|ref\|NP_001116218.2\| | myeloperoxidase precursor [Felis catus] | 86981 | 2 | 2 | 2 | 2 | 3.1 | 9.11 | 0.12 |
| 192 | gi\|755708524\|ref\|XP_003983758.3\| | PREDICTED: attractin [Felis catus] | 174544 | 3 | 3 | 3 | 3 | 1.9 | 7.12 | 0.09 |
| 193 | gi\|586991745\|ref\|XP_006933527.1\| | PREDICTED: triosephosphate isomerase [Felis catus] | 36456 | 2 | 2 | 2 | 2 | 14.7 | 6.15 | 0.3 |
| 194 | gi\|755808188\|ref\|XP_011289446.1\| | PREDICTED: bifunctional glutamate/proline--tRNA ligase [Felis catus] | 205155 | 4 | 4 | 2 | 2 | 1.1 | 7.31 | 0.05 |
| 195 | gi\|755764004\|ref\|XP_011284298.1\| | PREDICTED: mucin-13 [Felis catus] | 66068 | 2 | 2 | 1 | 1 | 1.5 | 5.79 | 0.08 |
| 196 | gi\|410972493\|ref\|XP_003992693.1\| | PREDICTED: dipeptidyl peptidase 1 isoform X1 [Felis catus] | 57337 | 4 | 4 | 1 | 1 | 1.3 | 6.02 | 0.09 |
| 197 | gi\|755781613\|ref\|XP_011286389.1\| | PREDICTED: LOW QUALITY PROTEIN: myomesin-1 [Felis catus] | 207415 | 2 | 2 | 2 | 2 | 1.3 | 6.17 | 0.05 |
| 198 | gi\|755696144\|ref\|XP_011287788.1\| | PREDICTED: resistin [Felis catus] | 16746 | 2 | 2 | 2 | 2 | 13.1 | 9.05 | 0.75 |
| 199 | gi\|57619159\|ref\|NP_001009876.1\| | beta-2-microglobulin precursor [Felis catus] | 15636 | 2 | 2 | 1 | 1 | 10.2 | 6.03 | 0.35 |
| 200 | gi\|755748852\|ref\|XP_011282298.1\| | PREDICTED: mucin-19 [Felis catus] | 718517 | 2 | 2 | 1 | 1 | 0.2 | 5.86 | 0.01 |
| 201 | gi\|410969762\|ref\|XP_003991361.1\| | PREDICTED: THAP domain-containing protein 4 isoform X2 [Felis catus] | 20752 | 3 | 3 | 1 | 1 | 5.5 | 6.22 | 0.26 |
| 202 | gi\|755802258\|ref\|XP_011288823.1\| | PREDICTED: zymogen granule membrane protein 16 [Felis catus] | 19996 | 3 | 3 | 1 | 1 | 4.2 | 8.96 | 0.27 |
| 203 | gi\|586984640\|ref\|XP_006931131.1\| | PREDICTED: vitamin D-binding protein [Felis catus] | 63987 | 2 | 2 | 2 | 2 | 7.8 | 5.07 | 0.16 |
| 204 | gi\|410978857\|ref\|XP_003995804.1\| | PREDICTED: prostaglandin reductase 1 [Felis catus] | 42370 | 1 | 1 | 1 | 1 | 3 | 7.59 | 0.12 |
| 205 | gi\|114326408\|ref\|NP_001041616.1\| | ferritin heavy chain [Felis catus] | 24620 | 1 | 1 | 1 | 1 | 6 | 5.53 | 0.21 |
| 206 | gi\|755784889\|ref\|XP_011286804.1\| | PREDICTED: thioredoxin [Felis catus] | 15085 | 2 | 2 | 1 | 1 | 8.6 | 4.97 | 0.37 |
| 207 | gi\|587010566\|ref\|XP_006940042.1\| | PREDICTED: alpha-2-antiplasmin isoform X1 [Felis catus] | 62034 | 2 | 2 | 2 | 2 | 4 | 6.09 | 0.17 |
| 208 | gi\|410949523\|ref\|XP_003981471.1\| | PREDICTED: integrin alpha-1 isoform X1 [Felis catus] | 148395 | 5 | 5 | 1 | 1 | 0.6 | 5.75 | 0.03 |
| 209 | gi\|410955432\|ref\|XP_003984357.1\| | PREDICTED: aldose 1-epimerase [Felis catus] | 42124 | 1 | 1 | 1 | 1 | 5 | 6.06 | 0.12 |
| 210 | gi\|755707496\|ref\|XP_003983653.3\| | PREDICTED: myosin-7B isoform X1 [Felis catus] | 262039 | 2 | 2 | 2 | 2 | 0.9 | 5.75 | 0.04 |
| 211 | gi\|410987349\|ref\|XP_003999967.1\| | PREDICTED: tumor protein D52 isoform X2 [Felis catus] | 24017 | 1 | 1 | 1 | 1 | 8.2 | 4.98 | 0.22 |
| 212 | gi\|587016752\|ref\|XP_006942304.1\| | PREDICTED: LOW QUALITY PROTEIN: dynein heavy chain 3, axonemal [Felis catus] | 540663 | 4 | 4 | 1 | 1 | 0.2 | 5.61 | 0.01 |
| 213 | gi\|410980361\|ref\|XP_003996546.1\| | PREDICTED: vitronectin [Felis catus] | 57608 | 4 | 4 | 1 | 1 | 1.9 | 4.91 | 0.09 |
| 214 | gi\|587018336\|ref\|XP_006942887.1\| | PREDICTED: apolipoprotein R-like [Felis catus] | 25570 | 4 | 4 | 1 | 1 | 3.7 | 6.79 | 0.2 |
| 215 | gi\|755773471\|ref\|XP_011285391.1\| | PREDICTED: cysteine--tRNA ligase, cytoplasmic isoform X1 [Felis catus] | 109011 | 3 | 3 | 2 | 2 | 1.7 | 6.9 | 0.09 |
| 216 | gi\|57619018\|ref\|NP_001009848.1\| | sodium/calcium exchanger 1 precursor [Felis catus] | 120306 | 2 | 2 | 1 | 1 | 2.9 | 4.91 | 0.04 |
| 217 | gi\|587013627\|ref\|XP_006941142.1\| | PREDICTED: C5a anaphylatoxin chemotactic receptor 1 [Felis catus] | 41394 | 12 | 12 | 1 | 1 | 1.7 | 9.41 | 0.12 |
| 218 | gi\|755765659\|ref\|XP_011284504.1\| | PREDICTED: ceruloplasmin-like [Felis catus] | 134644 | 2 | 2 | 1 | 1 | 0.9 | 5.96 | 0.04 |
| 219 | gi\|755786511\|ref\|XP_006944370.2\| | PREDICTED: dipeptidyl peptidase 2 [Felis catus] | 60734 | 2 | 2 | 2 | 2 | 5 | 6.18 | 0.17 |
| 220 | gi\|755789337\|ref\|XP_011287365.1\| | PREDICTED: ATPase family AAA domain-containing protein 5 [Felis catus] | 258331 | 4 | 4 | 2 | 2 | 0.9 | 9.32 | 0.04 |
| 221 | gi\|410983631\|ref\|XP_003998142.1\| | PREDICTED: aspartate aminotransferase, mitochondrial [Felis catus] | 54772 | 1 | 1 | 1 | 1 | 3.5 | 9.19 | 0.09 |
| 222 | gi\|755699181\|ref\|XP_006928695.2\| | PREDICTED: gamma-interferon-inducible lysosomal thiol reductase [Felis catus] | 35333 | 2 | 2 | 2 | 2 | 8.2 | 5.57 | 0.31 |
| 223 | gi\|587021718\|ref\|XP_004000896.2\| | PREDICTED: lysosome-associated membrane glycoprotein 2 isoform X1 [Felis catus] | 57073 | 3 | 3 | 1 | 1 | 1.7 | 7.43 | 0.09 |
| 224 | gi\|57163917\|ref\|NP_001009370.1\| | peptidyl-prolyl cis-trans isomerase A [Felis catus] | 21295 | 3 | 3 | 2 | 2 | 9.8 | 7.68 | 0.56 |
| 225 | gi\|589811507\|ref\|NP_001277178.1\| | retinol-binding protein 4 precursor [Felis catus] | 25975 | 3 | 3 | 2 | 2 | 15.4 | 5.24 | 0.44 |
| 226 | gi\|586978791\|ref\|XP_006929168.1\| | PREDICTED: neuronal cell adhesion molecule isoform X15 [Felis catus] | 147792 | 3 | 3 | 1 | 1 | 0.7 | 5.51 | 0.03 |
| 227 | gi\|410985002\|ref\|XP_003998814.1\| | PREDICTED: probable glutamate--tRNA ligase, mitochondrial [Felis catus] | 63045 | 9 | 9 | 1 | 1 | 1.1 | 8.82 | 0.08 |
| 228 | gi\|410975010\|ref\|XP_003993931.1\| | PREDICTED: neutral ceramidase [Felis catus] | 91636 | 2 | 2 | 2 | 2 | 3.2 | 7.69 | 0.11 |
| 229 | gi\|755730554\|ref\|XP_011280798.1\| | PREDICTED: LOW QUALITY PROTEIN: midasin [Felis catus] | 710516 | 10 | 10 | 1 | 1 | 0.1 | 5.47 | 0.01 |
| 230 | gi\|586983932\|ref\|XP_006930903.1\| | PREDICTED: methylmalonic aciduria type A protein, mitochondrial isoform X1 [Felis catus] | 54766 | 3 | 3 | 1 | 1 | 2.6 | 9.51 | 0.09 |
| 231 | gi\|755773900\|ref\|XP_011285460.1\| | PREDICTED: LOW QUALITY PROTEIN: mucin-5AC [Felis catus] | 359307 | 2 | 2 | 2 | 2 | 1 | 6.3 | 0.03 |
| 232 | gi\|755747619\|ref\|XP_011282152.1\| | PREDICTED: inositol 1,4,5-trisphosphate receptor type 2 [Felis catus] | 349364 | 2 | 2 | 1 | 1 | 0.3 | 5.97 | 0.01 |
| 233 | gi\|110350007\|ref\|NP_001036027.1\| | major vault protein [Felis catus] | 109611 | 3 | 3 | 1 | 1 | 0.9 | 5.5 | 0.04 |
| 234 | gi\|410969292\|ref\|XP_003991130.1\| | PREDICTED: gamma-crystallin B [Felis catus] | 22250 | 3 | 3 | 2 | 2 | 13.1 | 7.55 | 0.53 |
| 235 | gi\|755812784\|ref\|XP_011289987.1\| | PREDICTED: dystrophin isoform X6 [Felis catus] | 481987 | 3 | 3 | 1 | 1 | 0.2 | 5.62 | 0.01 |
| 236 | gi\|410983563\|ref\|XP_003998108.1\| | PREDICTED: solute carrier family 12 member 3 [Felis catus] | 124501 | 2 | 2 | 1 | 1 | 0.8 | 7.54 | 0.04 |
| 237 | gi\|755730560\|ref\|XP_011280799.1\| | PREDICTED: 5~-nucleotidase, partial [Felis catus] | 68935 | 1 | 1 | 1 | 1 | 1.8 | 6.3 | 0.07 |
| 238 | gi\|410973663\|ref\|XP_003993267.1\| | PREDICTED: prothrombin [Felis catus] | 79353 | 6 | 6 | 1 | 1 | 1 | 5.87 | 0.06 |
| 239 | gi\|755702694\|ref\|XP_011278761.1\| | PREDICTED: cyclin-dependent kinase 13 [Felis catus] | 170801 | 2 | 2 | 1 | 1 | 0.5 | 9.52 | 0.03 |
| 240 | gi\|755793749\|ref\|XP_011287841.1\| | PREDICTED: alpha-1B-glycoprotein [Felis catus] | 65601 | 1 | 1 | 1 | 1 | 4.1 | 6.23 | 0.08 |
| 241 | gi\|410984446\|ref\|XP_003998539.1\| | PREDICTED: transferrin receptor protein 2 [Felis catus] | 93564 | 2 | 2 | 1 | 1 | 0.9 | 5.85 | 0.05 |
| 242 | gi\|755743012\|ref\|XP_011281562.1\| | PREDICTED: nesprin-2 isoform X3 [Felis catus] | 939058 | 6 | 6 | 2 | 2 | 0.2 | 5.2 | 0.01 |
| 243 | gi\|755791438\|ref\|XP_011287568.1\| | PREDICTED: LOW QUALITY PROTEIN: rho GTPase-activating protein 27 [Felis catus] | 99947 | 1 | 1 | 1 | 1 | 0.8 | 5.15 | 0.05 |
| 244 | gi\|587005797\|ref\|XP_003994575.2\| | PREDICTED: fucose mutarotase [Felis catus] | 18804 | 1 | 1 | 1 | 1 | 9.7 | 5.41 | 0.29 |
| 245 | gi\|410977086\|ref\|XP_003994942.1\| | PREDICTED: immunoglobulin omega chain-like [Felis catus] | 18001 | 1 | 1 | 1 | 1 | 6.5 | 6.41 | 0.3 |
| 246 | gi\|586980600\|ref\|XP_006929788.1\| | PREDICTED: elafin [Felis catus] | 13707 | 2 | 2 | 1 | 1 | 7.5 | 9.04 | 0.41 |
| 247 | gi\|410965082\|ref\|XP_003989081.1\| | PREDICTED: lysozyme C [Felis catus] | 20275 | 1 | 1 | 1 | 1 | 6.1 | 9.27 | 0.26 |
| 248 | gi\|755801885\|ref\|XP_011288785.1\| | PREDICTED: mucin-17 [Felis catus] | 264120 | 1 | 1 | 1 | 1 | 0.4 | 4.01 | 0.02 |
| 249 | gi\|410951930\|ref\|XP_003982643.1\| | PREDICTED: probable ATP-dependent RNA helicase DDX56 [Felis catus] | 69403 | 3 | 3 | 1 | 1 | 1.6 | 9.05 | 0.07 |
| 250 | gi\|755757349\|ref\|XP_011283383.1\| | PREDICTED: cold shock domain-containing protein E1 isoform X1 [Felis catus] | 109170 | 2 | 2 | 1 | 1 | 0.7 | 6.1 | 0.04 |
| 251 | gi\|194353917\|ref\|NP_001123862.1\| | trefoil factor 3 precursor [Felis catus] | 9880 | 2 | 2 | 1 | 1 | 20 | 4.32 | 0.6 |
| 252 | gi\|586984721\|ref\|XP_006931157.1\| | PREDICTED: alpha-S2-casein-like [Felis catus] | 31833 | 2 | 2 | 1 | 1 | 3.8 | 5.73 | 0.16 |
| 253 | gi\|755823970\|ref\|XP_011278392.1\| | PREDICTED: 50S ribosomal protein L22, chloroplastic-like [Felis catus] | 15062 | 2 | 2 | 1 | 1 | 6.4 | 10.12 | 0.37 |
| 254 | gi\|755726451\|ref\|XP_011280371.1\| | PREDICTED: zinc finger protein 391 isoform X2 [Felis catus] | 9084 | 4 | 4 | 1 | 1 | 7.6 | 9.3 | 0.66 |
| 255 | gi\|587012447\|ref\|XP_006940691.1\| | PREDICTED: envoplakin [Felis catus] | 253078 | 1 | 1 | 1 | 1 | 0.4 | 6.24 | 0.02 |
| 256 | gi\|410964905\|ref\|XP_003988993.1\| | PREDICTED: methionine--tRNA ligase, cytoplasmic isoform X2 [Felis catus] | 112906 | 3 | 3 | 1 | 1 | 0.9 | 6.19 | 0.04 |
| 257 | gi\|586984768\|ref\|XP_006931174.1\| | PREDICTED: exocyst complex component 1 isoform X3 [Felis catus] | 120812 | 3 | 3 | 1 | 1 | 0.9 | 6.24 | 0.04 |
| 258 | gi\|755801889\|ref\|XP_011288786.1\| | PREDICTED: mucin-12 [Felis catus] | 46499 | 1 | 1 | 1 | 1 | 2.5 | 5.5 | 0.11 |
| 259 | gi\|755794228\|ref\|XP_011287917.1\| | PREDICTED: transmembrane channel-like protein 4 [Felis catus] | 84403 | 1 | 1 | 1 | 1 | 1 | 9.43 | 0.06 |
| 260 | gi\|755791279\|ref\|XP_011287549.1\| | PREDICTED: granulins isoform X1 [Felis catus] | 72885 | 1 | 1 | 1 | 1 | 4.9 | 7.52 | 0.07 |
| 261 | gi\|410948204\|ref\|XP_003980831.1\| | PREDICTED: transforming growth factor-beta-induced protein ig-h3 [Felis catus] | 83461 | 1 | 1 | 1 | 1 | 1 | 6.79 | 0.06 |
| 262 | gi\|410950750\|ref\|XP_003982066.1\| | PREDICTED: tropomyosin alpha-4 chain isoform X1 [Felis catus] | 42069 | 1 | 1 | 1 | 1 | 3.5 | 4.71 | 0.12 |
| 263 | gi\|755688537\|ref\|XP_011278735.1\| | PREDICTED: E3 ubiquitin-protein ligase MYCBP2 isoform X1 [Felis catus] | 596411 | 3 | 3 | 1 | 1 | 0.1 | 6.65 | 0.01 |
| 264 | gi\|410980576\|ref\|XP_003996653.1\| | PREDICTED: carbonic anhydrase 4 isoform X1 [Felis catus] | 41003 | 1 | 1 | 1 | 1 | 8.3 | 8.42 | 0.12 |
| 265 | gi\|296923758\|ref\|NP_001171901.1\| | cystatin-C precursor [Felis catus] | 18152 | 1 | 1 | 1 | 1 | 20.4 | 9.39 | 0.3 |
| 266 | gi\|755761612\|ref\|XP_011283971.1\| | PREDICTED: collagen alpha-3(VI) chain [Felis catus] | 376462 | 2 | 2 | 1 | 1 | 0.2 | 6.08 | 0.01 |
| 267 | gi\|410972157\|ref\|XP_003992527.1\| | PREDICTED: heat shock cognate 71 kDa protein [Felis catus] | 83686 | 1 | 1 | 1 | 1 | 1.1 | 5.37 | 0.06 |
| 268 | gi\|755703932\|ref\|XP_011278873.1\| | PREDICTED: thromboxane-A synthase [Felis catus] | 65632 | 2 | 2 | 1 | 1 | 1.1 | 7.17 | 0.08 |
| 269 | gi\|755716289\|ref\|XP_006930848.2\| | PREDICTED: probable ATP-dependent RNA helicase DDX60 [Felis catus] | 232855 | 1 | 1 | 1 | 1 | 0.5 | 7.85 | 0.02 |
| 270 | gi\|586993676\|ref\|XP_006934150.1\| | PREDICTED: myosin-9 [Felis catus] | 268587 | 1 | 1 | 1 | 1 | 0.4 | 5.57 | 0.02 |
| 271 | gi\|410977708\|ref\|XP_003995243.1\| | PREDICTED: spindle and kinetochore-associated protein 1 [Felis catus] | 35677 | 1 | 1 | 1 | 1 | 2.7 | 6.76 | 0.14 |
| 272 | gi\|410976983\|ref\|XP_003994892.1\| | PREDICTED: D-dopachrome decarboxylase [Felis catus] | 14295 | 1 | 1 | 1 | 1 | 9.3 | 7.82 | 0.39 |
| 273 | gi\|755707146\|ref\|XP_011279140.1\| | PREDICTED: LOW QUALITY PROTEIN: helicase with zinc finger domain 2 [Felis catus] | 338182 | 2 | 2 | 1 | 1 | 0.2 | 8.57 | 0.01 |
| 274 | gi\|587019905\|ref\|XP_006943403.1\| | PREDICTED: brain and acute leukemia cytoplasmic protein [Felis catus] | 11873 | 1 | 1 | 1 | 1 | 16 | 10.04 | 0.48 |
| 275 | gi\|587007386\|ref\|XP_006938924.1\| | PREDICTED: FH1/FH2 domain-containing protein 3 [Felis catus] | 194591 | 1 | 1 | 1 | 1 | 0.5 | 5.4 | 0.02 |
| 276 | gi\|587016311\|ref\|XP_003998652.2\| | PREDICTED: transmembrane protein 248 [Felis catus] | 44617 | 2 | 2 | 1 | 1 | 1.7 | 6.36 | 0.11 |
| 277 | gi\|586997924\|ref\|XP_006935609.1\| | PREDICTED: tensin-1 isoform X6 [Felis catus] | 208060 | 1 | 1 | 1 | 1 | 0.4 | 8.43 | 0.02 |
| 278 | gi\|755810053\|ref\|XP_011289678.1\| | PREDICTED: LOW QUALITY PROTEIN: chromodomain-helicase-DNA-binding protein 7 [Felis catus] | 382996 | 1 | 1 | 1 | 1 | 0.2 | 5.96 | 0.01 |
| 279 | gi\|586976823\|ref\|XP_006928469.1\| | PREDICTED: transcription activator BRG1 isoform X1 [Felis catus] | 215688 | 1 | 1 | 1 | 1 | 0.4 | 7.99 | 0.02 |
| 280 | gi\|410975697\|ref\|XP_003994267.1\| | PREDICTED: myoferlin isoform X2 [Felis catus] | 270226 | 1 | 1 | 1 | 1 | 0.3 | 5.86 | 0.02 |
| 281 | gi\|755809151\|ref\|XP_003999832.2\| | PREDICTED: protein S100-A9 [Felis catus] | 17685 | 1 | 1 | 1 | 1 | 5.2 | 5.87 | 0.31 |
| 282 | gi\|755701286\|ref\|XP_011278632.1\| | PREDICTED: cadherin-related family member 3 [Felis catus] | 105218 | 1 | 1 | 1 | 1 | 0.9 | 5.43 | 0.05 |
| 283 | gi\|755739878\|ref\|XP_003987082.2\| | PREDICTED: cartilage intermediate layer protein 1 isoform X1 [Felis catus] | 147915 | 1 | 1 | 1 | 1 | 0.8 | 8.6 | 0.03 |
| 284 | gi\|410958588\|ref\|XP_003985898.1\| | PREDICTED: ribosyldihydronicotinamide dehydrogenase [quinone] [Felis catus] | 30173 | 1 | 1 | 1 | 1 | 7.8 | 5.31 | 0.17 |
| 285 | gi\|587005967\|ref\|XP_006938427.1\| | PREDICTED: glycosyltransferase 1 domain-containing protein 1 isoform X1 [Felis catus] | 40245 | 1 | 1 | 1 | 1 | 2.1 | 5.44 | 0.13 |
| 286 | gi\|755740613\|ref\|XP_011281305.1\| | PREDICTED: unconventional myosin-Vc [Felis catus] | 249436 | 1 | 1 | 1 | 1 | 0.4 | 7.8 | 0.02 |
| 287 | gi\|410959064\|ref\|XP_003986132.1\| | PREDICTED: lactoylglutathione lyase [Felis catus] | 25255 | 1 | 1 | 1 | 1 | 10.9 | 5.4 | 0.21 |
| 288 | gi\|410960930\|ref\|XP_003987040.1\| | PREDICTED: uveal autoantigen with coiled-coil domains and ankyrin repeats isoform X1 [Felis catus] | 198164 | 1 | 1 | 1 | 1 | 0.6 | 6.33 | 0.02 |
| 289 | gi\|410985907\|ref\|XP_003999257.1\| | PREDICTED: antithrombin-III [Felis catus] | 60978 | 1 | 1 | 1 | 1 | 2.8 | 5.65 | 0.08 |
| 290 | gi\|410956071\|ref\|XP_003984668.1\| | PREDICTED: microtubule-associated tumor suppressor 1 isoform X4 [Felis catus] | 62808 | 1 | 1 | 1 | 1 | 1.4 | 7.62 | 0.08 |
| 291 | gi\|410951049\|ref\|XP_003982214.1\| | PREDICTED: collagen alpha-1(VII) chain [Felis catus] | 318329 | 2 | 2 | 1 | 1 | 0.3 | 6.15 | 0.02 |
| 292 | gi\|755792933\|ref\|XP_011287749.1\| | PREDICTED: ectonucleotide pyrophosphatase/phosphodiesterase family member 7 [Felis catus] | 74030 | 1 | 1 | 1 | 1 | 1.1 | 8.54 | 0.07 |
| 293 | gi\|410977013\|ref\|XP_003994907.1\| | PREDICTED: acetyl-CoA carboxylase 2 isoform X1 [Felis catus] | 306408 | 1 | 1 | 1 | 1 | 0.3 | 6.05 | 0.02 |
| 294 | gi\|410964087\|ref\|XP_003988587.1\| | PREDICTED: DENN domain-containing protein 5B isoform X1 [Felis catus] | 166766 | 2 | 2 | 1 | 1 | 0.5 | 6.21 | 0.03 |
| 295 | gi\|410947680\|ref\|XP_003980571.1\| | PREDICTED: arginine and glutamate-rich protein 1 [Felis catus] | 40988 | 1 | 1 | 1 | 1 | 2.6 | 10.35 | 0.12 |
| 296 | gi\|755765563\|ref\|XP_011284489.1\| | PREDICTED: rho guanine nucleotide exchange factor 26 [Felis catus] | 112591 | 1 | 1 | 1 | 1 | 0.7 | 8.94 | 0.04 |
| 297 | gi\|755750140\|ref\|XP_011282461.1\| | PREDICTED: LOW QUALITY PROTEIN: netrin-4 [Felis catus] | 83831 | 1 | 1 | 1 | 1 | 1 | 8.66 | 0.06 |
| 298 | gi\|67972630\|ref\|NP_001020030.1\| | cytochrome P450 1A1 [Felis catus] | 65599 | 2 | 2 | 1 | 1 | 1.4 | 7.23 | 0.08 |
| 299 | gi\|410986218\|ref\|XP_003999408.1\| | PREDICTED: probable ATP-dependent RNA helicase DDX59 [Felis catus] | 81701 | 2 | 2 | 1 | 1 | 1 | 6.78 | 0.06 |
| 300 | gi\|254750669\|ref\|NP_001157127.1\| | lysosome-associated membrane glycoprotein 1 precursor [Felis catus] | 47997 | 1 | 1 | 1 | 1 | 2.2 | 7.07 | 0.1 |
| 301 | gi\|410981520\|ref\|XP_003997116.1\| | PREDICTED: probable ATP-dependent RNA helicase DDX5 [Felis catus] | 77639 | 2 | 2 | 1 | 1 | 1.3 | 9.06 | 0.06 |
| 302 | gi\|586994145\|ref\|XP_003989618.2\| | PREDICTED: GDH/6PGL endoplasmic bifunctional protein isoform X2 [Felis catus] | 99508 | 1 | 1 | 1 | 1 | 0.7 | 7.97 | 0.05 |
| 303 | gi\|587010956\|ref\|XP_006940177.1\| | PREDICTED: acetyl-CoA carboxylase 1 isoform X3 [Felis catus] | 292667 | 1 | 1 | 1 | 1 | 0.3 | 6.01 | 0.02 |
| 304 | gi\|755740452\|ref\|XP_006932422.2\| | PREDICTED: tyrosine-protein phosphatase non-receptor type 9, partial [Felis catus] | 81621 | 1 | 1 | 1 | 1 | 0.9 | 8.87 | 0.06 |
| 305 | gi\|410971047\|ref\|XP_003991985.1\| | PREDICTED: serpin I2 [Felis catus] | 53323 | 1 | 1 | 1 | 1 | 2 | 5.13 | 0.09 |
| 306 | gi\|755696051\|ref\|XP_011287653.1\| | PREDICTED: LOW QUALITY PROTEIN: protein Hook homolog 2 [Felis catus] | 90993 | 1 | 1 | 1 | 1 | 1.1 | 5.35 | 0.05 |
| 307 | gi\|410951335\|ref\|XP_003982353.1\| | PREDICTED: troponin C, slow skeletal and cardiac muscles [Felis catus] | 21699 | 1 | 1 | 1 | 1 | 6.8 | 4.04 | 0.24 |
| 308 | gi\|410981598\|ref\|XP_003997154.1\| | PREDICTED: ATP-binding cassette sub-family A member 9 [Felis catus] | 207492 | 2 | 2 | 1 | 1 | 0.4 | 7.18 | 0.02 |
| 309 | gi\|410971725\|ref\|XP_003992315.1\| | PREDICTED: EPM2A-interacting protein 1 [Felis catus] | 77378 | 1 | 1 | 1 | 1 | 1 | 6.14 | 0.06 |
| 310 | gi\|587021918\|ref\|XP_006944103.1\| | PREDICTED: HIV Tat-specific factor 1 [Felis catus] | 103494 | 1 | 1 | 1 | 1 | 1 | 4.32 | 0.05 |
| 311 | gi\|755722164\|ref\|XP_003985064.2\| | PREDICTED: membrane-associated progesterone receptor component 2 [Felis catus] | 29303 | 1 | 1 | 1 | 1 | 3.2 | 5.32 | 0.18 |
| 312 | gi\|587002352\|ref\|XP_006937183.1\| | PREDICTED: transcription factor SOX-6 isoform X1 [Felis catus] | 108429 | 1 | 1 | 1 | 1 | 0.7 | 7.32 | 0.05 |
| 313 | gi\|755756854\|ref\|XP_011283334.1\| | PREDICTED: LOW QUALITY PROTEIN: doublesex- and mab-3-related transcription factor A2, partial [Felis catus] | 50851 | 1 | 1 | 1 | 1 | 1.9 | 7.41 | 0.1 |
| 314 | gi\|410958746\|ref\|XP_003985975.1\| | PREDICTED: G patch domain and ankyrin repeat-containing protein 1 [Felis catus] | 42015 | 2 | 2 | 1 | 1 | 1.4 | 9.39 | 0.12 |
| 315 | gi\|410947794\|ref\|XP_003980627.1\| | PREDICTED: ly6/PLAUR domain-containing protein 8 [Felis catus] | 31352 | 1 | 1 | 1 | 1 | 3.1 | 5.31 | 0.16 |
| 316 | gi\|57863763\|ref\|NP_001009845.2\| | interleukin-5 precursor [Felis catus] | 17391 | 1 | 1 | 1 | 1 | 4.5 | 8.64 | 0.31 |
| 317 | gi\|410983038\|ref\|XP_003997851.1\| | PREDICTED: tetratricopeptide repeat protein 9B [Felis catus] | 28484 | 1 | 1 | 1 | 1 | 2.5 | 9.72 | 0.18 |
| 318 | gi\|410952919\|ref\|XP_003983124.1\| | PREDICTED: aldose reductase [Felis catus] | 42558 | 1 | 1 | 1 | 1 | 2.2 | 6.51 | 0.12 |
| 319 | gi\|410969636\|ref\|XP_003991299.1\| | PREDICTED: nucleolin [Felis catus] | 98260 | 2 | 2 | 1 | 1 | 1.1 | 4.6 | 0.05 |
| 320 | gi\|410967474\|ref\|XP_003990244.1\| | PREDICTED: tetratricopeptide repeat protein 22 [Felis catus] | 69566 | 1 | 1 | 1 | 1 | 1 | 5.43 | 0.07 |
| 321 | gi\|114326414\|ref\|NP_001041617.1\| | cullin-4B [Felis catus] | 109180 | 1 | 1 | 1 | 1 | 0.8 | 8.25 | 0.04 |
| 322 | gi\|410958483\|ref\|XP_003985847.1\| | PREDICTED: glucose-fructose oxidoreductase domain-containing protein 1 isoform X1 [Felis catus] | 48098 | 1 | 1 | 1 | 1 | 2.6 | 5.63 | 0.1 |
| 323 | gi\|410970677\|ref\|XP_003991804.1\| | PREDICTED: centrosomal protein of 19 kDa [Felis catus] | 23422 | 1 | 1 | 1 | 1 | 3.7 | 5.38 | 0.22 |
| 324 | gi\|586976665\|ref\|XP_006928422.1\| | PREDICTED: zinc finger protein 317 isoform X1 [Felis catus] | 81552 | 2 | 2 | 1 | 1 | 1 | 9.3 | 0.06 |
| 325 | gi\|755762850\|ref\|XP_011284166.1\| | PREDICTED: enteropeptidase, partial [Felis catus] | 77374 | 1 | 1 | 1 | 1 | 1.1 | 5.21 | 0.06 |
| 326 | gi\|410952288\|ref\|XP_003982813.1\| | PREDICTED: collagen alpha-2(I) chain [Felis catus] | 141899 | 1 | 1 | 1 | 1 | 0.7 | 9.21 | 0.03 |
| 327 | gi\|755792792\|ref\|XP_006940713.2\| | PREDICTED: lysosomal alpha-glucosidase isoform X1 [Felis catus] | 111034 | 1 | 1 | 1 | 1 | 1.1 | 6.2 | 0.04 |
| 328 | gi\|410978282\|ref\|XP_003995524.1\| | PREDICTED: spermatogenesis-associated protein 31A3 [Felis catus] | 178928 | 2 | 2 | 1 | 1 | 0.4 | 9.21 | 0.03 |
| 329 | gi\|755732206\|ref\|XP_011280967.1\| | PREDICTED: monofunctional C1-tetrahydrofolate synthase, mitochondrial [Felis catus] | 118422 | 1 | 1 | 1 | 1 | 0.6 | 6.7 | 0.04 |
| 330 | gi\|410947907\|ref\|XP_003980683.1\| | PREDICTED: zinc finger protein 62 homolog isoform X3 [Felis catus] | 134897 | 1 | 1 | 1 | 1 | 1 | 9.14 | 0.04 |
| 331 | gi\|410985725\|ref\|XP_003999167.1\| | PREDICTED: centrosomal protein of 170 kDa isoform X8 [Felis catus] | 189847 | 1 | 1 | 1 | 1 | 0.5 | 6.75 | 0.03 |
| 332 | gi\|587013050\|ref\|XP_006940923.1\| | PREDICTED: LOW QUALITY PROTEIN: serine/threonine-protein kinase BRSK1 [Felis catus] | 91609 | 1 | 1 | 1 | 1 | 1.1 | 9.39 | 0.05 |
| 333 | gi\|755707777\|ref\|XP_003983593.2\| | PREDICTED: LOW QUALITY PROTEIN: retinoblastoma-like protein 1 [Felis catus] | 137408 | 1 | 1 | 1 | 1 | 0.7 | 6.77 | 0.04 |
| 334 | gi\|587009145\|ref\|XP_006939520.1\| | PREDICTED: 78 kDa glucose-regulated protein [Felis catus] | 86531 | 1 | 1 | 1 | 1 | 1.1 | 5.06 | 0.06 |
| 335 | gi\|587008699\|ref\|XP_006939369.1\| | PREDICTED: hemogen [Felis catus] | 66603 | 1 | 1 | 1 | 1 | 1.4 | 4.83 | 0.07 |
| 336 | gi\|410975647\|ref\|XP_003994242.1\| | PREDICTED: ras-GEF domain-containing family member 1A isoform X3 [Felis catus] | 64311 | 1 | 1 | 1 | 1 | 1.2 | 7.95 | 0.08 |
| 337 | gi\|146198809\|ref\|NP_001078907.1\| | adiponectin precursor [Felis catus] | 29778 | 1 | 1 | 1 | 1 | 3.7 | 5.68 | 0.17 |
| 338 | gi\|586999293\|ref\|XP_006936120.1\| | PREDICTED: D(3) dopamine receptor [Felis catus] | 47576 | 1 | 1 | 1 | 1 | 1.5 | 9.36 | 0.11 |
| 339 | gi\|410958730\|ref\|XP_003985967.1\| | PREDICTED: allograft inflammatory factor 1 isoform X1 [Felis catus] | 21402 | 1 | 1 | 1 | 1 | 4.1 | 7.85 | 0.25 |
| 340 | gi\|587012884\|ref\|XP_006940858.1\| | PREDICTED: zinc finger protein 547 isoform X1 [Felis catus] | 70799 | 2 | 2 | 1 | 1 | 0.9 | 8.56 | 0.07 |
| 341 | gi\|755699397\|ref\|XP_006928978.2\| | PREDICTED: THO complex subunit 7 homolog [Felis catus] | 28767 | 1 | 1 | 1 | 1 | 2.9 | 5.57 | 0.18 |
| 342 | gi\|755789464\|ref\|XP_011287389.1\| | PREDICTED: mediator of RNA polymerase II transcription subunit 13 [Felis catus] | 267397 | 2 | 2 | 1 | 1 | 0.6 | 5.4 | 0.02 |
| 343 | gi\|755724163\|ref\|XP_006931168.2\| | PREDICTED: testicular haploid expressed gene protein-like [Felis catus] | 64494 | 1 | 1 | 1 | 1 | 1.2 | 8.51 | 0.08 |
| 344 | gi\|410969535\|ref\|XP_003991250.1\| | PREDICTED: phenylalanine--tRNA ligase beta subunit [Felis catus] | 77382 | 1 | 1 | 1 | 1 | 1.2 | 6.67 | 0.06 |
| 345 | gi\|410947702\|ref\|XP_003980582.1\| | PREDICTED: coagulation factor VII isoform X1 [Felis catus] | 54518 | 1 | 1 | 1 | 1 | 1.3 | 5.81 | 0.09 |
| 346 | gi\|410984235\|ref\|XP_003998435.1\| | PREDICTED: G-protein coupled estrogen receptor 1 [Felis catus] | 43889 | 1 | 1 | 1 | 1 | 1.7 | 8.3 | 0.11 |
| 347 | gi\|755790754\|ref\|XP_011287490.1\| | PREDICTED: integrin alpha-3 [Felis catus] | 124337 | 2 | 2 | 1 | 1 | 0.5 | 6.25 | 0.04 |
| 348 | gi\|755775779\|ref\|XP_011285671.1\| | PREDICTED: catenin alpha-3 [Felis catus] | 117282 | 1 | 1 | 1 | 1 | 0.8 | 6.02 | 0.04 |
| 349 | gi\|114326435\|ref\|NP_001041626.1\| | MHC class I antigen precursor [Felis catus] | 43829 | 1 | 1 | 1 | 1 | 2.5 | 5.66 | 0.11 |
| 350 | gi\|410963228\|ref\|XP_003988167.1\| | PREDICTED: protein artemis isoform X1 [Felis catus] | 88844 | 1 | 1 | 1 | 1 | 0.7 | 5.71 | 0.06 |
| 351 | gi\|410976593\|ref\|XP_003994702.1\| | PREDICTED: actin-related protein 2/3 complex subunit 3 [Felis catus] | 25116 | 1 | 1 | 1 | 1 | 3.9 | 8.78 | 0.21 |
| 352 | gi\|587009684\|ref\|XP_006939715.1\| | PREDICTED: uncharacterized protein LOC101092651 [Felis catus] | 142190 | 1 | 1 | 1 | 1 | 0.4 | 10.64 | 0.03 |
| 353 | gi\|755761090\|ref\|XP_011283895.1\| | PREDICTED: period circadian protein homolog 2 isoform X1 [Felis catus] | 149623 | 1 | 1 | 1 | 1 | 0.4 | 6.35 | 0.03 |
| 354 | gi\|587022443\|ref\|XP_006944293.1\| | PREDICTED: PEX5-related protein isoform X1 [Felis catus] | 82262 | 1 | 1 | 1 | 1 | 1.7 | 5.2 | 0.06 |
| 355 | gi\|410967424\|ref\|XP_003990219.1\| | PREDICTED: interleukin-12 receptor subunit beta-2 isoform X4 [Felis catus] | 82171 | 6 | 6 | 1 | 1 | 0.8 | 8.82 | 0.06 |
| 356 | gi\|755724465\|ref\|XP_011280199.1\| | PREDICTED: ADP-ribosylation factor-like protein 9 [Felis catus] | 29340 | 1 | 1 | 1 | 1 | 2.7 | 5.53 | 0.18 |
| 357 | gi\|410947364\|ref\|XP_003980419.1\| | PREDICTED: cytidine and dCMP deaminase domain-containing protein 1 [Felis catus] | 68783 | 1 | 1 | 1 | 1 | 1 | 8 | 0.09 |
| 358 | gi\|755741661\|ref\|XP_011281421.1\| | PREDICTED: chromodomain-helicase-DNA-binding protein 8 isoform X1 [Felis catus] | 332396 | 1 | 1 | 1 | 1 | 0.2 | 6.03 | 0.01 |
| 359 | gi\|410956520\|ref\|XP_003984890.1\| | PREDICTED: protein FAM167A [Felis catus] | 25975 | 1 | 1 | 1 | 1 | 2.9 | 5.04 | 0.2 |
| 360 | gi\|410965747\|ref\|XP_003989403.1\| | PREDICTED: polymerase delta-interacting protein 3 [Felis catus] | 55293 | 1 | 1 | 1 | 1 | 1.4 | 10 | 0.09 |
| 361 | gi\|755696151\|ref\|XP_011287799.1\| | PREDICTED: mucin-16 [Felis catus] | 776943 | 1 | 1 | 1 | 1 | 0.1 | 5.17 | 0.01 |
| 362 | gi\|755713432\|ref\|XP_011279649.1\| | PREDICTED: myelin transcription factor 1-like protein [Felis catus] | 152403 | 1 | 1 | 1 | 1 | 0.4 | 4.91 | 0.03 |
| 363 | gi\|410986701\|ref\|XP_003999648.1\| | PREDICTED: GPI mannosyltransferase 1 [Felis catus] | 52511 | 1 | 1 | 1 | 1 | 1.2 | 9.52 | 0.1 |
| 364 | gi\|410953234\|ref\|XP_003983279.1\| | PREDICTED: chondroitin sulfate glucuronyltransferase [Felis catus] | 90351 | 1 | 1 | 1 | 1 | 1.2 | 7.1 | 0.05 |
| 365 | gi\|755807406\|ref\|XP_011289380.1\| | PREDICTED: LEM domain-containing protein 1 [Felis catus] | 27205 | 1 | 1 | 1 | 1 | 3 | 9.59 | 0.19 |
| 366 | gi\|586991079\|ref\|XP_006933287.1\| | PREDICTED: CUGBP Elav-like family member 2 isoform X1 [Felis catus] | 58363 | 1 | 1 | 1 | 1 | 1.4 | 8.84 | 0.09 |
| 367 | gi\|755782743\|ref\|XP_011286532.1\| | PREDICTED: histone-arginine methyltransferase CARM1-like [Felis catus] | 52630 | 1 | 1 | 1 | 1 | 1.7 | 6.31 | 0.09 |
