# Supplementary material for: Non-invasive identification of protein biomarkers for early pregnancy diagnosis in the cheetah (Acinonyx jubatus)
Source: PLoS One. 2017 Dec 13;12(12):e0188575. doi: 10.1371/journal.pone.0188575 (PMC5728495; doi:10.1371/journal.pone.0188575)
Supplement: S3 Table — (DOCX) [file pone.0188575.s003.docx]

**S3 Table.**

|  | Accession number | Protein name | Mass (Da) | Peptide matches | Significant peptide matches | Unique peptide matches | Significant unique peptide matches | Protein sequence coverage (%) | Isoelectric point | Exponential modified protein abundance Index |
| --- | --- | --- | --- | --- | --- | --- | --- | --- | --- | --- |
| 1 | gi\|755759641\|ref\|XP_011283693.1\| | PREDICTED: LOW QUALITY PROTEIN: titin [Felis catus] | 4525854 | 1211 | 956 | 238 | 196 | 7.2 | 6.19 | 0.25 |
| 2 | gi\|755797159\|ref\|XP_011288253.1\| | PREDICTED: IgGFc-binding protein [Felis catus] | 347013 | 418 | 370 | 63 | 61 | 28.1 | 5.23 | 1.78 |
| 3 | gi\|410968346\|ref\|XP_003990668.1\| | PREDICTED: selenium-binding protein 1 [Felis catus] | 59581 | 353 | 307 | 30 | 27 | 58.9 | 5.93 | 10.07 |
| 4 | gi\|755740581\|ref\|XP_011281297.1\| | PREDICTED: fibrillin-1 [Felis catus] | 352582 | 295 | 274 | 42 | 37 | 16.6 | 4.79 | 1 |
| 5 | gi\|57527809\|ref\|NP_001009252.2\| | aminopeptidase N [Felis catus] | 120525 | 372 | 298 | 30 | 30 | 27.4 | 5.91 | 4.75 |
| 6 | gi\|410967641\|ref\|XP_003990326.1\| | PREDICTED: calcium-activated chloride channel regulator 1 [Felis catus] | 115448 | 246 | 198 | 31 | 26 | 36.9 | 6.54 | 2.47 |
| 7 | gi\|410953013\|ref\|XP_003983171.1\| | PREDICTED: cationic trypsin [Felis catus] | 30220 | 152 | 139 | 10 | 9 | 37.8 | 8.7 | 11.4 |
| 8 | gi\|755773894\|ref\|XP_011285459.1\| | PREDICTED: LOW QUALITY PROTEIN: mucin-2 [Felis catus] | 336641 | 187 | 163 | 27 | 22 | 10 | 5.17 | 0.45 |
| 9 | gi\|410953015\|ref\|XP_003983172.1\| | PREDICTED: trypsin-2 [Felis catus] | 29661 | 176 | 149 | 5 | 5 | 24.7 | 4.82 | 2.6 |
| 10 | gi\|586979587\|ref\|XP_006929424.1\| | PREDICTED: filamin-C isoform X1 [Felis catus] | 331970 | 193 | 166 | 37 | 36 | 13.4 | 5.75 | 0.76 |
| 11 | gi\|587018664\|ref\|XP_006943006.1\| | PREDICTED: polymeric immunoglobulin receptor [Felis catus] | 95652 | 160 | 136 | 17 | 17 | 24.1 | 5.55 | 1.46 |
| 12 | gi\|755787436\|ref\|XP_006939853.2\| | PREDICTED: myosin-2 [Felis catus] | 272107 | 109 | 85 | 13 | 9 | 6.2 | 5.64 | 0.19 |
| 13 | gi\|586989631\|ref\|XP_006932808.1\| | PREDICTED: myosin-7 [Felis catus] | 270150 | 106 | 82 | 13 | 9 | 6.6 | 5.59 | 0.22 |
| 14 | gi\|566559804\|ref\|NP_001274485.1\| | transthyretin precursor [Felis catus] | 18171 | 84 | 64 | 7 | 6 | 45.9 | 5.55 | 5.13 |
| 15 | gi\|57977283\|ref\|NP_001009961.1\| | serum albumin precursor [Felis catus] | 82985 | 104 | 99 | 21 | 20 | 30.9 | 5.46 | 2.55 |
| 16 | gi\|410959337\|ref\|XP_003986267.1\| | PREDICTED: meprin A subunit alpha [Felis catus] | 93521 | 97 | 80 | 14 | 11 | 22.3 | 5.2 | 1.05 |
| 17 | gi\|755777980\|ref\|XP_011285956.1\| | PREDICTED: LOW QUALITY PROTEIN: deleted in malignant brain tumors 1 protein [Felis catus] | 152414 | 83 | 77 | 12 | 11 | 9.8 | 5.36 | 0.6 |
| 18 | gi\|755795363\|ref\|XP_006941006.2\| | PREDICTED: kallikrein-1 [Felis catus] | 37104 | 90 | 83 | 6 | 5 | 21.3 | 5.53 | 1.46 |
| 19 | gi\|57618968\|ref\|NP_001009838.1\| | dipeptidyl peptidase 4 [Felis catus] | 98811 | 115 | 81 | 24 | 20 | 25.2 | 5.59 | 1.9 |
| 20 | gi\|410968584\|ref\|XP_003990782.1\| | PREDICTED: lactase-phlorizin hydrolase [Felis catus] | 240465 | 112 | 91 | 25 | 19 | 12.3 | 6.77 | 0.52 |
| 21 | gi\|755790190\|ref\|XP_011287452.1\| | PREDICTED: keratin, type I cytoskeletal 10 [Felis catus] | 52941 | 70 | 58 | 12 | 9 | 24.8 | 4.77 | 1.7 |
| 22 | gi\|755747281\|ref\|XP_011282111.1\| | PREDICTED: chymotrypsin-like elastase family member 1 isoform X1 [Felis catus] | 31303 | 70 | 65 | 7 | 5 | 39.8 | 9.24 | 2.37 |
| 23 | gi\|410957015\|ref\|XP_003985130.1\| | PREDICTED: glutamyl aminopeptidase [Felis catus] | 122471 | 97 | 77 | 23 | 19 | 19.9 | 5.65 | 1.37 |
| 24 | gi\|86604717\|ref\|NP_001034545.1\| | angiotensin-converting enzyme 2 precursor [Felis catus] | 104105 | 81 | 62 | 18 | 15 | 25.3 | 5.64 | 1.29 |
| 25 | gi\|410957884\|ref\|XP_003985554.1\| | PREDICTED: cytosol aminopeptidase [Felis catus] | 65141 | 80 | 58 | 22 | 19 | 40.6 | 6.65 | 3.34 |
| 26 | gi\|586987428\|ref\|XP_006932074.1\| | PREDICTED: ectonucleotide pyrophosphatase/phosphodiesterase family member 3 [Felis catus] | 113471 | 77 | 58 | 15 | 11 | 18 | 6.48 | 0.73 |
| 27 | gi\|586998392\|ref\|XP_006935789.1\| | PREDICTED: intestinal-type alkaline phosphatase [Felis catus] | 54770 | 52 | 47 | 11 | 10 | 31.9 | 8.68 | 2.39 |
| 28 | gi\|587000113\|ref\|XP_006936407.1\| | PREDICTED: carboxypeptidase B [Felis catus] | 55482 | 72 | 50 | 13 | 11 | 32.3 | 6.77 | 2.06 |
| 29 | gi\|755799802\|ref\|XP_011288550.1\| | PREDICTED: chymotrypsinogen B [Felis catus] | 31269 | 42 | 35 | 6 | 6 | 30.4 | 7.48 | 2.37 |
| 30 | gi\|586980623\|ref\|XP_006929794.1\| | PREDICTED: adenosine deaminase isoform X2 [Felis catus] | 46670 | 64 | 56 | 9 | 9 | 21 | 5.53 | 2.08 |
| 31 | gi\|755688916\|ref\|XP_011279149.1\| | PREDICTED: collagen alpha-1(IV) chain [Felis catus] | 181290 | 53 | 49 | 5 | 5 | 3.6 | 8.36 | 0.27 |
| 32 | gi\|755712918\|ref\|XP_011279602.1\| | PREDICTED: phospholipase B1, membrane-associated [Felis catus] | 185154 | 59 | 44 | 12 | 10 | 9 | 5.92 | 0.3 |
| 33 | gi\|410964495\|ref\|XP_003988789.1\| | PREDICTED: keratin, type II cytoskeletal 1 [Felis catus] | 71719 | 32 | 29 | 8 | 7 | 10.6 | 7.04 | 0.7 |
| 34 | gi\|410983827\|ref\|XP_003998238.1\| | PREDICTED: cadherin-1 [Felis catus] | 106271 | 81 | 68 | 16 | 14 | 13.6 | 4.62 | 0.88 |
| 35 | gi\|755790971\|ref\|XP_011287513.1\| | PREDICTED: keratin, type I cytoskeletal 13 [Felis catus] | 56157 | 38 | 29 | 9 | 5 | 14.2 | 4.97 | 0.67 |
| 36 | gi\|755704148\|ref\|XP_011278892.1\| | PREDICTED: maltase-glucoamylase, intestinal [Felis catus] | 223639 | 52 | 40 | 16 | 12 | 7.5 | 5.74 | 0.32 |
| 37 | gi\|755791670\|ref\|XP_011287600.1\| | PREDICTED: angiotensin-converting enzyme isoform X2 [Felis catus] | 92416 | 53 | 43 | 10 | 7 | 11.2 | 6.26 | 0.51 |
| 38 | gi\|755697522\|ref\|XP_011289429.1\| | PREDICTED: lactotransferrin [Felis catus] | 84529 | 40 | 33 | 8 | 8 | 12.8 | 7.62 | 0.57 |
| 39 | gi\|410971829\|ref\|XP_003992365.1\| | PREDICTED: interstitial collagenase-like [Felis catus] | 60684 | 38 | 36 | 10 | 10 | 20.6 | 5.24 | 1.2 |
| 40 | gi\|755781682\|ref\|XP_011286403.1\| | PREDICTED: meprin A subunit beta [Felis catus] | 102548 | 49 | 42 | 8 | 8 | 11.1 | 5.31 | 0.83 |
| 41 | gi\|410964505\|ref\|XP_003988794.1\| | PREDICTED: keratin, type II cytoskeletal 8 [Felis catus] | 62044 | 35 | 27 | 6 | 6 | 13.1 | 5.6 | 0.71 |
| 42 | gi\|755779025\|ref\|XP_011286065.1\| | PREDICTED: LOW QUALITY PROTEIN: Ig lambda chain V-I region BL2 [Felis catus] | 28048 | 26 | 22 | 3 | 2 | 16.8 | 6.71 | 0.66 |
| 43 | gi\|755790084\|ref\|XP_003996910.3\| | PREDICTED: keratin, type I cytoskeletal 16 [Felis catus] | 57216 | 43 | 29 | 8 | 6 | 14.4 | 5.1 | 0.95 |
| 44 | gi\|410952034\|ref\|XP_003982694.1\| | PREDICTED: dihydrolipoyl dehydrogenase, mitochondrial [Felis catus] | 63449 | 34 | 28 | 9 | 7 | 15.3 | 7.59 | 0.69 |
| 45 | gi\|755791666\|ref\|XP_011287599.1\| | PREDICTED: angiotensin-converting enzyme isoform X1 [Felis catus] | 97746 | 42 | 30 | 11 | 7 | 12.5 | 6.57 | 0.48 |
| 46 | gi\|755776713\|ref\|XP_011285783.1\| | PREDICTED: aspartate aminotransferase, cytoplasmic [Felis catus] | 51627 | 36 | 30 | 11 | 9 | 23.7 | 7.08 | 1.3 |
| 47 | gi\|410981097\|ref\|XP_003996909.1\| | PREDICTED: keratin, type I cytoskeletal 14 [Felis catus] | 57477 | 50 | 30 | 10 | 6 | 18.4 | 5.08 | 0.79 |
| 48 | gi\|755799876\|ref\|XP_011288556.1\| | PREDICTED: chymotrypsinogen B-like [Felis catus] | 40774 | 36 | 30 | 6 | 6 | 27.5 | 8.97 | 1.55 |
| 49 | gi\|410964493\|ref\|XP_003988788.1\| | PREDICTED: keratin, type II cytoskeletal 2 epidermal [Felis catus] | 76576 | 30 | 27 | 9 | 8 | 13.2 | 8.52 | 0.65 |
| 50 | gi\|410981095\|ref\|XP_003996908.1\| | PREDICTED: keratin, type I cytoskeletal 19 [Felis catus] | 48389 | 30 | 23 | 9 | 6 | 19.5 | 4.92 | 1.2 |
| 51 | gi\|755740797\|ref\|XP_011281331.1\| | PREDICTED: creatine kinase U-type, mitochondrial [Felis catus] | 51814 | 38 | 34 | 9 | 8 | 21.8 | 8.59 | 1.09 |
| 52 | gi\|755695102\|ref\|XP_011286658.1\| | PREDICTED: complement C3-like [Felis catus] | 205273 | 29 | 25 | 10 | 8 | 7.2 | 6.12 | 0.21 |
| 53 | gi\|587016801\|ref\|XP_003998841.2\| | PREDICTED: pancreatic secretory granule membrane major glycoprotein GP2 [Felis catus] | 62967 | 40 | 28 | 7 | 6 | 12.5 | 6.59 | 0.58 |
| 54 | gi\|112983638\|ref\|NP_001036821.1\| | dipeptidase 1 precursor [Felis catus] | 48060 | 27 | 22 | 9 | 7 | 21 | 6.01 | 1 |
| 55 | gi\|755822233\|ref\|XP_011278043.1\| | PREDICTED: keratin, type II cytoskeletal 6A, partial [Felis catus] | 56746 | 34 | 27 | 11 | 8 | 19.6 | 5.4 | 0.96 |
| 56 | gi\|410947740\|ref\|XP_003980600.1\| | PREDICTED: collagen alpha-2(IV) chain [Felis catus] | 188351 | 24 | 23 | 3 | 3 | 2.5 | 8.93 | 0.14 |
| 57 | gi\|410964485\|ref\|XP_003988785.1\| | PREDICTED: keratin, type II cytoskeletal 75 [Felis catus] | 66147 | 23 | 18 | 6 | 4 | 9.3 | 8.16 | 0.34 |
| 58 | gi\|410989631\|ref\|XP_004001062.1\| | PREDICTED: filamin-A isoform X1 [Felis catus] | 320115 | 28 | 21 | 6 | 6 | 2 | 5.72 | 0.11 |
| 59 | gi\|410949453\|ref\|XP_003981436.1\| | PREDICTED: LOW QUALITY PROTEIN: glutathione peroxidase 3 [Felis catus] | 28966 | 24 | 22 | 5 | 4 | 24.3 | 8.27 | 0.93 |
| 60 | gi\|410949124\|ref\|XP_003981274.1\| | PREDICTED: cadherin-related family member 2 [Felis catus] | 151082 | 26 | 26 | 8 | 8 | 5.1 | 4.4 | 0.33 |
| 61 | gi\|410952867\|ref\|XP_003983099.1\| | PREDICTED: carboxypeptidase A2 [Felis catus] | 52223 | 27 | 24 | 7 | 7 | 21.3 | 5.93 | 0.9 |
| 62 | gi\|410981896\|ref\|XP_003997300.1\| | PREDICTED: galectin-3-binding protein [Felis catus] | 66901 | 20 | 17 | 4 | 4 | 10 | 4.75 | 0.33 |
| 63 | gi\|410964499\|ref\|XP_003988791.1\| | PREDICTED: keratin, type II cytoskeletal 4 [Felis catus] | 64632 | 26 | 19 | 8 | 6 | 13.4 | 6.92 | 0.56 |
| 64 | gi\|755745696\|ref\|XP_011281910.1\| | PREDICTED: integrin beta-1 isoform X1 [Felis catus] | 105003 | 32 | 25 | 11 | 9 | 12.6 | 5.33 | 0.51 |
| 65 | gi\|114326410\|ref\|NP_001041615.1\| | ferritin light chain [Felis catus] | 22439 | 20 | 19 | 5 | 5 | 39.4 | 5.52 | 1.87 |
| 66 | gi\|410954481\|ref\|XP_003983893.1\| | PREDICTED: cystatin-C-like [Felis catus] | 19027 | 27 | 25 | 6 | 6 | 32.4 | 8.33 | 3.45 |
| 67 | gi\|755751065\|ref\|XP_011282588.1\| | PREDICTED: myosin-binding protein C, slow-type [Felis catus] | 156237 | 16 | 16 | 3 | 3 | 1.9 | 5.58 | 0.13 |
| 68 | gi\|57163759\|ref\|NP_001009222.1\| | lysosomal alpha-mannosidase precursor [Felis catus] | 120089 | 31 | 24 | 7 | 7 | 6.1 | 7.42 | 0.32 |
| 69 | gi\|57163839\|ref\|NP_001009307.1\| | glyceraldehyde-3-phosphate dehydrogenase [Felis catus] | 42206 | 35 | 23 | 2 | 1 | 3.9 | 8.21 | 0.25 |
| 70 | gi\|410969322\|ref\|XP_003991145.1\| | PREDICTED: carboxypeptidase O [Felis catus] | 94469 | 31 | 24 | 6 | 6 | 9.1 | 8.38 | 0.43 |
| 71 | gi\|755695108\|ref\|XP_011286660.1\| | PREDICTED: complement C3-like [Felis catus] | 203390 | 14 | 13 | 3 | 2 | 2.1 | 6.01 | 0.07 |
| 72 | gi\|410952877\|ref\|XP_003983104.1\| | PREDICTED: carboxypeptidase A1 isoform X2 [Felis catus] | 52761 | 20 | 19 | 6 | 6 | 17.6 | 5.84 | 0.72 |
| 73 | gi\|410960327\|ref\|XP_003986744.1\| | PREDICTED: plasminogen isoform X2 [Felis catus] | 105576 | 20 | 13 | 5 | 2 | 5.4 | 7.16 | 0.09 |
| 74 | gi\|410987401\|ref\|XP_003999993.1\| | PREDICTED: carbonic anhydrase 1 isoform X1 [Felis catus] | 33194 | 13 | 13 | 3 | 3 | 10.7 | 7.19 | 0.54 |
| 75 | gi\|586979257\|ref\|XP_006929319.1\| | PREDICTED: cytochrome c [Felis catus] | 16063 | 20 | 19 | 3 | 3 | 33.3 | 9.61 | 3.31 |
| 76 | gi\|410979206\|ref\|XP_003995976.1\| | PREDICTED: neutrophil gelatinase-associated lipocalin-like isoform X1 [Felis catus] | 25605 | 13 | 12 | 4 | 4 | 24.7 | 5.46 | 1.1 |
| 77 | gi\|755774158\|ref\|XP_011285490.1\| | PREDICTED: cadherin-related family member 5 [Felis catus] | 84363 | 21 | 18 | 6 | 5 | 8.5 | 4.6 | 0.41 |
| 78 | gi\|410980769\|ref\|XP_003996748.1\| | PREDICTED: collagen alpha-1(I) chain isoform X1 [Felis catus] | 152845 | 14 | 14 | 5 | 5 | 4.1 | 5.75 | 0.17 |
| 79 | gi\|410989433\|ref\|XP_004000966.1\| | PREDICTED: xaa-Pro aminopeptidase 2 [Felis catus] | 82996 | 16 | 14 | 7 | 6 | 8.6 | 6.04 | 0.41 |
| 80 | gi\|755748454\|ref\|XP_004001337.3\| | PREDICTED: keratin, type II cytoskeletal 5 [Felis catus] | 70076 | 25 | 20 | 9 | 7 | 11.9 | 7.59 | 0.61 |
| 81 | gi\|587015109\|ref\|XP_006941697.1\| | PREDICTED: chymotrypsin-like protease CTRL-1 isoform X1 [Felis catus] | 33885 | 18 | 15 | 3 | 3 | 15.7 | 8.97 | 0.52 |
| 82 | gi\|410979342\|ref\|XP_003996044.1\| | PREDICTED: neutrophil gelatinase-associated lipocalin-like [Felis catus] | 34414 | 16 | 12 | 5 | 5 | 11.5 | 5.96 | 1 |
| 83 | gi\|410971077\|ref\|XP_003992000.1\| | PREDICTED: latexin [Felis catus] | 29766 | 16 | 14 | 2 | 2 | 8.6 | 5.83 | 0.38 |
| 84 | gi\|312147372\|ref\|NP_001185858.1\| | protein S100-A12 [Felis catus] | 12584 | 21 | 17 | 3 | 3 | 30.4 | 4.98 | 2.06 |
| 85 | gi\|755772832\|ref\|XP_011285314.1\| | PREDICTED: N-acetylated-alpha-linked acidic dipeptidase-like protein [Felis catus] | 84563 | 19 | 18 | 5 | 5 | 8.2 | 5.44 | 0.33 |
| 86 | gi\|586990665\|ref\|XP_006933138.1\| | PREDICTED: alpha-1-antitrypsin [Felis catus] | 54996 | 16 | 14 | 4 | 4 | 9.9 | 5.62 | 0.42 |
| 87 | gi\|410967853\|ref\|XP_003990428.1\| | PREDICTED: alpha-amylase 2B [Felis catus] | 64202 | 17 | 14 | 6 | 6 | 13.9 | 6.68 | 0.56 |
| 88 | gi\|189303530\|ref\|NP_001121543.1\| | chloride anion exchanger precursor [Felis catus] | 31901 | 10 | 10 | 2 | 2 | 7.9 | 4.8 | 0.35 |
| 89 | gi\|586975121\|ref\|XP_006927896.1\| | PREDICTED: hyaluronan and proteoglycan link protein 1 [Felis catus] | 45753 | 8 | 8 | 1 | 1 | 3.7 | 7.54 | 0.11 |
| 90 | gi\|410952288\|ref\|XP_003982813.1\| | PREDICTED: collagen alpha-2(I) chain [Felis catus] | 141899 | 9 | 9 | 3 | 3 | 3.2 | 9.21 | 0.11 |
| 91 | gi\|755809147\|ref\|XP_003999831.2\| | PREDICTED: protein S100-A8 [Felis catus] | 12891 | 15 | 14 | 4 | 4 | 30.3 | 5.15 | 3.28 |
| 92 | gi\|755796690\|ref\|XP_011288203.1\| | PREDICTED: carcinoembryonic antigen-related cell adhesion molecule 1 isoform X1 [Felis catus] | 61602 | 15 | 14 | 3 | 3 | 6.3 | 5.2 | 0.26 |
| 93 | gi\|755744670\|ref\|XP_011281771.1\| | PREDICTED: alpha-1-antichymotrypsin [Felis catus] | 52251 | 19 | 11 | 6 | 4 | 17.6 | 7.72 | 0.44 |
| 94 | gi\|254750669\|ref\|NP_001157127.1\| | lysosome-associated membrane glycoprotein 1 precursor [Felis catus] | 47997 | 13 | 13 | 1 | 1 | 2.2 | 7.07 | 0.1 |
| 95 | gi\|410962659\|ref\|XP_003987886.1\| | PREDICTED: dihydrolipoyllysine-residue succinyltransferase component of 2-oxoglutarate dehydrogenase complex, mitochondrial [Felis catus] | 55586 | 13 | 11 | 3 | 3 | 7 | 9 | 0.29 |
| 96 | gi\|410964501\|ref\|XP_003988792.1\| | PREDICTED: keratin, type II cytoskeletal 79 [Felis catus] | 64892 | 12 | 9 | 3 | 2 | 4.9 | 8.11 | 0.16 |
| 97 | gi\|523704472\|ref\|NP_001265782.1\| | malate dehydrogenase, mitochondrial [Felis catus] | 41906 | 12 | 10 | 3 | 3 | 9.2 | 8.93 | 0.41 |
| 98 | gi\|146198809\|ref\|NP_001078907.1\| | adiponectin precursor [Felis catus] | 29778 | 12 | 11 | 2 | 2 | 7.8 | 5.68 | 0.38 |
| 99 | gi\|410965016\|ref\|XP_003989048.1\| | PREDICTED: N-acetylglucosamine-6-sulfatase [Felis catus] | 69330 | 13 | 12 | 6 | 5 | 10.5 | 8.4 | 0.41 |
| 100 | gi\|755693865\|ref\|XP_011285140.1\| | PREDICTED: integrin alpha-2 [Felis catus] | 140246 | 10 | 10 | 1 | 1 | 0.9 | 5.24 | 0.03 |
| 101 | gi\|755753175\|ref\|XP_011282860.1\| | PREDICTED: chymotrypsin-like elastase family member 3B [Felis catus] | 33029 | 26 | 21 | 1 | 1 | 3.1 | 5.57 | 0.15 |
| 102 | gi\|224994156\|ref\|NP_001116826.1\| | ubiquitin-60S ribosomal protein L40 precursor [Felis catus] | 19129 | 8 | 7 | 2 | 2 | 19.5 | 9.87 | 0.64 |
| 103 | gi\|410948892\|ref\|XP_003981161.1\| | PREDICTED: creatine kinase S-type, mitochondrial [Felis catus] | 53332 | 18 | 14 | 7 | 6 | 15.8 | 8.61 | 0.71 |
| 104 | gi\|755753697\|ref\|XP_011282920.1\| | PREDICTED: basement membrane-specific heparan sulfate proteoglycan core protein [Felis catus] | 509414 | 12 | 10 | 6 | 5 | 1.6 | 6.09 | 0.05 |
| 105 | gi\|410980935\|ref\|XP_003996829.1\| | PREDICTED: telethonin [Felis catus] | 20680 | 20 | 17 | 3 | 2 | 14.4 | 5.51 | 0.58 |
| 106 | gi\|755807803\|ref\|XP_011289415.1\| | PREDICTED: nicastrin [Felis catus] | 83853 | 8 | 6 | 2 | 2 | 2.8 | 5.59 | 0.12 |
| 107 | gi\|755700303\|ref\|XP_011278526.1\| | PREDICTED: fibulin-2 isoform X1 [Felis catus] | 143033 | 9 | 9 | 1 | 1 | 1.1 | 4.75 | 0.03 |
| 108 | gi\|755748903\|ref\|XP_011282310.1\| | PREDICTED: collagen alpha-1(II) chain [Felis catus] | 161314 | 12 | 6 | 5 | 4 | 4.3 | 8.1 | 0.13 |
| 109 | gi\|410969366\|ref\|XP_003991167.1\| | PREDICTED: fibronectin isoform X6 [Felis catus] | 294420 | 19 | 6 | 6 | 3 | 2.7 | 5.51 | 0.05 |
| 110 | gi\|410947494\|ref\|XP_003980481.1\| | PREDICTED: olfactomedin-4 [Felis catus] | 63056 | 26 | 18 | 5 | 4 | 7.7 | 4.95 | 0.46 |
| 111 | gi\|410957470\|ref\|XP_003985350.1\| | PREDICTED: immunoglobulin J chain [Felis catus] | 20315 | 24 | 24 | 3 | 3 | 16.5 | 4.79 | 1.54 |
| 112 | gi\|587017024\|ref\|XP_006942402.1\| | PREDICTED: 4-aminobutyrate aminotransferase, mitochondrial [Felis catus] | 64599 | 8 | 6 | 4 | 2 | 8.2 | 8.69 | 0.16 |
| 113 | gi\|755792933\|ref\|XP_011287749.1\| | PREDICTED: ectonucleotide pyrophosphatase/phosphodiesterase family member 7 [Felis catus] | 74030 | 11 | 9 | 6 | 6 | 10.8 | 8.54 | 0.47 |
| 114 | gi\|410964647\|ref\|XP_003988864.1\| | PREDICTED: keratin, type II cytoskeletal 2 oral [Felis catus] | 73528 | 16 | 10 | 6 | 4 | 7.6 | 8.38 | 0.3 |
| 115 | gi\|410951335\|ref\|XP_003982353.1\| | PREDICTED: troponin C, slow skeletal and cardiac muscles [Felis catus] | 21699 | 9 | 9 | 2 | 2 | 13 | 4.04 | 0.93 |
| 116 | gi\|755753040\|ref\|XP_003989706.2\| | PREDICTED: tissue alpha-L-fucosidase [Felis catus] | 60069 | 11 | 9 | 4 | 4 | 10.2 | 6.2 | 0.37 |
| 117 | gi\|755732164\|ref\|XP_011280961.1\| | PREDICTED: superoxide dismutase [Mn], mitochondrial [Felis catus] | 50503 | 11 | 8 | 5 | 2 | 10.8 | 9.35 | 0.21 |
| 118 | gi\|170763531\|ref\|NP_001116218.2\| | myeloperoxidase precursor [Felis catus] | 86981 | 7 | 7 | 2 | 2 | 2.9 | 9.11 | 0.12 |
| 119 | gi\|755743575\|ref\|XP_003987862.2\| | PREDICTED: prostaglandin reductase 2 [Felis catus] | 43262 | 7 | 7 | 1 | 1 | 2.8 | 5.57 | 0.12 |
| 120 | gi\|755801251\|ref\|XP_006942027.2\| | PREDICTED: zinc-alpha-2-glycoprotein [Felis catus] | 36679 | 11 | 6 | 4 | 3 | 17.8 | 4.93 | 0.48 |
| 121 | gi\|755779082\|ref\|XP_011286069.1\| | PREDICTED: sushi domain-containing protein 2 [Felis catus] | 96191 | 9 | 5 | 3 | 2 | 4.3 | 5.82 | 0.1 |
| 122 | gi\|410956528\|ref\|XP_003984894.1\| | PREDICTED: cathepsin B [Felis catus] | 42177 | 11 | 9 | 2 | 2 | 5.3 | 5.45 | 0.25 |
| 123 | gi\|410982852\|ref\|XP_003997760.1\| | PREDICTED: persulfide dioxygenase ETHE1, mitochondrial [Felis catus] | 27091 | 7 | 6 | 2 | 2 | 10.1 | 5.77 | 0.42 |
| 124 | gi\|410958194\|ref\|XP_003985704.1\| | PREDICTED: glutathione peroxidase 6 isoform X1 [Felis catus] | 28927 | 8 | 7 | 3 | 3 | 18.1 | 8.19 | 0.64 |
| 125 | gi\|410983998\|ref\|XP_003998322.1\| | PREDICTED: chymotrypsinogen B [Felis catus] | 27424 | 14 | 12 | 2 | 2 | 7.5 | 7.63 | 0.68 |
| 126 | gi\|410977275\|ref\|XP_003995033.1\| | PREDICTED: macrophage migration inhibitory factor [Felis catus] | 13510 | 8 | 8 | 1 | 1 | 9.6 | 7.74 | 0.41 |
| 127 | gi\|410953638\|ref\|XP_003983477.1\| | PREDICTED: WAP four-disulfide core domain protein 2 isoform X2 [Felis catus] | 15669 | 5 | 5 | 1 | 1 | 8.1 | 5.42 | 0.35 |
| 128 | gi\|587014614\|ref\|XP_006941518.1\| | PREDICTED: xaa-Pro dipeptidase [Felis catus] | 62995 | 12 | 10 | 7 | 6 | 12.5 | 5.89 | 0.58 |
| 129 | gi\|194353917\|ref\|NP_001123862.1\| | trefoil factor 3 precursor [Felis catus] | 9880 | 4 | 4 | 1 | 1 | 20 | 4.32 | 0.6 |
| 130 | gi\|587015573\|ref\|XP_006941863.1\| | PREDICTED: N-acetylgalactosamine-6-sulfatase [Felis catus] | 57777 | 9 | 9 | 3 | 3 | 4.6 | 6.36 | 0.28 |
| 131 | gi\|755765573\|ref\|XP_006936377.2\| | PREDICTED: neprilysin [Felis catus] | 98576 | 8 | 6 | 5 | 3 | 5.2 | 5.48 | 0.16 |
| 132 | gi\|755805026\|ref\|XP_011289070.1\| | PREDICTED: serine protease 27-like [Felis catus] | 37262 | 12 | 12 | 3 | 3 | 7.2 | 4.89 | 0.67 |
| 133 | gi\|755757840\|ref\|XP_011283428.1\| | PREDICTED: cathepsin S isoform X1 [Felis catus] | 44950 | 8 | 5 | 4 | 4 | 10.8 | 7.52 | 0.53 |
| 134 | gi\|755758826\|ref\|XP_011283568.1\| | PREDICTED: nebulin isoform X1 [Felis catus] | 1084405 | 12 | 6 | 1 | 1 | 0.1 | 9.09 | 0 |
| 135 | gi\|410955202\|ref\|XP_003984246.1\| | PREDICTED: lithostathine-like [Felis catus] | 22063 | 7 | 6 | 2 | 2 | 13.6 | 6.93 | 1.36 |
| 136 | gi\|410969050\|ref\|XP_003991010.1\| | PREDICTED: collagen alpha-1(III) chain [Felis catus] | 153840 | 5 | 4 | 1 | 1 | 0.8 | 6.22 | 0.03 |
| 137 | gi\|755809151\|ref\|XP_003999832.2\| | PREDICTED: protein S100-A9 [Felis catus] | 17685 | 11 | 5 | 4 | 2 | 28.9 | 5.87 | 1.23 |
| 138 | gi\|755807297\|ref\|XP_011289352.1\| | PREDICTED: laminin subunit gamma-1, partial [Felis catus] | 198018 | 5 | 4 | 2 | 2 | 2 | 5.03 | 0.05 |
| 139 | gi\|410962651\|ref\|XP_003987882.1\| | PREDICTED: epididymal secretory protein E1 [Felis catus] | 19409 | 6 | 3 | 3 | 2 | 18.8 | 8.52 | 0.63 |
| 140 | gi\|410959962\|ref\|XP_003986567.1\| | PREDICTED: acid sphingomyelinase-like phosphodiesterase 3a [Felis catus] | 57229 | 6 | 3 | 2 | 1 | 4.4 | 5.78 | 0.09 |
| 141 | gi\|410965904\|ref\|XP_003989478.1\| | PREDICTED: arylsulfatase A isoform X1 [Felis catus] | 56214 | 6 | 6 | 2 | 2 | 4.7 | 5.3 | 0.19 |
| 142 | gi\|586980052\|ref\|XP_003983266.2\| | PREDICTED: amiloride-sensitive amine oxidase [copper-containing] [Felis catus] | 101743 | 11 | 9 | 2 | 2 | 2.1 | 7.71 | 0.1 |
| 143 | gi\|410951463\|ref\|XP_003982416.1\| | PREDICTED: filamin-B isoform X1 [Felis catus] | 319897 | 11 | 8 | 3 | 3 | 0.9 | 5.42 | 0.05 |
| 144 | gi\|755773699\|ref\|XP_011285433.1\| | PREDICTED: glutathione S-transferase P [Felis catus] | 26861 | 2 | 2 | 1 | 1 | 7.6 | 8.34 | 0.19 |
| 145 | gi\|586991840\|ref\|XP_006933559.1\| | PREDICTED: alpha-2-macroglobulin [Felis catus] | 185650 | 10 | 4 | 2 | 2 | 1.3 | 5.81 | 0.05 |
| 146 | gi\|755696144\|ref\|XP_011287788.1\| | PREDICTED: resistin [Felis catus] | 16746 | 7 | 6 | 1 | 1 | 4.8 | 9.05 | 0.32 |
| 147 | gi\|410971867\|ref\|XP_003992383.1\| | PREDICTED: radixin isoform X2 [Felis catus] | 86998 | 11 | 10 | 2 | 1 | 2.5 | 6.3 | 0.06 |
| 148 | gi\|410969762\|ref\|XP_003991361.1\| | PREDICTED: THAP domain-containing protein 4 isoform X2 [Felis catus] | 20752 | 7 | 4 | 2 | 2 | 8.5 | 6.22 | 0.58 |
| 149 | gi\|755741361\|ref\|XP_011281388.1\| | PREDICTED: LOW QUALITY PROTEIN: formin-1 [Felis catus] | 157800 | 7 | 7 | 1 | 1 | 0.7 | 5.55 | 0.03 |
| 150 | gi\|410972925\|ref\|XP_003992906.1\| | PREDICTED: hemoglobin subunit beta-A/B [Felis catus] | 19159 | 5 | 4 | 2 | 2 | 15 | 7.1 | 0.64 |
| 151 | gi\|410950888\|ref\|XP_003982134.1\| | PREDICTED: cartilage oligomeric matrix protein [Felis catus] | 90189 | 3 | 3 | 2 | 2 | 2.5 | 4.36 | 0.11 |
| 152 | gi\|587018338\|ref\|XP_006942888.1\| | PREDICTED: C4b-binding protein alpha chain [Felis catus] | 79360 | 5 | 4 | 1 | 1 | 2 | 7.58 | 0.06 |
| 153 | gi\|755762615\|ref\|XP_011284119.1\| | PREDICTED: enteropeptidase-like, partial [Felis catus] | 49472 | 4 | 4 | 1 | 1 | 3 | 4.51 | 0.1 |
| 154 | gi\|755808188\|ref\|XP_011289446.1\| | PREDICTED: bifunctional glutamate/proline--tRNA ligase [Felis catus] | 205155 | 14 | 11 | 2 | 2 | 1.1 | 7.31 | 0.05 |
| 155 | gi\|586993378\|ref\|XP_006934051.1\| | PREDICTED: thioredoxin reductase 1, cytoplasmic [Felis catus] | 63054 | 4 | 4 | 2 | 2 | 4 | 6.07 | 0.16 |
| 156 | gi\|586989523\|ref\|XP_006932772.1\| | PREDICTED: ras-related protein Rab-2B isoform X2 [Felis catus] | 21218 | 15 | 0 | 1 | 0 | 3.5 | 6.43 |  |
| 157 | gi\|755773904\|ref\|XP_011285461.1\| | PREDICTED: mucin-5B [Felis catus] | 413889 | 5 | 5 | 1 | 1 | 0.3 | 5.54 | 0.01 |
| 158 | gi\|755765419\|ref\|XP_011284468.1\| | PREDICTED: ceruloplasmin [Felis catus] | 141290 | 5 | 2 | 5 | 2 | 6.4 | 5.41 | 0.07 |
| 159 | gi\|410965747\|ref\|XP_003989403.1\| | PREDICTED: polymerase delta-interacting protein 3 [Felis catus] | 55293 | 7 | 7 | 1 | 1 | 1.4 | 10 | 0.09 |
| 160 | gi\|755784671\|ref\|XP_011286776.1\| | PREDICTED: tenascin isoform X1 [Felis catus] | 263598 | 4 | 3 | 1 | 1 | 0.6 | 4.93 | 0.02 |
| 161 | gi\|755764004\|ref\|XP_011284298.1\| | PREDICTED: mucin-13 [Felis catus] | 66068 | 3 | 3 | 2 | 2 | 3.7 | 5.79 | 0.16 |
| 162 | gi\|586983843\|ref\|XP_006930873.1\| | PREDICTED: fibrinogen alpha chain [Felis catus] | 78480 | 5 | 2 | 2 | 1 | 2.8 | 6.64 | 0.06 |
| 163 | gi\|587011197\|ref\|XP_006940265.1\| | PREDICTED: nucleoside diphosphate kinase A [Felis catus] | 19979 | 4 | 3 | 1 | 1 | 11.2 | 5.78 | 0.27 |
| 164 | gi\|755704144\|ref\|XP_011278891.1\| | PREDICTED: probable maltase-glucoamylase-like protein [Felis catus] | 311591 | 7 | 4 | 4 | 2 | 1.7 | 5.27 | 0.03 |
| 165 | gi\|586979805\|ref\|XP_006929495.1\| | PREDICTED: serine protease 58-like [Felis catus] | 29977 | 8 | 7 | 1 | 1 | 3 | 6.28 | 0.17 |
| 166 | gi\|755750150\|ref\|XP_011282463.1\| | PREDICTED: leukotriene A-4 hydrolase [Felis catus] | 79241 | 4 | 4 | 2 | 2 | 3.4 | 6.09 | 0.13 |
| 167 | gi\|586998746\|ref\|XP_006935932.1\| | PREDICTED: cystatin-B [Felis catus] | 11187 | 2 | 2 | 1 | 1 | 15.2 | 6.9 | 0.52 |
| 168 | gi\|586998950\|ref\|XP_006936005.1\| | PREDICTED: amyloid beta A4 protein isoform X1 [Felis catus] | 94096 | 2 | 2 | 1 | 1 | 1.8 | 4.74 | 0.05 |
| 169 | gi\|410950750\|ref\|XP_003982066.1\| | PREDICTED: tropomyosin alpha-4 chain isoform X1 [Felis catus] | 42069 | 2 | 2 | 1 | 1 | 3.5 | 4.71 | 0.12 |
| 170 | gi\|755786511\|ref\|XP_006944370.2\| | PREDICTED: dipeptidyl peptidase 2 [Felis catus] | 60734 | 5 | 2 | 1 | 1 | 1.7 | 6.18 | 0.08 |
| 171 | gi\|586999703\|ref\|XP_006936266.1\| | PREDICTED: alpha-2-HS-glycoprotein [Felis catus] | 42906 | 4 | 4 | 1 | 1 | 2.2 | 5.11 | 0.12 |
| 172 | gi\|755822903\|ref\|XP_011278187.1\| | PREDICTED: immunoglobulin omega chain-like, partial [Felis catus] | 15334 | 5 | 1 | 2 | 1 | 12.2 | 6.37 | 0.36 |
| 173 | gi\|586998890\|ref\|XP_006935984.1\| | PREDICTED: superoxide dismutase [Cu-Zn] [Felis catus] | 18488 | 4 | 2 | 2 | 1 | 11.8 | 6.28 | 0.29 |
| 174 | gi\|587012447\|ref\|XP_006940691.1\| | PREDICTED: envoplakin [Felis catus] | 253078 | 11 | 6 | 1 | 1 | 0.4 | 6.24 | 0.02 |
| 175 | gi\|410969052\|ref\|XP_003991011.1\| | PREDICTED: collagen alpha-2(V) chain [Felis catus] | 160507 | 6 | 2 | 2 | 2 | 1.3 | 6.33 | 0.06 |
| 176 | gi\|755688023\|ref\|XP_011286991.1\| | PREDICTED: LOW QUALITY PROTEIN: spermatid-associated protein-like [Felis catus] | 39036 | 6 | 4 | 2 | 1 | 5.3 | 8.66 | 0.13 |
| 177 | gi\|586978791\|ref\|XP_006929168.1\| | PREDICTED: neuronal cell adhesion molecule isoform X15 [Felis catus] | 147792 | 4 | 4 | 1 | 1 | 0.7 | 5.51 | 0.03 |
| 178 | gi\|410968892\|ref\|XP_003990933.1\| | PREDICTED: ATP synthase F(0) complex subunit C3, mitochondrial [Felis catus] | 15796 | 4 | 2 | 1 | 1 | 5 | 9.56 | 0.35 |
| 179 | gi\|114326408\|ref\|NP_001041616.1\| | ferritin heavy chain [Felis catus] | 24620 | 5 | 2 | 3 | 2 | 13.1 | 5.53 | 0.47 |
| 180 | gi\|410977086\|ref\|XP_003994942.1\| | PREDICTED: immunoglobulin omega chain-like [Felis catus] | 18001 | 2 | 2 | 1 | 1 | 6.5 | 6.41 | 0.3 |
| 181 | gi\|312147375\|ref\|NP_001185857.1\| | haptoglobin precursor [Felis catus] | 45282 | 2 | 2 | 2 | 2 | 6.3 | 5.55 | 0.23 |
| 182 | gi\|410959353\|ref\|XP_003986275.1\| | PREDICTED: phosphoglycerate kinase 2 [Felis catus] | 54837 | 5 | 3 | 2 | 1 | 3.1 | 8.02 | 0.09 |
| 183 | gi\|755781491\|ref\|XP_011286374.1\| | PREDICTED: laminin subunit alpha-3 [Felis catus] | 388892 | 4 | 0 | 1 | 0 | 0.3 | 6.84 |  |
| 184 | gi\|755773471\|ref\|XP_011285391.1\| | PREDICTED: cysteine--tRNA ligase, cytoplasmic isoform X1 [Felis catus] | 109011 | 3 | 3 | 1 | 1 | 1 | 6.9 | 0.04 |
| 185 | gi\|410974061\|ref\|XP_003993466.1\| | PREDICTED: gastric intrinsic factor [Felis catus] | 50284 | 3 | 3 | 2 | 2 | 4.1 | 6.55 | 0.21 |
| 186 | gi\|410947812\|ref\|XP_003980636.1\| | PREDICTED: olfactory receptor 2A12-like [Felis catus] | 38613 | 2 | 2 | 1 | 1 | 2.9 | 8.47 | 0.13 |
| 187 | gi\|410960930\|ref\|XP_003987040.1\| | PREDICTED: uveal autoantigen with coiled-coil domains and ankyrin repeats isoform X1 [Felis catus] | 198164 | 9 | 4 | 3 | 2 | 1.5 | 6.33 | 0.05 |
| 188 | gi\|325652162\|ref\|NP_001191706.1\| | Fel d 7 allergen precursor [Felis catus] | 24010 | 1 | 1 | 1 | 1 | 7.2 | 4.87 | 0.22 |
| 189 | gi\|755743012\|ref\|XP_011281562.1\| | PREDICTED: nesprin-2 isoform X3 [Felis catus] | 939058 | 7 | 7 | 2 | 2 | 0.2 | 5.2 | 0.01 |
| 190 | gi\|410964479\|ref\|XP_003988782.1\| | PREDICTED: keratin, type II cuticular Hb5-like [Felis catus] | 62447 | 10 | 1 | 3 | 1 | 5.3 | 5.77 | 0.08 |
| 191 | gi\|523704480\|ref\|NP_001265786.1\| | keratin, type II cytoskeletal 74 [Felis catus] | 66590 | 7 | 3 | 3 | 1 | 3.6 | 8.54 | 0.07 |
| 192 | gi\|587009145\|ref\|XP_006939520.1\| | PREDICTED: 78 kDa glucose-regulated protein [Felis catus] | 86531 | 7 | 2 | 2 | 1 | 2 | 5.06 | 0.06 |
| 193 | gi\|755810241\|ref\|XP_011289700.1\| | PREDICTED: cadherin-17 [Felis catus] | 102941 | 12 | 9 | 3 | 3 | 2.3 | 4.88 | 0.15 |
| 194 | gi\|410987534\|ref\|XP_004000054.1\| | PREDICTED: ribonuclease UK114 [Felis catus] | 16243 | 1 | 1 | 1 | 1 | 8 | 7.82 | 0.34 |
| 195 | gi\|755790091\|ref\|XP_003996986.2\| | PREDICTED: keratin, type I cytoskeletal 9 [Felis catus] | 73697 | 5 | 4 | 2 | 1 | 1.9 | 5.32 | 0.07 |
| 196 | gi\|410987068\|ref\|XP_003999830.1\| | PREDICTED: protein S100-A6 [Felis catus] | 12682 | 4 | 3 | 3 | 2 | 22.2 | 5.06 | 1.09 |
| 197 | gi\|755747220\|ref\|XP_011282102.1\| | PREDICTED: ovostatin homolog 2-like [Felis catus] | 179597 | 5 | 3 | 2 | 1 | 1.3 | 5.25 | 0.03 |
| 198 | gi\|586983932\|ref\|XP_006930903.1\| | PREDICTED: methylmalonic aciduria type A protein, mitochondrial isoform X1 [Felis catus] | 54766 | 3 | 1 | 1 | 1 | 2.6 | 9.51 | 0.09 |
| 199 | gi\|755756032\|ref\|XP_011283231.1\| | PREDICTED: medium-chain specific acyl-CoA dehydrogenase, mitochondrial [Felis catus] | 54121 | 3 | 2 | 1 | 1 | 1.7 | 8.42 | 0.09 |
| 200 | gi\|755797412\|ref\|XP_011288287.1\| | PREDICTED: LOW QUALITY PROTEIN: glucose-6-phosphate isomerase [Felis catus] | 71519 | 3 | 1 | 2 | 1 | 3 | 7.75 | 0.07 |
| 201 | gi\|410948265\|ref\|XP_003980861.1\| | PREDICTED: stress-70 protein, mitochondrial [Felis catus] | 86251 | 4 | 0 | 1 | 0 | 1 | 5.91 |  |
| 202 | gi\|410980881\|ref\|XP_003996802.1\| | PREDICTED: CDK5 regulatory subunit-associated protein 3 [Felis catus] | 63642 | 18 | 10 | 1 | 1 | 1.2 | 4.79 | 0.08 |
| 203 | gi\|755792792\|ref\|XP_006940713.2\| | PREDICTED: lysosomal alpha-glucosidase isoform X1 [Felis catus] | 111034 | 3 | 2 | 3 | 2 | 4.5 | 6.2 | 0.09 |
| 204 | gi\|410966581\|ref\|XP_003989809.1\| | PREDICTED: acid sphingomyelinase-like phosphodiesterase 3b [Felis catus] | 55144 | 1 | 1 | 1 | 1 | 3.5 | 6.02 | 0.09 |
| 205 | gi\|410985002\|ref\|XP_003998814.1\| | PREDICTED: probable glutamate--tRNA ligase, mitochondrial [Felis catus] | 63045 | 3 | 3 | 1 | 1 | 1.1 | 8.82 | 0.08 |
| 206 | gi\|586974191\|ref\|XP_006927587.1\| | PREDICTED: collagen alpha-1(XXIII) chain [Felis catus] | 47869 | 1 | 1 | 1 | 1 | 2.9 | 6.87 | 0.1 |
| 207 | gi\|755721900\|ref\|XP_011279961.1\| | PREDICTED: ankyrin-2 isoform X1 [Felis catus] | 504662 | 5 | 1 | 1 | 1 | 0.2 | 4.99 | 0.01 |
| 208 | gi\|755739175\|ref\|XP_011281147.1\| | PREDICTED: dual oxidase 2 [Felis catus] | 187771 | 3 | 3 | 1 | 1 | 0.4 | 7.42 | 0.03 |
| 209 | gi\|755699181\|ref\|XP_006928695.2\| | PREDICTED: gamma-interferon-inducible lysosomal thiol reductase [Felis catus] | 35333 | 1 | 1 | 1 | 1 | 3.8 | 5.57 | 0.14 |
| 210 | gi\|410971725\|ref\|XP_003992315.1\| | PREDICTED: EPM2A-interacting protein 1 [Felis catus] | 77378 | 5 | 3 | 2 | 2 | 2 | 6.14 | 0.13 |
| 211 | gi\|410969292\|ref\|XP_003991130.1\| | PREDICTED: gamma-crystallin B [Felis catus] | 22250 | 2 | 2 | 1 | 1 | 9.1 | 7.55 | 0.24 |
| 212 | gi\|410985038\|ref\|XP_003998832.1\| | PREDICTED: putative RNA exonuclease NEF-sp [Felis catus] | 103199 | 4 | 4 | 1 | 1 | 0.8 | 9.31 | 0.05 |
| 213 | gi\|755773900\|ref\|XP_011285460.1\| | PREDICTED: LOW QUALITY PROTEIN: mucin-5AC [Felis catus] | 359307 | 2 | 2 | 1 | 1 | 0.3 | 6.3 | 0.01 |
| 214 | gi\|410977708\|ref\|XP_003995243.1\| | PREDICTED: spindle and kinetochore-associated protein 1 [Felis catus] | 35677 | 4 | 2 | 1 | 1 | 2.7 | 6.76 | 0.14 |
| 215 | gi\|755756174\|ref\|XP_011283244.1\| | PREDICTED: uncharacterized protein C1orf141 homolog [Felis catus] | 65482 | 4 | 4 | 1 | 1 | 1.8 | 9.81 | 0.08 |
| 216 | gi\|755707146\|ref\|XP_011279140.1\| | PREDICTED: LOW QUALITY PROTEIN: helicase with zinc finger domain 2 [Felis catus] | 338182 | 3 | 3 | 1 | 1 | 0.2 | 8.57 | 0.01 |
| 217 | gi\|755691216\|ref\|XP_011282008.1\| | PREDICTED: LOW QUALITY PROTEIN: importin-11-like [Felis catus] | 99451 | 3 | 3 | 1 | 1 | 0.8 | 5.36 | 0.05 |
| 218 | gi\|755730554\|ref\|XP_011280798.1\| | PREDICTED: LOW QUALITY PROTEIN: midasin [Felis catus] | 710516 | 10 | 9 | 1 | 1 | 0.1 | 5.47 | 0.01 |
| 219 | gi\|587016752\|ref\|XP_006942304.1\| | PREDICTED: LOW QUALITY PROTEIN: dynein heavy chain 3, axonemal [Felis catus] | 540663 | 6 | 0 | 2 | 0 | 0.4 | 5.61 |  |
| 220 | gi\|57619018\|ref\|NP_001009848.1\| | sodium/calcium exchanger 1 precursor [Felis catus] | 120306 | 2 | 1 | 1 | 1 | 2.9 | 4.91 | 0.04 |
| 221 | gi\|755702727\|ref\|XP_011278766.1\| | PREDICTED: mammalian ependymin-related protein 1 [Felis catus] | 37604 | 7 | 5 | 1 | 1 | 2.2 | 9.24 | 0.13 |
| 222 | gi\|755764780\|ref\|XP_003991995.2\| | PREDICTED: structural maintenance of chromosomes protein 4 [Felis catus] | 181799 | 4 | 3 | 1 | 1 | 0.6 | 6.7 | 0.03 |
| 223 | gi\|410970314\|ref\|XP_003991630.1\| | PREDICTED: omega-amidase NIT2 [Felis catus] | 35387 | 2 | 1 | 2 | 1 | 6 | 5.88 | 0.14 |
| 224 | gi\|586984721\|ref\|XP_006931157.1\| | PREDICTED: alpha-S2-casein-like [Felis catus] | 31833 | 3 | 3 | 1 | 1 | 3.8 | 5.73 | 0.16 |
| 225 | gi\|755765659\|ref\|XP_011284504.1\| | PREDICTED: ceruloplasmin-like [Felis catus] | 134644 | 2 | 2 | 2 | 2 | 1.6 | 5.96 | 0.07 |
| 226 | gi\|755760654\|ref\|XP_006935585.2\| | PREDICTED: frizzled-5, partial [Felis catus] | 70452 | 4 | 4 | 1 | 1 | 1.1 | 8.35 | 0.07 |
| 227 | gi\|755803944\|ref\|XP_011288956.1\| | PREDICTED: deoxyribonuclease-1 [Felis catus] | 34203 | 3 | 2 | 2 | 1 | 5.2 | 5.23 | 0.15 |
| 228 | gi\|755763912\|ref\|XP_011284288.1\| | PREDICTED: ephrin type-A receptor 6 [Felis catus] | 139721 | 3 | 0 | 1 | 0 | 0.6 | 6.25 |  |
| 229 | gi\|755691610\|ref\|XP_011282449.1\| | PREDICTED: corticotropin-releasing factor-binding protein [Felis catus] | 39265 | 6 | 5 | 1 | 1 | 2.2 | 6.59 | 0.13 |
| 230 | gi\|410966512\|ref\|XP_003989776.1\| | PREDICTED: myomesin-3 [Felis catus] | 186147 | 3 | 2 | 2 | 1 | 0.8 | 5.98 | 0.03 |
| 231 | gi\|410972013\|ref\|XP_003992455.1\| | PREDICTED: myelin protein zero-like protein 2 [Felis catus] | 27779 | 1 | 1 | 1 | 1 | 6 | 6.9 | 0.19 |
| 232 | gi\|586982015\|ref\|XP_006930258.1\| | PREDICTED: dynactin subunit 1 isoform X2 [Felis catus] | 159781 | 3 | 2 | 2 | 1 | 1.2 | 5.37 | 0.03 |
| 233 | gi\|410982762\|ref\|XP_003997717.1\| | PREDICTED: peptidoglycan recognition protein 1 isoform X1 [Felis catus] | 23670 | 1 | 1 | 1 | 1 | 6.6 | 8.13 | 0.22 |
| 234 | gi\|587010956\|ref\|XP_006940177.1\| | PREDICTED: acetyl-CoA carboxylase 1 isoform X3 [Felis catus] | 292667 | 4 | 4 | 1 | 1 | 0.3 | 6.01 | 0.02 |
| 235 | gi\|587019161\|ref\|XP_006943179.1\| | PREDICTED: NAD(P)H-hydrate epimerase [Felis catus] | 32295 | 3 | 0 | 1 | 0 | 3 | 7.62 |  |
| 236 | gi\|410974554\|ref\|XP_003993709.1\| | PREDICTED: kinesin light chain 2 [Felis catus] | 78983 | 9 | 8 | 1 | 1 | 1 | 6.58 | 0.06 |
| 237 | gi\|755699397\|ref\|XP_006928978.2\| | PREDICTED: THO complex subunit 7 homolog [Felis catus] | 28767 | 2 | 2 | 1 | 1 | 2.9 | 5.57 | 0.18 |
| 238 | gi\|755732199\|ref\|XP_011280966.1\| | PREDICTED: LOW QUALITY PROTEIN: nesprin-1 [Felis catus] | 1172331 | 14 | 3 | 5 | 2 | 0.4 | 5.38 | 0.01 |
| 239 | gi\|755765599\|ref\|XP_011284492.1\| | PREDICTED: serotransferrin [Felis catus] | 133794 | 3 | 2 | 2 | 1 | 1.4 | 8.41 | 0.04 |
| 240 | gi\|410970549\|ref\|XP_003991741.1\| | PREDICTED: coiled-coil domain-containing protein 58 isoform X1 [Felis catus] | 20127 | 2 | 2 | 1 | 1 | 6.2 | 6.91 | 0.27 |
| 241 | gi\|587013544\|ref\|XP_006941112.1\| | PREDICTED: carcinoembryonic antigen-related cell adhesion molecule 18 [Felis catus] | 47989 | 2 | 2 | 2 | 2 | 2 | 8.78 | 0.22 |
| 242 | gi\|410956071\|ref\|XP_003984668.1\| | PREDICTED: microtubule-associated tumor suppressor 1 isoform X4 [Felis catus] | 62808 | 7 | 5 | 2 | 1 | 2.5 | 7.62 | 0.08 |
| 243 | gi\|755784889\|ref\|XP_011286804.1\| | PREDICTED: thioredoxin [Felis catus] | 15085 | 2 | 1 | 1 | 1 | 8.6 | 4.97 | 0.37 |
| 244 | gi\|410952743\|ref\|XP_003983038.1\| | PREDICTED: ankyrin repeat and SOCS box protein 15 isoform X1 [Felis catus] | 73913 | 10 | 7 | 1 | 1 | 1 | 5.37 | 0.07 |
| 245 | gi\|755741661\|ref\|XP_011281421.1\| | PREDICTED: chromodomain-helicase-DNA-binding protein 8 isoform X1 [Felis catus] | 332396 | 7 | 3 | 3 | 1 | 0.7 | 6.03 | 0.01 |
| 246 | gi\|410987405\|ref\|XP_003999995.1\| | PREDICTED: carbonic anhydrase 2 [Felis catus] | 34608 | 2 | 1 | 2 | 1 | 10.4 | 6.87 | 0.15 |
| 247 | gi\|410960411\|ref\|XP_003986783.1\| | PREDICTED: hydroxylysine kinase [Felis catus] | 47123 | 3 | 1 | 1 | 1 | 1.9 | 5.91 | 0.11 |
| 248 | gi\|755810848\|ref\|XP_004000158.2\| | PREDICTED: LOW QUALITY PROTEIN: fer-1-like protein 6 [Felis catus] | 247630 | 2 | 2 | 1 | 1 | 0.4 | 6.02 | 0.02 |
| 249 | gi\|755795983\|ref\|XP_011288130.1\| | PREDICTED: sodium/calcium exchanger 2 [Felis catus] | 88617 | 3 | 2 | 1 | 1 | 0.8 | 5.39 | 0.06 |
| 250 | gi\|587013338\|ref\|XP_006941033.1\| | PREDICTED: nuclear pore glycoprotein p62 [Felis catus] | 58079 | 5 | 3 | 1 | 1 | 1.3 | 5.29 | 0.09 |
| 251 | gi\|410972493\|ref\|XP_003992693.1\| | PREDICTED: dipeptidyl peptidase 1 isoform X1 [Felis catus] | 57337 | 2 | 2 | 1 | 1 | 1.3 | 6.02 | 0.09 |
| 252 | gi\|586983143\|ref\|XP_006930646.1\| | PREDICTED: protocadherin Fat 1 [Felis catus] | 557891 | 2 | 2 | 1 | 1 | 0.2 | 4.85 | 0.01 |
| 253 | gi\|410971206\|ref\|XP_003992063.1\| | PREDICTED: E3 ubiquitin-protein ligase RNF13 [Felis catus] | 48778 | 2 | 2 | 1 | 1 | 1.8 | 4.9 | 0.1 |
| 254 | gi\|755731728\|ref\|XP_011280915.1\| | PREDICTED: laminin subunit alpha-2 [Felis catus] | 394629 | 14 | 4 | 2 | 1 | 0.4 | 5.92 | 0.01 |
| 255 | gi\|586973159\|ref\|XP_006927228.1\| | PREDICTED: sacsin isoform X2 [Felis catus] | 585898 | 4 | 1 | 2 | 1 | 0.3 | 6.51 | 0.01 |
| 256 | gi\|755693500\|ref\|XP_011284685.1\| | PREDICTED: maestro heat-like repeat-containing protein family member 2B [Felis catus] | 204480 | 7 | 7 | 1 | 1 | 0.5 | 5.88 | 0.02 |
| 257 | gi\|410948204\|ref\|XP_003980831.1\| | PREDICTED: transforming growth factor-beta-induced protein ig-h3 [Felis catus] | 83461 | 2 | 2 | 1 | 1 | 1 | 6.79 | 0.06 |
| 258 | gi\|410981598\|ref\|XP_003997154.1\| | PREDICTED: ATP-binding cassette sub-family A member 9 [Felis catus] | 207492 | 11 | 8 | 1 | 1 | 0.4 | 7.18 | 0.02 |
| 259 | gi\|755788539\|ref\|XP_006940115.2\| | PREDICTED: oligodendrocyte-myelin glycoprotein [Felis catus] | 55547 | 3 | 0 | 1 | 0 | 2 | 8.57 |  |
| 260 | gi\|112807238\|ref\|NP_001036805.1\| | phosducin [Felis catus] | 33214 | 3 | 1 | 1 | 1 | 2.4 | 4.87 | 0.15 |
| 261 | gi\|589811507\|ref\|NP_001277178.1\| | retinol-binding protein 4 precursor [Felis catus] | 25975 | 1 | 1 | 1 | 1 | 5.5 | 5.24 | 0.2 |
| 262 | gi\|755757349\|ref\|XP_011283383.1\| | PREDICTED: cold shock domain-containing protein E1 isoform X1 [Felis catus] | 109170 | 3 | 3 | 1 | 1 | 0.7 | 6.1 | 0.04 |
| 263 | gi\|410985421\|ref\|XP_003999021.1\| | PREDICTED: hemoglobin subunit alpha [Felis catus] | 18176 | 2 | 0 | 1 | 0 | 6.3 | 7.77 |  |
| 264 | gi\|755806910\|ref\|XP_011289285.1\| | PREDICTED: ribosomal protein S6 kinase delta-1 isoform X1 [Felis catus] | 114761 | 6 | 3 | 1 | 1 | 0.8 | 4.85 | 0.04 |
| 265 | gi\|586976566\|ref\|XP_006928393.1\| | PREDICTED: cdc42-interacting protein 4 [Felis catus] | 101060 | 3 | 1 | 1 | 1 | 1.3 | 6.7 | 0.05 |
| 266 | gi\|182509186\|ref\|NP_001116804.1\| | sucrase-isomaltase, intestinal [Felis catus] | 231560 | 2 | 1 | 2 | 1 | 0.8 | 5.7 | 0.02 |
| 267 | gi\|410983563\|ref\|XP_003998108.1\| | PREDICTED: solute carrier family 12 member 3 [Felis catus] | 124501 | 2 | 1 | 1 | 1 | 0.8 | 7.54 | 0.04 |
| 268 | gi\|410957053\|ref\|XP_003985149.1\| | PREDICTED: hydroxyacyl-coenzyme A dehydrogenase, mitochondrial [Felis catus] | 41733 | 1 | 1 | 1 | 1 | 2.5 | 9.02 | 0.12 |
| 269 | gi\|587018157\|ref\|XP_006942828.1\| | PREDICTED: centrosome-associated protein 350 isoform X1 [Felis catus] | 415218 | 3 | 2 | 2 | 1 | 0.4 | 6.14 | 0.01 |
| 270 | gi\|410957776\|ref\|XP_003985500.1\| | PREDICTED: magnesium transporter NIPA3 [Felis catus] | 50926 | 1 | 1 | 1 | 1 | 1.7 | 6.66 | 0.1 |
| 271 | gi\|755722232\|ref\|XP_011279991.1\| | PREDICTED: nocturnin [Felis catus] | 45805 | 3 | 0 | 1 | 0 | 2.2 | 5.63 |  |
| 272 | gi\|410965082\|ref\|XP_003989081.1\| | PREDICTED: lysozyme C [Felis catus] | 20275 | 1 | 1 | 1 | 1 | 6.1 | 9.27 | 0.26 |
| 273 | gi\|410980361\|ref\|XP_003996546.1\| | PREDICTED: vitronectin [Felis catus] | 57608 | 1 | 1 | 1 | 1 | 1.9 | 4.91 | 0.09 |
| 274 | gi\|410967138\|ref\|XP_003990079.1\| | PREDICTED: A/G-specific adenine DNA glycosylase isoform X4 [Felis catus] | 64141 | 1 | 1 | 1 | 1 | 1.3 | 9.38 | 0.08 |
| 275 | gi\|587020525\|ref\|XP_006943618.1\| | PREDICTED: FERM and PDZ domain-containing protein 4 [Felis catus] | 219196 | 3 | 0 | 1 | 0 | 0.4 | 5.36 |  |
| 276 | gi\|755794228\|ref\|XP_011287917.1\| | PREDICTED: transmembrane channel-like protein 4 [Felis catus] | 84403 | 1 | 1 | 1 | 1 | 1 | 9.43 | 0.06 |
| 277 | gi\|410964016\|ref\|XP_003988553.1\| | PREDICTED: ras association domain-containing protein 8 [Felis catus] | 57152 | 2 | 0 | 1 | 0 | 1.7 | 5.73 |  |
| 278 | gi\|755740690\|ref\|XP_011281318.1\| | PREDICTED: THAP domain-containing protein 10 [Felis catus] | 38918 | 1 | 1 | 1 | 1 | 2.1 | 8.03 | 0.13 |
| 279 | gi\|410950558\|ref\|XP_003981971.1\| | PREDICTED: calreticulin [Felis catus] | 58191 | 3 | 1 | 1 | 1 | 1.4 | 4.31 | 0.09 |
| 280 | gi\|410958730\|ref\|XP_003985967.1\| | PREDICTED: allograft inflammatory factor 1 isoform X1 [Felis catus] | 21402 | 1 | 1 | 1 | 1 | 4.1 | 7.85 | 0.25 |
| 281 | gi\|410947718\|ref\|XP_003980590.1\| | PREDICTED: ras GTPase-activating protein 3 [Felis catus] | 111640 | 4 | 0 | 1 | 0 | 0.7 | 7.71 |  |
| 282 | gi\|755814817\|ref\|XP_011290109.1\| | PREDICTED: transcriptional regulator ATRX [Felis catus] | 351021 | 2 | 0 | 1 | 0 | 0.3 | 6.37 |  |
| 283 | gi\|755702800\|ref\|XP_006929254.2\| | PREDICTED: CAS1 domain-containing protein 1 [Felis catus] | 100458 | 2 | 1 | 1 | 1 | 0.9 | 8.89 | 0.05 |
| 284 | gi\|410967474\|ref\|XP_003990244.1\| | PREDICTED: tetratricopeptide repeat protein 22 [Felis catus] | 69566 | 6 | 3 | 1 | 1 | 1 | 5.43 | 0.07 |
| 285 | gi\|586994145\|ref\|XP_003989618.2\| | PREDICTED: GDH/6PGL endoplasmic bifunctional protein isoform X2 [Felis catus] | 99508 | 3 | 2 | 1 | 1 | 0.7 | 7.97 | 0.05 |
| 286 | gi\|755765563\|ref\|XP_011284489.1\| | PREDICTED: rho guanine nucleotide exchange factor 26 [Felis catus] | 112591 | 3 | 0 | 1 | 0 | 0.7 | 8.94 |  |
| 287 | gi\|755695070\|ref\|XP_011286611.1\| | PREDICTED: fibrillin-3 [Felis catus] | 326166 | 3 | 0 | 2 | 0 | 0.4 | 5.07 |  |
| 288 | gi\|410959592\|ref\|XP_003986389.1\| | PREDICTED: RWD domain-containing protein 2A [Felis catus] | 39673 | 4 | 1 | 1 | 1 | 2.7 | 6.19 | 0.13 |
| 289 | gi\|755787771\|ref\|XP_011287174.1\| | PREDICTED: G protein pathway suppressor 2 [Felis catus] | 36163 | 3 | 2 | 1 | 1 | 2.8 | 9.59 | 0.14 |
| 290 | gi\|755761926\|ref\|XP_011284022.1\| | PREDICTED: pericentrin isoform X1 [Felis catus] | 425115 | 6 | 1 | 2 | 1 | 0.4 | 5.46 | 0.01 |
| 291 | gi\|586984768\|ref\|XP_006931174.1\| | PREDICTED: exocyst complex component 1 isoform X3 [Felis catus] | 120812 | 1 | 1 | 1 | 1 | 0.9 | 6.24 | 0.04 |
| 292 | gi\|755820195\|ref\|XP_011277747.1\| | PREDICTED: WD repeat-containing protein 44 isoform X1 [Felis catus] | 118600 | 3 | 1 | 1 | 1 | 0.7 | 5.28 | 0.04 |
| 293 | gi\|755705124\|ref\|XP_011279003.1\| | PREDICTED: prolactin-inducible protein [Felis catus] | 18882 | 2 | 0 | 1 | 0 | 4.8 | 8.8 |  |
| 294 | gi\|755729942\|ref\|XP_011280717.1\| | PREDICTED: dystonin isoform X1 [Felis catus] | 771116 | 6 | 1 | 3 | 1 | 0.3 | 5.66 | 0.01 |
| 295 | gi\|410954831\|ref\|XP_003984065.1\| | PREDICTED: coiled-coil domain-containing protein 85A isoform X1 [Felis catus] | 67662 | 3 | 1 | 1 | 1 | 1.1 | 9 | 0.07 |
| 296 | gi\|755703932\|ref\|XP_011278873.1\| | PREDICTED: thromboxane-A synthase [Felis catus] | 65632 | 4 | 4 | 1 | 1 | 1.1 | 7.17 | 0.08 |
| 297 | gi\|755756110\|ref\|XP_011283238.1\| | PREDICTED: ankyrin repeat domain-containing protein 13C [Felis catus] | 69913 | 2 | 0 | 1 | 0 | 1.1 | 6.43 |  |
| 298 | gi\|755716289\|ref\|XP_006930848.2\| | PREDICTED: probable ATP-dependent RNA helicase DDX60 [Felis catus] | 232855 | 1 | 1 | 1 | 1 | 0.5 | 7.85 | 0.02 |
| 299 | gi\|755688625\|ref\|XP_011278814.1\| | PREDICTED: progesterone-induced-blocking factor 1 [Felis catus] | 108313 | 2 | 0 | 1 | 0 | 1.2 | 5.8 |  |
| 300 | gi\|410967306\|ref\|XP_003990161.1\| | PREDICTED: carnitine O-palmitoyltransferase 2, mitochondrial isoform X1 [Felis catus] | 84010 | 4 | 1 | 1 | 1 | 0.9 | 8.8 | 0.06 |
| 301 | gi\|410972199\|ref\|XP_003992548.1\| | PREDICTED: transmembrane protein 225 [Felis catus] | 29161 | 1 | 1 | 1 | 1 | 2.6 | 9.43 | 0.18 |
| 302 | gi\|755779642\|ref\|XP_011286127.1\| | PREDICTED: ataxin-2 [Felis catus] | 132301 | 3 | 1 | 1 | 1 | 0.5 | 9.14 | 0.04 |
| 303 | gi\|410971087\|ref\|XP_003992005.1\| | PREDICTED: leucine-rich repeat-containing protein 31 [Felis catus] | 72521 | 2 | 0 | 1 | 0 | 1.2 | 5.65 |  |
| 304 | gi\|755759244\|ref\|XP_011283638.1\| | PREDICTED: LOW QUALITY PROTEIN: sodium channel protein type 7 subunit alpha [Felis catus] | 221934 | 2 | 0 | 1 | 0 | 0.5 | 8.56 |  |
| 305 | gi\|755802023\|ref\|XP_011288806.1\| | PREDICTED: kinesin-like protein KIF19 [Felis catus] | 113661 | 4 | 0 | 1 | 0 | 0.6 | 8.07 |  |
| 306 | gi\|755760416\|ref\|XP_011283807.1\| | PREDICTED: DBF4-type zinc finger-containing protein 2 [Felis catus] | 328705 | 4 | 1 | 3 | 1 | 0.7 | 5.56 | 0.01 |
| 307 | gi\|410953344\|ref\|XP_003983332.1\| | PREDICTED: zinc finger CCCH-type with G patch domain-containing protein [Felis catus] | 62578 | 3 | 0 | 1 | 0 | 1.4 | 5.5 |  |
| 308 | gi\|755726451\|ref\|XP_011280371.1\| | PREDICTED: zinc finger protein 391 isoform X2 [Felis catus] | 9084 | 2 | 2 | 1 | 1 | 7.6 | 9.3 | 0.66 |
| 309 | gi\|755741024\|ref\|XP_006932685.2\| | PREDICTED: spectrin beta chain, non-erythrocytic 5 [Felis catus] | 462168 | 5 | 2 | 1 | 1 | 0.2 | 6.11 | 0.01 |
| 310 | gi\|755688053\|ref\|XP_006927392.2\| | PREDICTED: LOW QUALITY PROTEIN: protein diaphanous homolog 3 [Felis catus] | 128521 | 3 | 0 | 1 | 0 | 0.6 | 6.2 |  |
| 311 | gi\|755688663\|ref\|XP_011278875.1\| | PREDICTED: glypican-6 [Felis catus] | 70073 | 1 | 1 | 1 | 1 | 1.8 | 5.3 | 0.07 |
| 312 | gi\|410985339\|ref\|XP_003998980.1\| | PREDICTED: transcription elongation factor B polypeptide 2 isoform X1 [Felis catus] | 15038 | 4 | 4 | 1 | 1 | 5.9 | 4.86 | 0.37 |
| 313 | gi\|755770815\|ref\|XP_011285107.1\| | PREDICTED: tripartite motif-containing protein 44 isoform X1 [Felis catus] | 43099 | 1 | 1 | 1 | 1 | 2.3 | 4.17 | 0.12 |
| 314 | gi\|755707496\|ref\|XP_003983653.3\| | PREDICTED: myosin-7B isoform X1 [Felis catus] | 262039 | 5 | 1 | 3 | 1 | 0.9 | 5.75 | 0.02 |
| 315 | gi\|587001225\|ref\|XP_006936792.1\| | PREDICTED: rho guanine nucleotide exchange factor 12 isoform X1 [Felis catus] | 196143 | 5 | 3 | 2 | 1 | 0.8 | 5.42 | 0.05 |
| 316 | gi\|410982486\|ref\|XP_003997587.1\| | PREDICTED: leukocyte receptor cluster member 1 [Felis catus] | 37413 | 8 | 4 | 2 | 1 | 4.3 | 10.01 | 0.14 |
| 317 | gi\|586999649\|ref\|XP_003991845.2\| | PREDICTED: tumor protein 63 isoform X1 [Felis catus] | 85783 | 1 | 1 | 1 | 1 | 0.9 | 6.05 | 0.06 |
| 318 | gi\|755782274\|ref\|XP_011286480.1\| | PREDICTED: sal-like protein 3 [Felis catus] | 146917 | 4 | 1 | 1 | 1 | 0.5 | 7.69 | 0.03 |
| 319 | gi\|410971047\|ref\|XP_003991985.1\| | PREDICTED: serpin I2 [Felis catus] | 53323 | 1 | 1 | 1 | 1 | 2 | 5.13 | 0.09 |
| 320 | gi\|283806558\|ref\|NP_001164535.1\| | multidrug resistance protein 1 [Felis catus] | 160813 | 3 | 1 | 1 | 1 | 0.5 | 8.94 | 0.03 |
| 321 | gi\|755758988\|ref\|XP_011283598.1\| | PREDICTED: low-density lipoprotein receptor-related protein 1B [Felis catus] | 585907 | 1 | 1 | 1 | 1 | 0.1 | 5.12 | 0.01 |
| 322 | gi\|410950616\|ref\|XP_003982000.1\| | PREDICTED: tRNA (guanine(26)-N(2))-dimethyltransferase isoform X1 [Felis catus] | 79398 | 2 | 2 | 1 | 1 | 0.9 | 8.53 | 0.06 |
| 323 | gi\|410970074\|ref\|XP_003991514.1\| | PREDICTED: uncharacterized protein C21orf62 homolog [Felis catus] | 25891 | 2 | 0 | 1 | 0 | 2.7 | 5.84 |  |
| 324 | gi\|410984840\|ref\|XP_003998733.1\| | PREDICTED: fructose-bisphosphate aldolase A [Felis catus] | 46108 | 1 | 1 | 1 | 1 | 1.9 | 8.3 | 0.11 |
| 325 | gi\|587011992\|ref\|XP_006940535.1\| | PREDICTED: EF-hand calcium-binding domain-containing protein 3 isoform X5 [Felis catus] | 62364 | 1 | 1 | 1 | 1 | 1.4 | 9.38 | 0.08 |
| 326 | gi\|410970677\|ref\|XP_003991804.1\| | PREDICTED: centrosomal protein of 19 kDa [Felis catus] | 23422 | 1 | 1 | 1 | 1 | 3.7 | 5.38 | 0.22 |
| 327 | gi\|755782697\|ref\|XP_006939134.2\| | PREDICTED: dedicator of cytokinesis protein 8 isoform X1 [Felis catus] | 263101 | 2 | 1 | 1 | 1 | 0.3 | 6.52 | 0.02 |
| 328 | gi\|755774848\|ref\|XP_006938022.2\| | PREDICTED: leucine-rich repeat-containing protein 18 [Felis catus] | 36616 | 1 | 1 | 1 | 1 | 2.3 | 9.83 | 0.14 |
| 329 | gi\|410981520\|ref\|XP_003997116.1\| | PREDICTED: probable ATP-dependent RNA helicase DDX5 [Felis catus] | 77639 | 2 | 1 | 1 | 1 | 1.3 | 9.06 | 0.06 |
| 330 | gi\|410975259\|ref\|XP_003994051.1\| | PREDICTED: putative hexokinase HKDC1 [Felis catus] | 120385 | 4 | 2 | 1 | 1 | 0.9 | 6.76 | 0.04 |
| 331 | gi\|755712248\|ref\|XP_006930294.2\| | PREDICTED: C-type lectin domain family 4 member F [Felis catus] | 75893 | 1 | 1 | 1 | 1 | 1.3 | 6.17 | 0.07 |
| 332 | gi\|755784893\|ref\|XP_011286805.1\| | PREDICTED: paralemmin-2 isoform X1 [Felis catus] | 54157 | 2 | 0 | 1 | 0 | 1.7 | 4.99 |  |
| 333 | gi\|586973679\|ref\|XP_006927406.1\| | PREDICTED: LOW QUALITY PROTEIN: protein aurora borealis [Felis catus] | 77878 | 2 | 0 | 1 | 0 | 1.1 | 5.54 |  |
| 334 | gi\|755708524\|ref\|XP_003983758.3\| | PREDICTED: attractin [Felis catus] | 174544 | 1 | 1 | 1 | 1 | 0.5 | 7.12 | 0.03 |
| 335 | gi\|755766639\|ref\|XP_003992321.2\| | PREDICTED: TPR and ankyrin repeat-containing protein 1 [Felis catus] | 394668 | 3 | 0 | 1 | 0 | 0.2 | 6.71 |  |
| 336 | gi\|587018401\|ref\|XP_006942909.1\| | PREDICTED: troponin I, slow skeletal muscle isoform X1 [Felis catus] | 32990 | 2 | 2 | 1 | 1 | 2.6 | 9.65 | 0.16 |
| 337 | gi\|755730616\|ref\|XP_011280806.1\| | PREDICTED: wiskott-Aldrich syndrome protein family member 1 isoform X1 [Felis catus] | 67598 | 1 | 1 | 1 | 1 | 0.9 | 6.01 | 0.08 |
| 338 | gi\|410959359\|ref\|XP_003986278.1\| | PREDICTED: transcription factor AP-2-delta [Felis catus] | 55216 | 1 | 1 | 1 | 1 | 1.5 | 8.41 | 0.09 |
| 339 | gi\|755758892\|ref\|XP_011283587.1\| | PREDICTED: RNA-binding protein 43 [Felis catus] | 49023 | 2 | 1 | 1 | 1 | 2 | 9.7 | 0.1 |
| 340 | gi\|410957577\|ref\|XP_003985402.1\| | PREDICTED: DNA-directed RNA polymerase II subunit RPB2 [Felis catus] | 150560 | 2 | 0 | 1 | 0 | 0.6 | 6.44 |  |
| 341 | gi\|755768333\|ref\|XP_011284819.1\| | PREDICTED: LOW QUALITY PROTEIN: 60S ribosomal protein L10-like [Felis catus] | 46318 | 1 | 0 | 1 | 0 | 1.7 | 9.32 |  |
| 342 | gi\|586996560\|ref\|XP_006935132.1\| | PREDICTED: bolA-like protein 1 isoform X2 [Felis catus] | 13575 | 4 | 4 | 1 | 1 | 5.5 | 10.81 | 0.41 |
| 343 | gi\|587011703\|ref\|XP_006940431.1\| | PREDICTED: histone-lysine N-methyltransferase EZH1 isoform X1 [Felis catus] | 102388 | 3 | 1 | 2 | 1 | 1.6 | 8.1 | 0.05 |
| 344 | gi\|410958760\|ref\|XP_003985982.1\| | PREDICTED: protein G6b isoform X1 [Felis catus] | 27515 | 2 | 0 | 1 | 0 | 2.5 | 9.54 |  |
| 345 | gi\|755738049\|ref\|XP_011281004.1\| | PREDICTED: ras-specific guanine nucleotide-releasing factor 1 [Felis catus] | 164710 | 1 | 1 | 1 | 1 | 0.6 | 6.91 | 0.03 |
| 346 | gi\|755762850\|ref\|XP_011284166.1\| | PREDICTED: enteropeptidase, partial [Felis catus] | 77374 | 1 | 1 | 1 | 1 | 1.3 | 5.21 | 0.06 |
| 347 | gi\|410987219\|ref\|XP_003999902.1\| | PREDICTED: tripartite motif-containing protein 55 isoform X1 [Felis catus] | 69554 | 1 | 1 | 1 | 1 | 1.1 | 4.66 | 0.07 |
| 348 | gi\|410958972\|ref\|XP_003986086.1\| | PREDICTED: protein kinase C and casein kinase substrate in neurons protein 1 [Felis catus] | 61558 | 2 | 0 | 1 | 0 | 2.3 | 5.14 |  |
| 349 | gi\|755755044\|ref\|XP_011283086.1\| | PREDICTED: MAP7 domain-containing protein 1 [Felis catus] | 101913 | 1 | 1 | 1 | 1 | 0.9 | 9.93 | 0.05 |
| 350 | gi\|410972786\|ref\|XP_003992837.1\| | PREDICTED: olfactory receptor 52M1 [Felis catus] | 37554 | 1 | 1 | 1 | 1 | 1.9 | 8.72 | 0.14 |
| 351 | gi\|586993676\|ref\|XP_006934150.1\| | PREDICTED: myosin-9 [Felis catus] | 268587 | 1 | 0 | 1 | 0 | 0.4 | 5.57 |  |
| 352 | gi\|755687951\|ref\|XP_011286060.1\| | PREDICTED: NEDD4-binding protein 2-like 1 [Felis catus] | 58665 | 2 | 1 | 1 | 1 | 1.2 | 9.52 | 0.08 |
| 353 | gi\|755696377\|ref\|XP_011288124.1\| | PREDICTED: zinc finger SWIM domain-containing protein 4 [Felis catus] | 133856 | 9 | 2 | 2 | 1 | 1 | 7.59 | 0.04 |
| 354 | gi\|755780792\|ref\|XP_011286271.1\| | PREDICTED: lethal(3)malignant brain tumor-like protein 4 [Felis catus] | 81125 | 2 | 0 | 1 | 0 | 1 | 6.4 |  |
| 355 | gi\|410986772\|ref\|XP_003999683.1\| | PREDICTED: histone-lysine N-methyltransferase ASH1L isoform X4 [Felis catus] | 398129 | 5 | 2 | 2 | 1 | 0.4 | 9.48 | 0.01 |
| 356 | gi\|755754437\|ref\|XP_011283020.1\| | PREDICTED: microtubule-actin cross-linking factor 1 [Felis catus] | 712983 | 3 | 0 | 2 | 0 | 0.2 | 5.24 |  |
| 357 | gi\|755745256\|ref\|XP_011281840.1\| | PREDICTED: centrosomal protein of 170 kDa protein B [Felis catus] | 166334 | 3 | 0 | 1 | 0 | 0.5 | 6.62 |  |
| 358 | gi\|410973352\|ref\|XP_003993117.1\| | PREDICTED: protein kinase C-binding protein NELL1 isoform X1 [Felis catus] | 102527 | 1 | 1 | 1 | 1 | 0.7 | 5.67 | 0.05 |
| 359 | gi\|755791635\|ref\|XP_011287593.1\| | PREDICTED: uncharacterized protein LOC102899173 [Felis catus] | 152842 | 6 | 2 | 1 | 1 | 0.5 | 5.38 | 0.03 |
| 360 | gi\|586976665\|ref\|XP_006928422.1\| | PREDICTED: zinc finger protein 317 isoform X1 [Felis catus] | 81552 | 1 | 1 | 1 | 1 | 1 | 9.3 | 0.06 |
| 361 | gi\|755691332\|ref\|XP_011282185.1\| | PREDICTED: transcription factor TFIIIB component B~~ homolog [Felis catus] | 333807 | 1 | 0 | 1 | 0 | 0.3 | 4.91 |  |
| 362 | gi\|755788035\|ref\|XP_011287215.1\| | PREDICTED: dynein heavy chain 9, axonemal [Felis catus] | 566522 | 7 | 1 | 1 | 1 | 0.2 | 5.82 | 0.01 |
| 363 | gi\|586991079\|ref\|XP_006933287.1\| | PREDICTED: CUGBP Elav-like family member 2 isoform X1 [Felis catus] | 58363 | 1 | 1 | 1 | 1 | 1.4 | 8.84 | 0.09 |
| 364 | gi\|755743958\|ref\|XP_011281688.1\| | PREDICTED: disks large-associated protein 5 [Felis catus] | 115256 | 1 | 0 | 1 | 0 | 0.7 | 9.07 |  |
| 365 | gi\|755759629\|ref\|XP_003990956.2\| | PREDICTED: coiled-coil domain-containing protein 141 [Felis catus] | 206769 | 1 | 0 | 1 | 0 | 0.7 | 5.51 |  |
| 366 | gi\|755754367\|ref\|XP_011283013.1\| | PREDICTED: adenylyl cyclase-associated protein 1 isoform X1 [Felis catus] | 61612 | 1 | 1 | 1 | 1 | 2.3 | 7.16 | 0.08 |
| 367 | gi\|586975312\|ref\|XP_006927946.1\| | PREDICTED: histone-lysine N-methyltransferase, H3 lysine-36 and H4 lysine-20 specific isoform X1 [Felis catus] | 354414 | 1 | 0 | 1 | 0 | 0.3 | 8.38 |  |
| 368 | gi\|755780409\|ref\|XP_006938807.2\| | PREDICTED: heparin cofactor 2 [Felis catus] | 66231 | 1 | 1 | 1 | 1 | 1.4 | 6.1 | 0.07 |
| 369 | gi\|410975411\|ref\|XP_003994126.1\| | PREDICTED: dual specificity protein phosphatase 13 isoform X6 [Felis catus] | 24260 | 1 | 0 | 1 | 0 | 4 | 9.54 |  |
| 370 | gi\|410947897\|ref\|XP_003980678.1\| | PREDICTED: putative transferase CAF17, mitochondrial [Felis catus] | 39124 | 2 | 2 | 1 | 1 | 1.5 | 8.53 | 0.13 |
| 371 | gi\|755701002\|ref\|XP_006929157.2\| | PREDICTED: biliverdin reductase A [Felis catus] | 39728 | 1 | 0 | 1 | 0 | 2.7 | 6.34 |  |
| 372 | gi\|755747923\|ref\|XP_011282186.1\| | PREDICTED: RNA polymerase II-associated protein 3 [Felis catus] | 93581 | 1 | 0 | 1 | 0 | 0.9 | 7.49 |  |
| 373 | gi\|410964571\|ref\|XP_003988827.1\| | PREDICTED: calcium-binding and coiled-coil domain-containing protein 1 isoform X2 [Felis catus] | 86463 | 3 | 0 | 1 | 0 | 1 | 4.79 |  |
| 374 | gi\|410983916\|ref\|XP_003998281.1\| | PREDICTED: conserved oligomeric Golgi complex subunit 4 [Felis catus] | 101298 | 1 | 0 | 1 | 0 | 1.3 | 5.09 |  |
| 375 | gi\|410958026\|ref\|XP_003985624.1\| | PREDICTED: cilia- and flagella-associated protein 99 [Felis catus] | 83909 | 1 | 0 | 1 | 0 | 0.9 | 9.24 |  |
| 376 | gi\|755759486\|ref\|XP_011283673.1\| | PREDICTED: LOW QUALITY PROTEIN: transcription factor Sp3 [Felis catus] | 82635 | 2 | 0 | 1 | 0 | 1 | 5.91 |  |
| 377 | gi\|410961455\|ref\|XP_003987298.1\| | PREDICTED: vam6/Vps39-like protein [Felis catus] | 118202 | 2 | 1 | 1 | 1 | 0.7 | 6.47 | 0.04 |
| 378 | gi\|755721092\|ref\|XP_011279944.1\| | PREDICTED: LOW QUALITY PROTEIN: uncharacterized protein LOC105260498 [Felis catus] | 70766 | 1 | 0 | 1 | 0 | 1.5 | 9.67 |  |
| 379 | gi\|410975523\|ref\|XP_003994180.1\| | PREDICTED: leucine-rich repeat, immunoglobulin-like domain and transmembrane domain-containing protein 2 [Felis catus] | 66705 | 3 | 3 | 1 | 1 | 1.3 | 6.24 | 0.07 |
| 380 | gi\|410973019\|ref\|XP_003992953.1\| | PREDICTED: putative olfactory receptor 56B2 [Felis catus] | 38638 | 1 | 0 | 1 | 0 | 2.5 | 8.06 |  |
| 381 | gi\|410953976\|ref\|XP_003983644.1\| | PREDICTED: microtubule-associated proteins 1A/1B light chain 3A [Felis catus] | 16612 | 1 | 1 | 1 | 1 | 5.8 | 8.74 | 0.33 |
| 382 | gi\|755775662\|ref\|XP_003994050.2\| | PREDICTED: LOW QUALITY PROTEIN: ATP-dependent RNA helicase SUPV3L1, mitochondrial [Felis catus] | 101347 | 1 | 1 | 1 | 1 | 1.1 | 8.3 | 0.05 |
| 383 | gi\|755695274\|ref\|XP_011286862.1\| | PREDICTED: histone-lysine N-methyltransferase, H3 lysine-79 specific [Felis catus] | 190788 | 1 | 0 | 1 | 0 | 0.6 | 9.44 |  |
| 384 | gi\|755792727\|ref\|XP_011287716.1\| | PREDICTED: ubiquitin carboxyl-terminal hydrolase 36 isoform X1 [Felis catus] | 140385 | 2 | 0 | 2 | 0 | 1.2 | 9.88 |  |
| 385 | gi\|410951323\|ref\|XP_003982347.1\| | PREDICTED: WD repeat-containing protein 82 [Felis catus] | 40956 | 2 | 1 | 1 | 1 | 1.9 | 7.59 | 0.12 |
| 386 | gi\|755742895\|ref\|XP_011281547.1\| | PREDICTED: disheveled-associated activator of morphogenesis 1 [Felis catus] | 146986 | 2 | 0 | 1 | 0 | 0.6 | 6.95 |  |
| 387 | gi\|755775779\|ref\|XP_011285671.1\| | PREDICTED: catenin alpha-3 [Felis catus] | 117282 | 1 | 1 | 1 | 1 | 0.8 | 6.02 | 0.04 |
| 388 | gi\|587015195\|ref\|XP_006941731.1\| | PREDICTED: cadherin-3 [Felis catus] | 100286 | 1 | 0 | 1 | 0 | 0.8 | 4.68 |  |
| 389 | gi\|755762692\|ref\|XP_006935959.2\| | PREDICTED: E3 ubiquitin-protein ligase TTC3 [Felis catus] | 270686 | 2 | 0 | 2 | 0 | 0.6 | 6.53 |  |
| 390 | gi\|410962441\|ref\|XP_003987778.1\| | PREDICTED: zinc finger and BTB domain-containing protein 1 isoform X1 [Felis catus] | 95251 | 1 | 0 | 1 | 0 | 0.8 | 5.97 |  |
| 391 | gi\|410965703\|ref\|XP_003989381.1\| | PREDICTED: aconitate hydratase, mitochondrial [Felis catus] | 99475 | 1 | 1 | 1 | 1 | 0.6 | 8.07 | 0.05 |
| 392 | gi\|587008128\|ref\|XP_006939179.1\| | PREDICTED: proprotein convertase subtilisin/kexin type 5 [Felis catus] | 118213 | 1 | 0 | 1 | 0 | 0.8 | 7.75 |  |
| 393 | gi\|587007386\|ref\|XP_006938924.1\| | PREDICTED: FH1/FH2 domain-containing protein 3 [Felis catus] | 194591 | 1 | 0 | 1 | 0 | 0.5 | 5.4 |  |
| 394 | gi\|410947306\|ref\|XP_003980391.1\| | PREDICTED: NHL repeat-containing protein 3 isoform X2 [Felis catus] | 42058 | 1 | 0 | 1 | 0 | 1.7 | 6.3 |  |
| 395 | gi\|410949682\|ref\|XP_003981548.1\| | PREDICTED: uncharacterized protein LOC101090745 [Felis catus] | 44250 | 2 | 2 | 1 | 1 | 1.4 | 8.69 | 0.13 |
| 396 | gi\|410980621\|ref\|XP_003996675.1\| | PREDICTED: E3 ubiquitin-protein ligase TRIM37 isoform X3 [Felis catus] | 120408 | 1 | 0 | 1 | 0 | 0.7 | 5.07 |  |
| 397 | gi\|410971130\|ref\|XP_003992026.1\| | PREDICTED: 1-phosphatidylinositol 4,5-bisphosphate phosphodiesterase eta-1 isoform X2 [Felis catus] | 214615 | 2 | 0 | 2 | 0 | 0.7 | 6.8 |  |
| 398 | gi\|410975976\|ref\|XP_003994403.1\| | PREDICTED: peroxisome proliferator-activated receptor gamma coactivator-related protein 1 isoform X1 [Felis catus] | 194568 | 1 | 0 | 1 | 0 | 0.5 | 5.63 |  |
| 399 | gi\|755754655\|ref\|XP_003989894.3\| | PREDICTED: zinc finger MYM-type protein 6 isoform X1 [Felis catus] | 177590 | 1 | 0 | 1 | 0 | 0.6 | 8.54 |  |
| 400 | gi\|586996488\|ref\|XP_006935109.1\| | PREDICTED: chromodomain-helicase-DNA-binding protein 1-like [Felis catus] | 116918 | 1 | 0 | 1 | 0 | 0.8 | 6.36 |  |
| 401 | gi\|410972253\|ref\|XP_003992575.1\| | PREDICTED: sialate O-acetylesterase isoform X1 [Felis catus] | 64503 | 1 | 1 | 1 | 1 | 1.2 | 8.49 | 0.08 |
| 402 | gi\|755790016\|ref\|XP_006940418.2\| | PREDICTED: probable ATP-dependent RNA helicase DHX58 [Felis catus] | 84149 | 1 | 0 | 1 | 0 | 1 | 8.16 |  |
| 403 | gi\|755743909\|ref\|XP_011281677.1\| | PREDICTED: F-box only protein 33, partial [Felis catus] | 59653 | 1 | 0 | 1 | 0 | 1.2 | 5.25 |  |
| 404 | gi\|755690699\|ref\|XP_003981008.2\| | PREDICTED: serine protease inhibitor Kazal-type 5 [Felis catus] | 150746 | 1 | 1 | 1 | 1 | 0.5 | 8.29 | 0.04 |
| 405 | gi\|755739199\|ref\|XP_006932626.2\| | PREDICTED: spermatogenesis-associated protein 5-like protein 1 [Felis catus] | 91114 | 2 | 0 | 1 | 0 | 0.9 | 8.3 |  |
| 406 | gi\|755761532\|ref\|XP_011283963.1\| | PREDICTED: obscurin-like protein 1 [Felis catus] | 196654 | 1 | 1 | 1 | 1 | 0.4 | 5.43 | 0.02 |
| 407 | gi\|410968693\|ref\|XP_003990836.1\| | PREDICTED: activin receptor type-1C [Felis catus] | 57565 | 1 | 0 | 1 | 0 | 2 | 8.34 |  |
| 408 | gi\|755806847\|ref\|XP_011289275.1\| | PREDICTED: integrator complex subunit 7 [Felis catus] | 119652 | 1 | 0 | 1 | 0 | 0.6 | 8.3 |  |
| 409 | gi\|410987883\|ref\|XP_004000224.1\| | PREDICTED: tonsoku-like protein isoform X2 [Felis catus] | 161220 | 1 | 0 | 1 | 0 | 0.6 | 6.28 |  |
| 410 | gi\|755767504\|ref\|XP_011284706.1\| | PREDICTED: complement C1q tumor necrosis factor-related protein 5 [Felis catus] | 28732 | 1 | 0 | 1 | 0 | 2.4 | 8.77 |  |
| 411 | gi\|325652164\|ref\|NP_001191707.1\| | cathelicidin antimicrobial peptide precursor [Felis catus] | 22453 | 1 | 0 | 1 | 0 | 3.5 | 6.37 |  |
| 412 | gi\|587006101\|ref\|XP_006938482.1\| | PREDICTED: kinetochore-associated protein 1 [Felis catus] | 289309 | 3 | 0 | 2 | 0 | 0.5 | 5.8 |  |
| 413 | gi\|410976788\|ref\|XP_003994795.1\| | PREDICTED: translational activator GCN1 [Felis catus] | 331854 | 1 | 0 | 1 | 0 | 0.3 | 7.18 |  |
| 414 | gi\|586994905\|ref\|XP_006934588.1\| | PREDICTED: brain-specific angiogenesis inhibitor 2 isoform X1 [Felis catus] | 185800 | 1 | 0 | 1 | 0 | 0.5 | 7.22 |  |
| 415 | gi\|586973408\|ref\|XP_006927316.1\| | PREDICTED: 28S ribosomal protein S31, mitochondrial isoform X1 [Felis catus] | 53371 | 1 | 0 | 1 | 0 | 1.8 | 9.04 |  |
| 416 | gi\|586985974\|ref\|XP_006931583.1\| | PREDICTED: mediator of DNA damage checkpoint protein 1 isoform X1 [Felis catus] | 230724 | 1 | 0 | 1 | 0 | 0.6 | 5.31 |  |
| 417 | gi\|410965178\|ref\|XP_003989127.1\| | PREDICTED: phosphatidylinositol phosphatase PTPRQ [Felis catus] | 280057 | 1 | 0 | 1 | 0 | 0.2 | 5.41 |  |
| 418 | gi\|587020273\|ref\|XP_006943518.1\| | PREDICTED: testis-specific serine/threonine-protein kinase 5-like [Felis catus] | 48135 | 1 | 1 | 1 | 1 | 1.7 | 9.47 | 0.1 |
| 419 | gi\|587017659\|ref\|XP_006942654.1\| | PREDICTED: nuclear valosin-containing protein-like isoform X1 [Felis catus] | 109135 | 1 | 0 | 1 | 0 | 0.8 | 7.23 |  |
| 420 | gi\|410953744\|ref\|XP_003983530.1\| | PREDICTED: beta-catenin-like protein 1 [Felis catus] | 73734 | 1 | 0 | 1 | 0 | 1.2 | 4.94 |  |
| 421 | gi\|410968705\|ref\|XP_003990842.1\| | PREDICTED: protein TANC1 isoform X1 [Felis catus] | 227505 | 1 | 0 | 1 | 0 | 0.3 | 8.67 |  |
| 422 | gi\|410978549\|ref\|XP_003995652.1\| | PREDICTED: Fanconi anemia group G protein [Felis catus] | 77698 | 1 | 0 | 1 | 0 | 1.1 | 5.25 |  |
| 423 | gi\|755760614\|ref\|XP_011283829.1\| | PREDICTED: protein FAM171B [Felis catus] | 104703 | 1 | 0 | 1 | 0 | 0.9 | 7.68 |  |
| 424 | gi\|755760290\|ref\|XP_011283783.1\| | PREDICTED: neurobeachin-like protein 1 [Felis catus] | 343166 | 1 | 0 | 1 | 0 | 0.3 | 5.87 |  |
| 425 | gi\|755781034\|ref\|XP_011286313.1\| | PREDICTED: LOW QUALITY PROTEIN: mothers against decapentaplegic homolog 7 [Felis catus] | 30076 | 1 | 0 | 1 | 0 | 2.5 | 6.79 |  |
| 426 | gi\|410964312\|ref\|XP_003988699.1\| | PREDICTED: olfactory receptor 8S1-like [Felis catus] | 37056 | 1 | 0 | 1 | 0 | 3 | 8.84 |  |
| 427 | gi\|587014929\|ref\|XP_006941625.1\| | PREDICTED: cyclic nucleotide-gated cation channel beta-1 [Felis catus] | 160648 | 4 | 0 | 2 | 0 | 1.1 | 4.62 |  |
| 428 | gi\|755777527\|ref\|XP_011285894.1\| | PREDICTED: LOW QUALITY PROTEIN: SEC23-interacting protein [Felis catus] | 125565 | 1 | 0 | 1 | 0 | 0.8 | 5.32 |  |
| 429 | gi\|587013184\|ref\|XP_006940975.1\| | PREDICTED: zinc finger protein 614 [Felis catus] | 80943 | 1 | 0 | 1 | 0 | 0.9 | 9.2 |  |
| 430 | gi\|755820169\|ref\|XP_011277745.1\| | PREDICTED: membrane-associated progesterone receptor component 1 [Felis catus] | 24709 | 1 | 0 | 1 | 0 | 4.1 | 4.56 |  |
| 431 | gi\|755770539\|ref\|XP_011285076.1\| | PREDICTED: activating molecule in BECN1-regulated autophagy protein 1 isoform X1 [Felis catus] | 146581 | 1 | 0 | 1 | 0 | 0.8 | 6.9 |  |
| 432 | gi\|410976099\|ref\|XP_003994463.1\| | PREDICTED: vesicle transport through interaction with t-SNAREs homolog 1A isoform X2 [Felis catus] | 27737 | 2 | 0 | 1 | 0 | 3.7 | 6.38 |  |
| 433 | gi\|755708501\|ref\|XP_011279236.1\| | PREDICTED: LOW QUALITY PROTEIN: leucine-rich repeat neuronal protein 4 [Felis catus] | 73788 | 1 | 1 | 1 | 1 | 0.9 | 8.05 | 0.07 |
| 434 | gi\|755759496\|ref\|XP_011283676.1\| | PREDICTED: low-density lipoprotein receptor-related protein 2 [Felis catus] | 580278 | 1 | 0 | 1 | 0 | 0.3 | 4.94 |  |
| 435 | gi\|755722538\|ref\|XP_011280022.1\| | PREDICTED: protein ZGRF1 [Felis catus] | 279343 | 1 | 0 | 1 | 0 | 0.4 | 6.18 |  |
