# Supplementary material for: Non-invasive identification of protein biomarkers for early pregnancy diagnosis in the cheetah (Acinonyx jubatus)
Source: PLoS One. 2017 Dec 13;12(12):e0188575. doi: 10.1371/journal.pone.0188575 (PMC5728495; doi:10.1371/journal.pone.0188575)
Supplement: S4 Table — Comparisons were made between samples from pregnant and non-pregnant (i.e., non-pregnant luteal phase and non-ovulatory) individuals. (DOCX) [file pone.0188575.s004.docx]

**S4 Table.**

| Protein –  reporter ions compared | Pregnant/non-pregnant luteal phase ratio | Pregnant/non-ovulatory control ratio |
| --- | --- | --- |
| Immunoglobulin J chain –  126/127N | 4.67 | 1.11* |
| Immunoglobulin J chain –  127C/128N | 1.52* | 3.19* |
| Immunoglobulin J chain –  128C/129N | 1.67* | 2.44 |
| Immunoglobulin J chain –  129C/130N | 2.66* | 1.79* |
| Immunoglobulin J chain –  130C/131 | 1.73* | 1.09 |
| Trefoil factor 3 –  126/127N | 5.67 | 0.94 |
| Trefoil factor 3 –  127C/128N | 1.38 | 1.39* |
| Trefoil factor 3 –  128C/129N | 3.06* | 3.72* |
| Trefoil factor 3 –  129C/130N | 5.75 | 1.28* |
| Trefoil factor 3 –  130C/131 | 1.18 | 2.06* |
| Nebulin –  126/127N | 3.61* | 1.08 |
| Nebulin –  127C/128N | 1.09 | 1.07 |
| Nebulin –  128C/129N | 2.06* | 1.82 |
| Nebulin –  129C/130N | 6.05* | 2.55* |
| Nebulin –  130C/131 | 1.56* | 1.08 |
| Complement C3 –  126/127N | 1.22* | 1.23* |
| Complement C3 –  127C/128N | 1.83 | 1.61* |
| Complement C3 –  128C/129N | 1.23 | 0.75 |
| Complement C3 –  129C/130N | 1.15 | 3.80* |
| Complement C3 –  130C/131 | 1.14 | 0.14 |
| Alkaline phosphatase –  126/127N | 0.83 | 0.76* |
| Alkaline phosphatase –  127C/128N | 0.73* | 1.00 |
| Alkaline phosphatase –  128C/129N | 0.71* | 0.80 |
| Alkaline phosphatase –  129C/130N | 0.83 | 0.89 |
| Alkaline phosphatase –  130C/131 | 1.56 | 0.97 |
| Myosin-binding protein C –  126/127N | 0.77* | 0.86* |
| Myosin-binding protein C –  127C/128N | 0.14 | 0.60* |
| Myosin-binding protein C –  128C/129N | 0.23* | 0.43* |
| Myosin-binding protein C –  129C/130N | 0.63 | 0.92 |
| Myosin-binding protein C –  130C/131 | 0.30 | 1.83 |

*Denotes significant (*P* < 0.05) expression change between pregnant and non-pregnant samples.
