# Supplementary material for: Non-invasive identification of protein biomarkers for early pregnancy diagnosis in the cheetah (Acinonyx jubatus)
Source: PLoS One. 2017 Dec 13;12(12):e0188575. doi: 10.1371/journal.pone.0188575 (PMC5728495; doi:10.1371/journal.pone.0188575)

S2 Appendix.

Immunoglobulin J chain

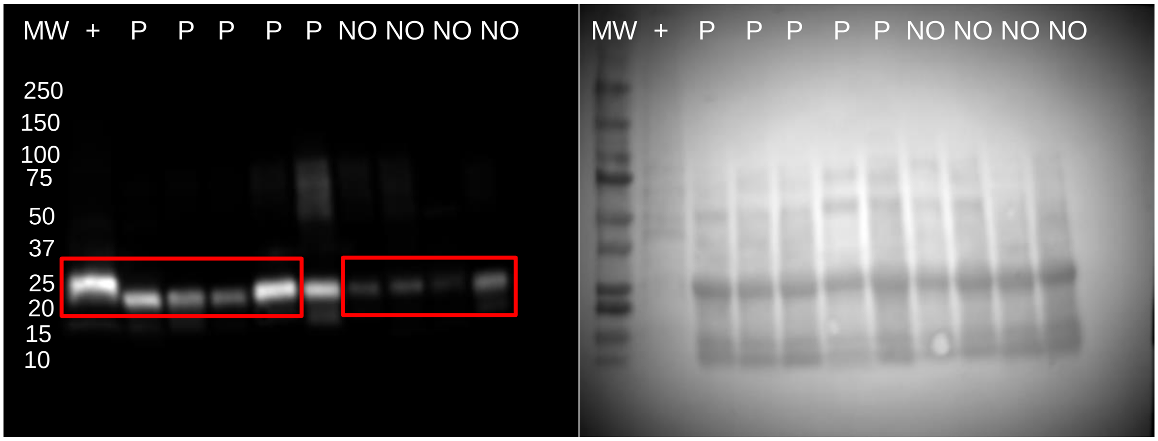

Immunoglobulin J chain

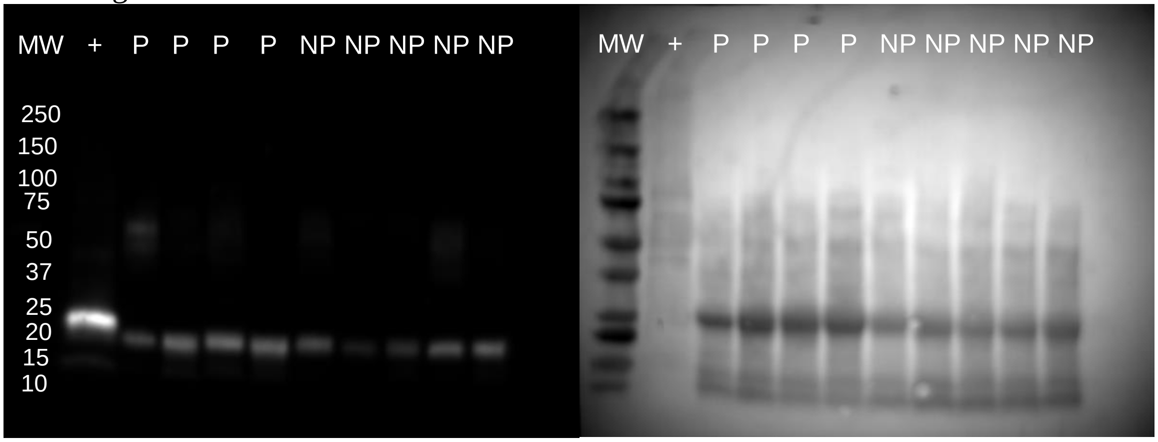

Immunoglobulin J chain

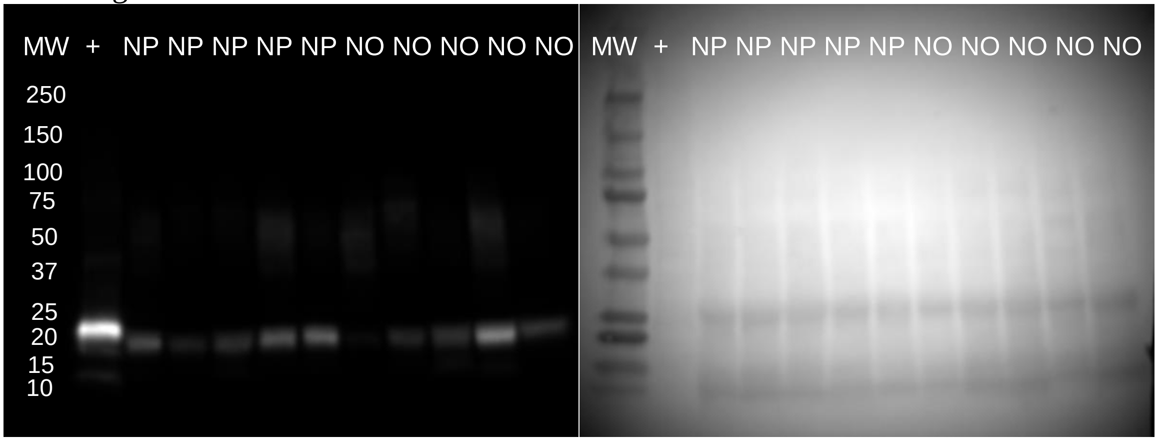

Immunoglobulin J chain

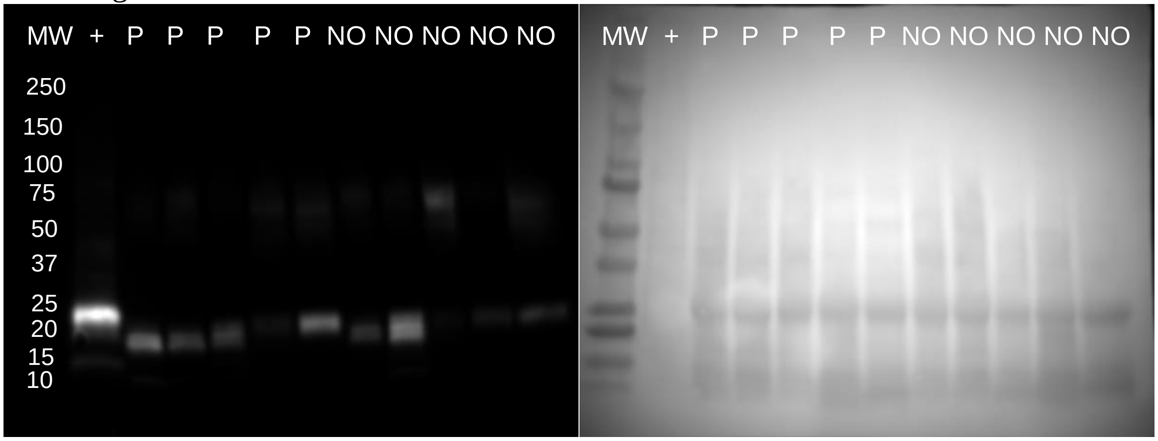

Immunoglobulin J chain

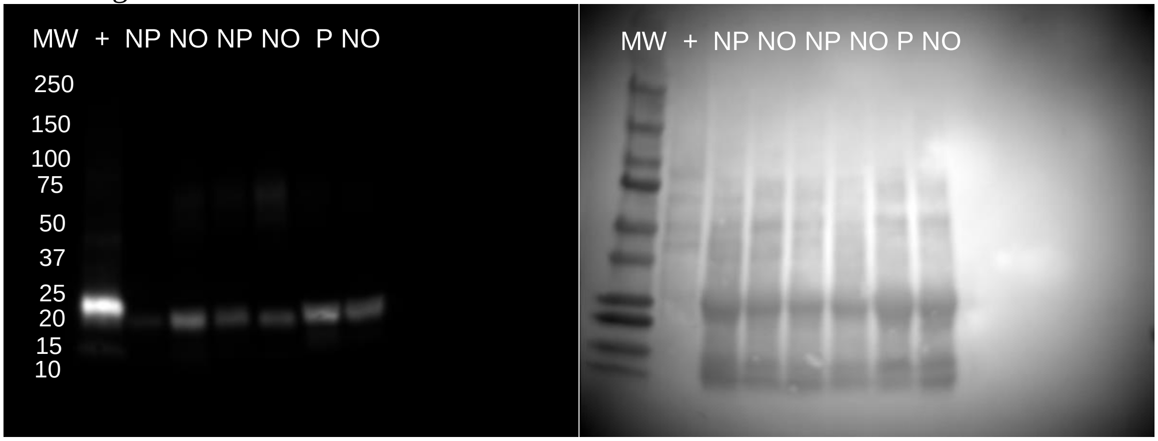

Trefoil factor 3

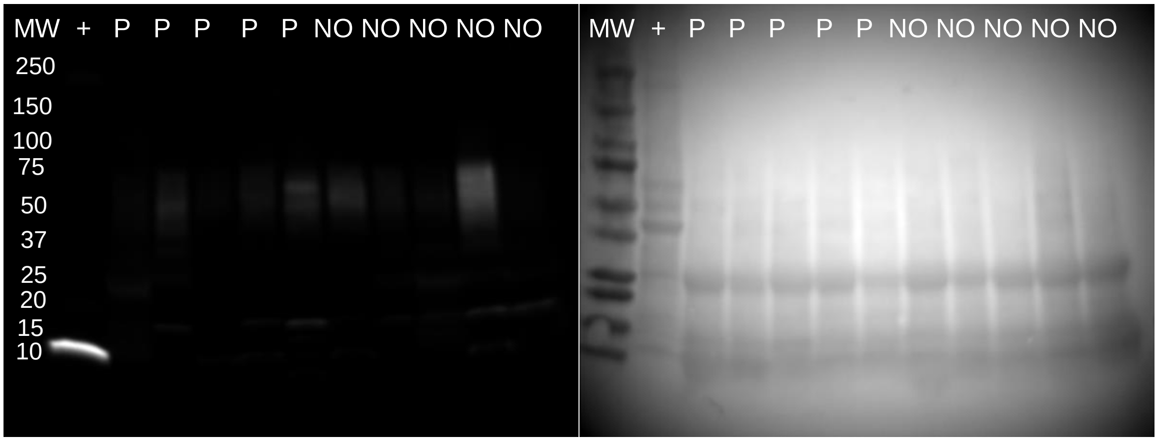

Trefoil factor 3

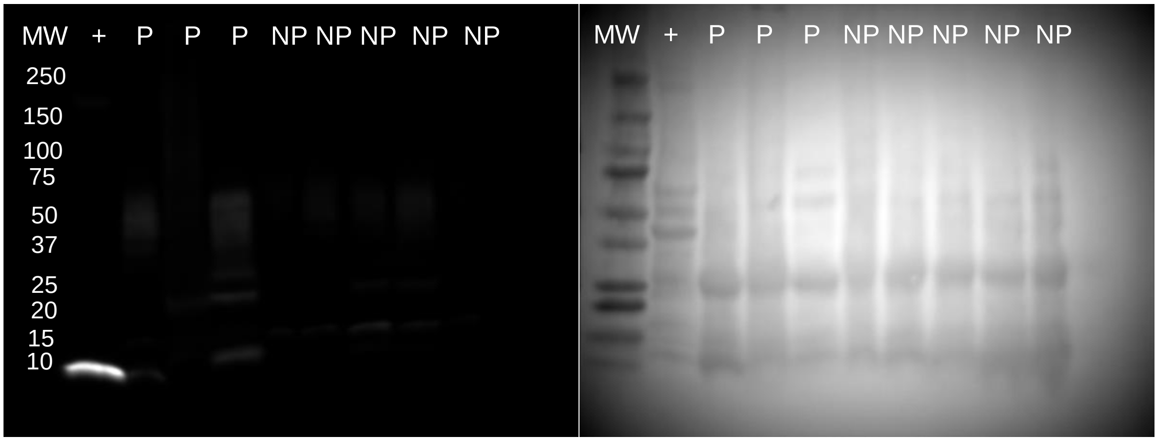

Myosin-binding protein C

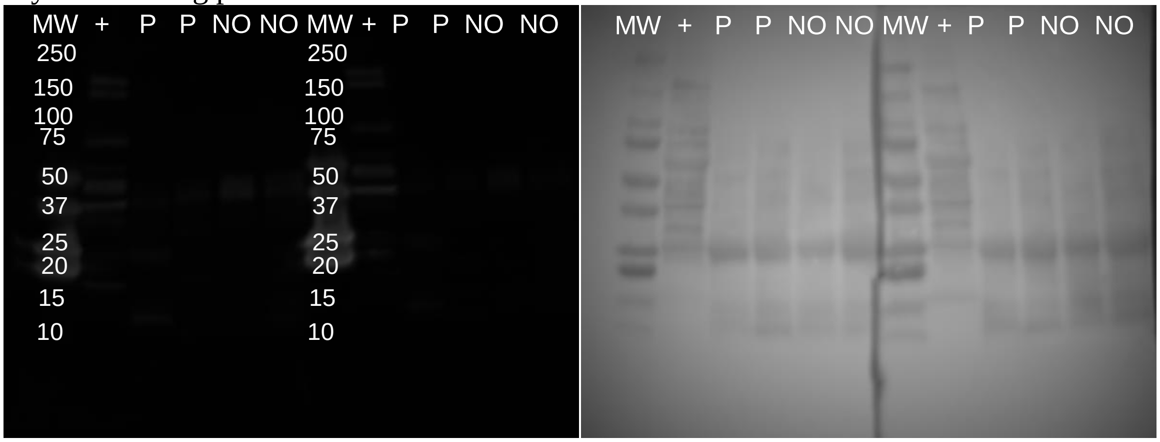

Myosin-binding protein C

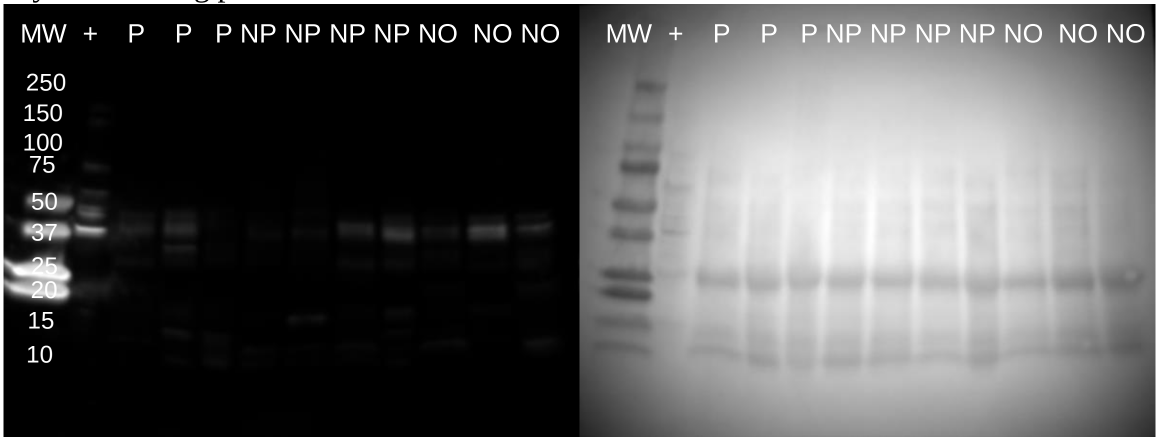

Myosin-binding protein C

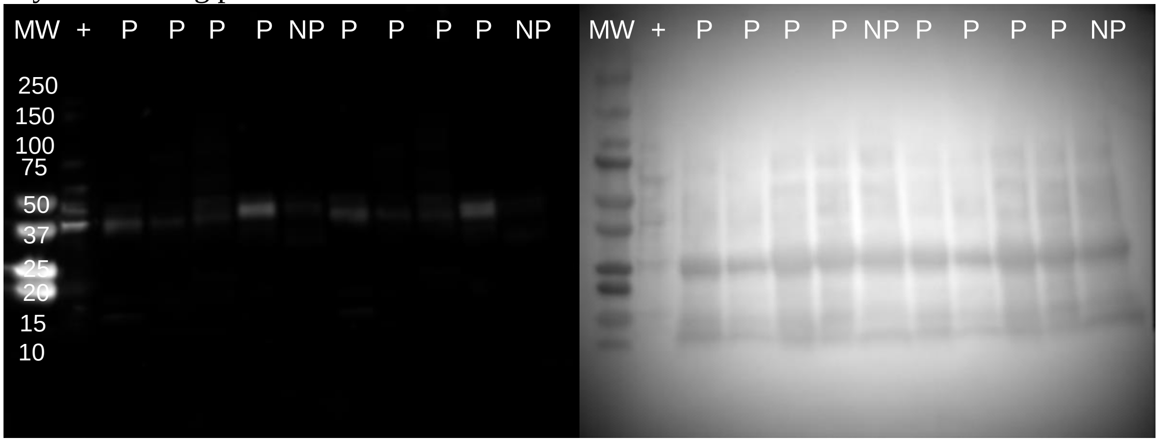

Complement C3

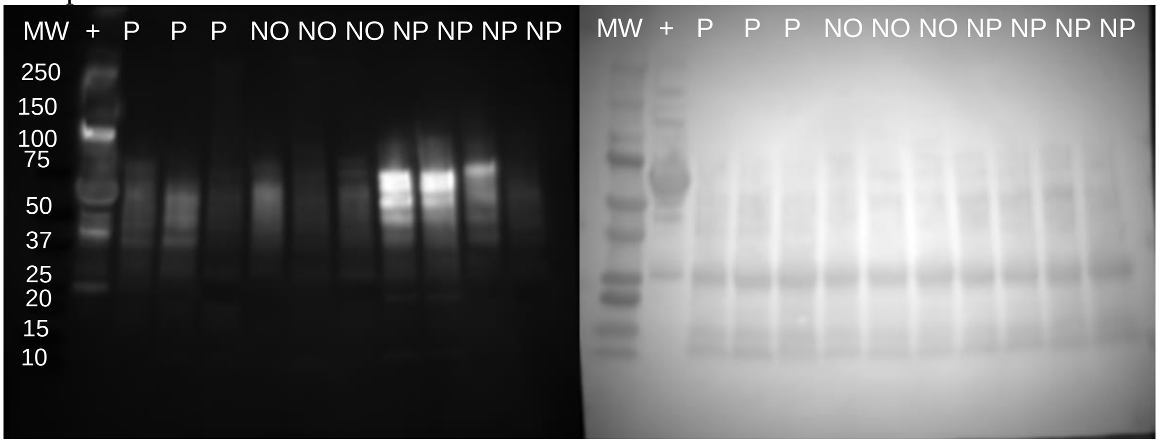

Complement C3

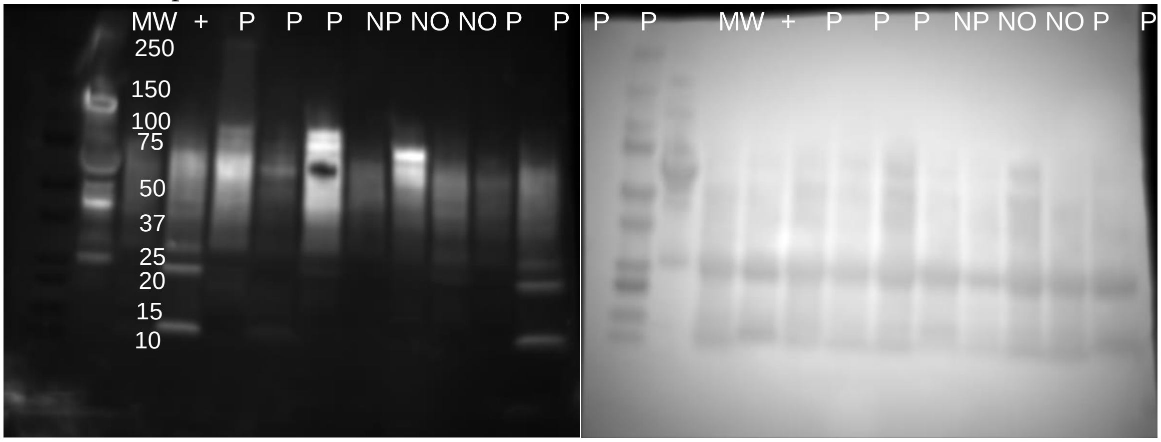

Alkaline phosphatase

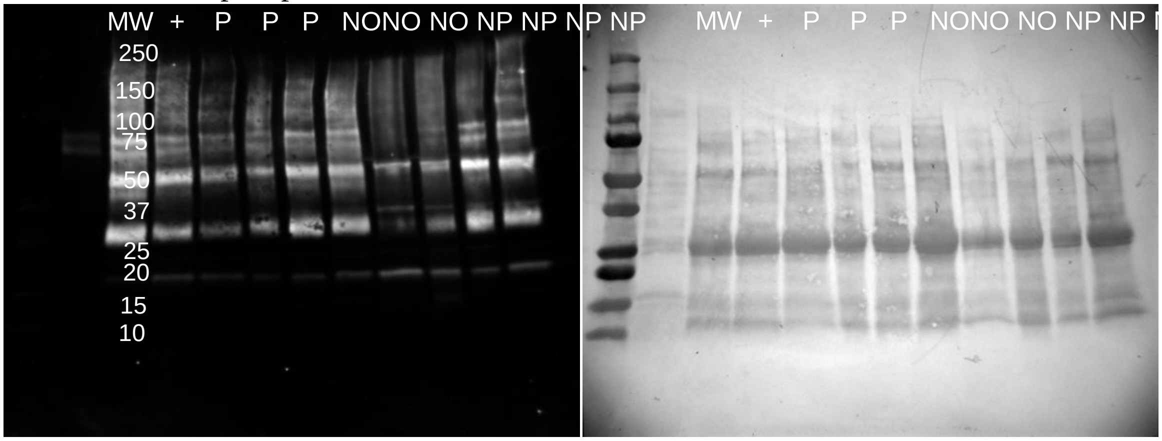

Alkaline phosphatase

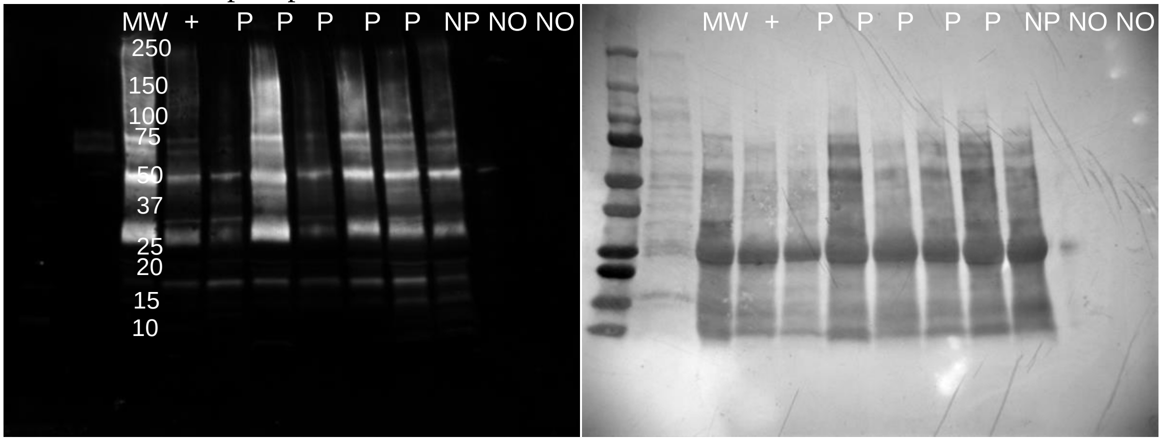

Supplement: S2 Appendix — Images utilized to generate protein intensity quantification. Each set of images represents chemiluminescence of specific antibody binding (left side) and corresponding light image (right side) to visualize molecular size markers (MW) and Coomassie stain of total lane protein as the loading control. Location of positive control (+), pregnant (P), non-pregnant luteal phase (NP), and non-ovulatory control (NO) samples are indicated at the top of each image. Red outline indicates blot sections shown in inset of Fig 5. (PDF) [file pone.0188575.s006.pdf]
